# Transcriptomic Analysis of the Effects of a Fish Oil Enriched Diet on Murine Brains

**DOI:** 10.1371/journal.pone.0090425

**Published:** 2014-03-14

**Authors:** Rasha Hammamieh, Nabarun Chakraborty, Aarti Gautam, Stacy-Ann Miller, Seid Muhie, James Meyerhoff, Marti Jett

**Affiliations:** United States Army Center for Environmental Health Research, Fort Detrick, Maryland, United States of America; Naval Research Laboratory, United States of America

## Abstract

The health benefits of fish oil enriched with high omega-3 polyunsaturated fatty acids (n-3 PUFA) are widely documented. Fish oil as dietary supplements, however, show moderate clinical efficacy, highlighting an immediate scope of systematic *in vitro* feedback. Our transcriptomic study was designed to investigate the genomic shift of murine brains fed on fish oil enriched diets. A customized fish oil enriched diet (FD) and standard lab diet (SD) were separately administered to two randomly chosen populations of C57BL/6J mice from their weaning age until late adolescence. Statistical analysis mined 1,142 genes of interest (GOI) differentially altered in the hemibrains collected from the FD- and SD-fed mice at the age of five months. The majority of identified GOI (∼40%) encodes proteins located in the plasma membrane, suggesting that fish oil primarily facilitated the membrane-oriented biofunctions. FD potentially augmented the nervous system's development and functions by selectively stimulating the Src-mediated calcium-induced growth cascade and the downstream PI3K-AKT-PKC pathways. FD reduced the amyloidal burden, attenuated oxidative stress, and assisted in somatostatin activation—the signatures of attenuation of Alzheimer's disease, Parkinson's disease, and affective disorder. FD induced elevation of *FKBP5* and suppression of *BDNF*, which are often linked with the improvement of anxiety disorder, depression, and post-traumatic stress disorder. Hence we anticipate efficacy of FD in treating illnesses such as depression that are typically triggered by the hypoactivities of dopaminergic, adrenergic, cholinergic, and GABAergic networks. Contrastingly, FD's efficacy could be compromised in treating illnesses such as bipolar disorder and schizophrenia, which are triggered by hyperactivities of the same set of neuromodulators. A more comprehensive investigation is recommended to elucidate the implications of fish oil on disease pathomechanisms, and the result-driven repositioning of fish oil utilization may revitalize its therapeutic efficacy.

## Introduction

Fish oils, derived from cold water oily fish like albacore tuna and salmon, are high in omega-3 poly-unsaturated fatty acids (n-3 PUFAs), mainly eicosapentaenoic acid (EPA) and docosahexaenoic acid (DHA), which are believed to have many health benefits [1]–[3]. N-3 PUFAs and fish oil, the major dietary source of n-3 PUFA have been extensively studied as dietary supplements suggesting beneficial effects for the treatment of inflammation [4]–[6], macular degeneration [7], [8], Alzheimer's disease (AD) [9], [10], Parkinson's disease (PD) [11]–[13] depression [6], [13], [14] and anxiety disorders [6], [15]. Optimum maintenance of the synaptosome, brain cell functions, and general health of human central nervous system are potentially facilitated by n-3 PUFA [16]–[18].

Despite of the wide implications of n-3 PUFA on the brain functions, clinical efforts investigating the efficacy of n-3 PUFAs and in that matter, the fish oil as dietary supplements for the treatment of psychiatric diseases have yet met with mixed results. Two separate clinical trials on Alzheimer's patients and Parkinson's patients supplemented by n-3 PUFA enriched diets failed to deliver a robust outcome. The trials found n-3 PUFA effective only if it was administered during the early onset of the AD [10]; in treating PD, its efficacy was restricted to mitigating the depression syndromes only [12]. N-3 PUFAs demonstrated significant positive impact on the major depressive episodes with anxiety co-morbidity [6], [13]; however, the supplement's efficacy on the heterogeneous disease profile (with and without comorbid anxiety) was marginal [13]. A pilot trial offering n-3 PUFA supplementation to patients immediately after life-threatening incidents resulted in reduced post traumatic syndromes [19].

A number of clinical, epidemiological, and laboratory studies linked the health benefits of n-3 PUFA to various dietary compositions, including EPA alone [20], [21], the EPA:DHA ratio [22], the arachidonic acid (AA):EPA ratio [23], [24] the AA: alpha-linolenic acid (ALA) ratio [25] and the overall n-3 PUFA; n-6 PUFA ratio [26]. Consequently, the appropriate dietary composition is still a subject of ongoing investigation.

Researchers have been using rodent models for decades to evaluate the efficacy of fish oil as the dietary supplement. Justifying the model, studies reported the increased levels of EPA and DHA accompanied by decreased AA in the brain tissues of rats fed on fish oil enriched diets [27], [28]. The n-3 PUFA enriched diet helped balancing behavioral plasticity [29], improved cognitive function [9] and working memory [30], enhanced neuroprotection [31], [32] and reduced anxiety and depression-like traits [33], [34] *in vitro*. High throughput genomic studies evaluating the effects of fish oil on the rodent brain identified genes involved in ion channels, neuronal function, signal transduction, synaptic plasticity, cytoskeleton and membrane association, and energy metabolism [35]–[41].

The comprehension of the underlying molecular mechanism is potentially critical to understand the effect of the fish oil on neuronal activities. Toward this goal, we studied the effects of high dietary intake of fish oil on the genomic regulations in the mouse brain, and characterized the potentially associated molecular events focused on the nervous system and neurofunctions. The effects of FD on the genes relevant to neurogenesis are of particular interest in the context of the recent study manifesting n-3PUFA as the mediators in generating new neuronal cells [42].

## Materials and Methods

### 2.1 Ethics statement

Research was conducted in compliance with the Animal Welfare Act, and other Federal statutes and regulations relating to animals and experiments involving animals and adheres to principles stated in the Guide for the Care and Use of Laboratory Animals (NRC 2011) in facilities that are fully accredited by the Association for the Assessment and Accreditation of Laboratory Animal Care, International. The protocol was approved by the IACUC committee of Walter Reed Army Institute of Research, Silver Spring, MD.

### 2.2 Diet types and mouse handling

The percentage of the fish oil in the customized FD was restricted to 16% to maintain the food pellet structure consistent with that of SD (Certified Rodent Diet 5002*, Purina LabDiet, MA). Thus, the external appearance and texture of FD and SD appeared similar. Solid food pellets helped maintaining the tidiness of the cages; hence, the cages occupied by FD- or SD-fed mice followed the same schedule of regular lab handling, precluding any concerns associated with psychological bias due to the handling with variable frequency [43]. Possible bias due to the rodents' potential favoring of particular food textures was further averted by introducing the two diets with similar textures.

Briefly, these two diets were similarly supplemented by proteins (FD: 18.3%, SD: 20.7%, both by weight), carbohydrates (FD: 53.8%, SD: 51.8%, by weight) and crude fibers (FD: 4%, SD: 4.3%, by weight); vitamins and minerals were also supplemented proportionately including vitamin E (FD: 90 IU/kg; SD: 65 IU/kg). The different supplements of fat (FD: 16%; SD: 5.5%, both by weight), primarily the higher n-3 PUFA supplements in FD (FD: 4.70%; SD: 0.20%, by weight) outlined the major difference between FD and SD. The n-6 PUFA supplements were marginally lower in FD (FD: 0.66%; SD: 1.22%, by weight). Overall caloric contributions from the two diets were comparable (FD: 4.2 Kcal/g; SD: 4.07 Kcal/g) (Table 1). To maintain the food quality across the study duration and to minimize the oxidative damage to n-3 PUFA, freshly prepared food was purchased every two months and stored at 4°C in sealed bags and the food chambers were replenished daily with fresh supplies.

**Table 1:** Compositions of the primary nutrients and n-3 PUFA and n-6 PUFA in two diets of interest.

		Fish diet (FD)		Standard diet (SD)	
		% by weight	% kcal	% by weight	% kcal
**Primary Nutrients**					
**Protein**		18.3	17.2	23.9	24.11
**Carbohydrate**		51.8	48.8	53.8	62.67
**Fat**		16.0	33.9	5.7	13.22
***Kcal/g***		***4.2***		***4.07***	
**Polyunsaturated Fatty Acids**					
**n-3 PUFA**	**EPA**	2.56		0.20	
	**DHA**	1.73			
	**ALA**	0.29			
**n-6 PUFA**	**LA**	0.29		1.22	
	**AA**	0.37		<0.01	
***n-3 PUFA∶n-6 PUFA***		***7∶1***		***1∶6***	
***EPA∶DHA***		***1.5∶1***		***0***	

The composition of protein, carbohydrate, and fat present in the fish oil enriched diet (FD) and standard diet (SD). The percent fraction by the weight and the calories offered by each diet type is presented. Total calories (Kcal/g) offered by each diet type is noted. The percentage fractions of three n-3 PUFA (EPA, DHA, and ALA) and two n-6 PUFA (AA and LA) are reported.

In designing the assay, we refrained from adjusting any potential shortcomings of the fish oil. For instance FD diet contained 0.3% linoleic acid (LA), whose scale of representation in rodent diets has long been a subject of investigation [44], [45]; of note the nearly equi-caloric control diet contained 1.22% LA (Table 1), as per the recommendation [44]. Hereby, our result highlighted the diet-induced molecular events potentially explaining the suggested benefits or the adverse effects of fish oil on the brain.

C57BL/6J male mice were purchased from Jackson Laboratory (Bar Harbor, ME) at the age of three weeks, singly housed and immediately introduced to either SD or FD. Mice had free access to their respective diets and standard lab-supplied liquids for next five months (∼20 weeks).

### 2.3 Collection of hemibrains and RNA isolation

Following the euthanization of the mice by cervical dislocation the brains were removed from their crania. After the mid-sagittal section, the hemibrains were collected by a highly trained professional experienced with this procedure, who was kept blinded from the animal identities during the dissection period. The dissected brain sections were snap-frozen, saved in individually labeled tubes, and transferred to the freezer at the end of the day. The entire process starting with the brain removal from the skull to the snap-freezing of the last brain region took less than 20 minutes as described in our earlier report [46].

On the day of nucleic acid isolation, organs were weighed before thawing and the appropriate amounts of Trizol™ (Invitrogen, NY) were added. RNA was isolated from the Trizol™ suspensions as per the manufacturer's guidelines. Briefly, the hemibrain was homogenized using the TissueLyser system (Qiagen, MD), phase separated with vigorous agitation, and the aqueous phase was collected. Serial alcohol-based precipitations and washes were performed on the aqueous phase to obtain RNA. The isolated nucleic acids were quantified and qualified using the Agilent BioAnalyzer as reported earlier [47].

### 2.4 High throughput transcriptomic expression analysis

The dual dye microarray was carried out using the Whole Mouse Genome Microarray Kit (Agilent Technologies, Inc.) following the vendor's protocol. 200–2000 ng of purified RNA was labeled with Cy-5 dyes and the reference RNA (Agilent, CA) with Cy-3 dyes (N = 4 from FD-fed mice and N = 3 from SD-fed mice. One SD sample was misplaced). The samples were simultaneously hybridized to Agilent 4×44k slides (platform number: 14868) and incubated for 17 h at 55°C. After overnight hybridization, slides were processed in a series of washes. The slides were scanned using an Agilent DNA microarray scanner and the features were extracted using the default setting of the Feature Extraction software (Feature Extraction software v.10.7, Agilent, CA).

### 2.5 Data filtering and process normalization

GeneSpring v.10.1 (Agilent Technologies, Inc., CA) was used to carry out the preliminary data filtration and statistical analysis. Each chip was subjected to intra-chip normalization using the locally weighted scatter-plot smoothing method (LOWESS) [48]. Genes were identified that were differently expressed between the hemibrains of mice fed on FD vs. SD based on ±1.5 fold change cut-off with FDR<0.05.

The microarray data was submitted to the Gene Expression Omnibus (GEO). This can be searched using the Platform ID: GPL7202, Series: GSE46933.

### 2.6 Cluster and functional analysis

**Figure 1:**
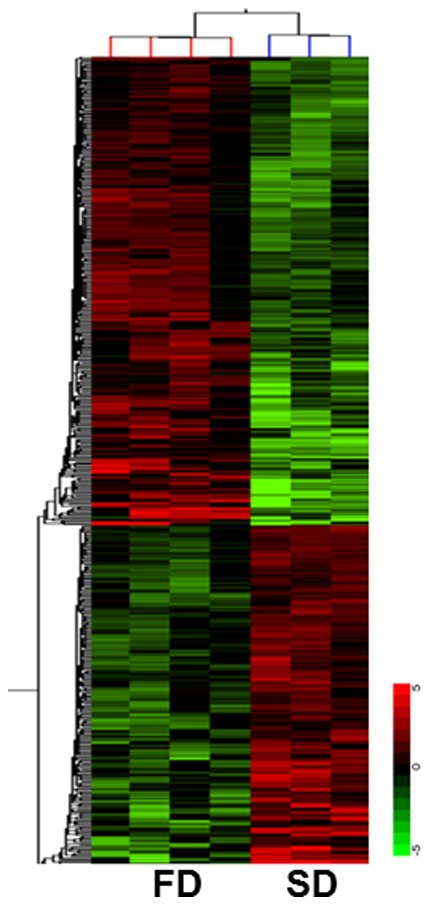
Cluster view (heatmap) of 1,142 genes differentially expressed between the mice fed with fish oil enriched diet (FD) vs. standard lab diet (SD). These genes are identified using a cut-off of ±1.5 fold changes between the mice with FD vs. SD, with FDR<0.05, and clustered using Pearson correlation algorithm. The color scale of the fold change (log_1.5_ (FD-/SD-fed mice) is noted at the bottom right of the figure. The first three columns from left represent the genomic expressions of 4 animals fed on FD and subsequent three columns of 3 animals fed on SD.

GeneSpring v.10.1 (Agilent Technologies, Inc., CA) was used to perform two-dimensional hierarchal clustering using Pearson correlation algorithm (Figure 1).

For the gene ontological classification and enrichment analysis, FATIGO^+^ [49], MSigDB-Parametric Geneset Enrichment analysis (http://www.broadinstitute.org/gsea/msigdb/index.jsp) and GeneCite [50] were used. Ingenuity Pathway Analysis (IPA; www.ingenuity.com) was used to mine the pathways and networks relevant to the present study.

### 2.7 Real-time PCR validation of microarray results

The mRNA microarray results were validated using two platforms. The high throughput real-time PCR was carried out using the RT^2^ Profiler PCR Array System (SABiosciences, MD) on the ABI 7900HT platform (Life Technologies, CA). The PCR was carried out using three biological replicates for each diet. SDS 2.4 software supplied by ABI, CA, was used to compute the changes of threshold cycles (Ct) (*i.e.*, 2 ∧ (−Δ Ct), where Δ Ct = Ct (GOI)−mean (Ct (HKG)); GOI denotes the gene of interest, and HKG denotes the housekeeping gene. In order to eliminate the false positives, the mRNA reads were screened based on two criteria: (i) the controls' threshold cycles were >30, and samples' threshold cycles were <30 (or vice versa), and (ii) the *p*-values for the fold-changes were either unavailable or relatively high (*p*>0.05) from the assay backgrounds.

In addition, qPCR of five genes (*NOS1*, *FKBP5*, *MMP9*, *SOD1* and *BDNF*) was carried out using primers purchased from Fisher Scientific, Inc, PA using their Solaris gene expression assay (Table S1). The selection of these genes is justified in a brief literature survey (Table 2). Real-time PCR was performed in the HT700 platform (Applied BioSystems, CA) using five technical replicates. The downstream analysis was conducted using the GAPDH (glyceraldehyde-3-phosphate dehydrogenase) and beta-actin housekeeping genes following the protocol described elsewhere [47].

**Table 2:** A brief literature survey of clinical studies associated with the panel of genes validated by qPCR.

Gene Name	Trauma	Cohort	Major findings	Reference
NOS-1	Depression	Snap-frozen and paraffin-embedded tissue of anterior cingulate cortex from Caucasian subjects; major depressive disorder, *N* = 5; bipolar disorder, *N* = 7; and control *N* = 12	Diminished NOS1 in anterior cingulate cortex with depression, possibly by affecting glutamatergic and GABAergic neurotransmission	[120]
	AD	European subjects; AD (baseline, *N* = 19; 30 months follow-up, *N* = 95; 60 months follow-up, *N* = 106) and controls (baseline, *N* = 555; 30 months follow-up, *N* = 360; 60 months follow-up, *N* = 285)	Decreased NOS1 expression in concert with degeneration of NOS-I neurons likely results in impaired hippocampal nitrinergic neurotransmission.	[121]
	Schizophrenia	Japanese patient; Postmortem brain samples of 12 schizophrenic patients, *N* = 12; and control, *N* = 15	Decreased NOS-1 is associated with schizophrenia patients; with stronger correlation for female patient	[122]
	Schizophrenia/Bi-polar disorder	Post mortem brain samples from schizophrenic patients *N* = 26; bipolar patients *N* = 30; and controls, *N* = 29	Over-expression of specific NOS1 isoforms among the schizophrenia patients, but not among bipolar patients	[123]
MMP9	Traumatic brain injury (TBI)	TBI patient, *N* = 7; and control, *N* = 4; all male	Increased levels of MMP-9/-3 in the ventricular CSF of patients with severe TBI.	[124]
	Bipolar depression	Patients with bipolar disorder (*N* = 54); and controls (*N* = 29)	Increased serum MMP-9 during depression in young patients	[125]
	AD	AD patients (*N* = 38); and controls (*N* = 34)	Higher MMP-9/TIMP-1 ratios and lower TIMP-1 levels compared to cognitively healthy individuals.	[126]
	AD/PD	AD patients (*N* = 30); PD patients (*N* = 24); and controls (*N* = 32)	Elevated MMP-9 in the plasma of AD patients; but no significant changes of MMP-9 levels in PD cohorts.	[127]
	Schizophrenia	Schizophrenic patients (*N* = 442); and controls (*N* = 558)	Significant preponderance of −1562 C:T polymorphism in patients	[128]
SOD-1	AD	Post-mortem brain samples from AD patient, *N* = 4; and patients diagnosed with Huntington's Disease, *N* = 4	Decreased SOD-1 in AD patients' hippocampus	[129]
	Down syndrome (DS) and AD	Post-mortem brain samples; DS, *N* = 9, AD, *N* = 9; and controls, *N* = 9	Increased SOD-1 in DS temporal, parietal, and occipital cortex, whereas decreased SOD-1 in the AD temporal cortex	[130]
BDNF	PTSD	All women cohort, diagnosed with PTSD, *N* = 17; with PTSD+ childhood physical neglect, *N* = 17; controls, *N* = 15	Lower plasma BDNF in association with PTSD, and more strongly linked with childhood neglect driven PTSD	[131]
	PTSD	Patients with history of trauma, *N* = 34; and controls, *N* = 34	Higher level of serum BDNF levels right after the traumatic events decreasing over time	[84]
	Bipolar disorder	UK Caucasian subjects, BD, *N* = 962; and control, *N* = 2100	No overall influence from the Val66Met polymorphism of BDNF, with a moderate association with the susceptibility to the rapid-cycling subset of the disorder.	[132]
	Major depression	Korean subjects, major depression, *N* = 310 and controls, *N* = 209	Significant association of BDNF with the disease onset among the younger subgroup only	[133]
	PD	PD patients, *N* = 453 and controls, *N* = 291	Increased PD risk in association with the pesticide exposure	[134]
	AD	AD patients with rapid cognitive decline, *N* = 12; and slow cognitive decline, *N* = 28	Decreased BDNF serum levels in AD patients with fast cognitive decline	[135]
FKBP5	PTSD	Nonpsychiatric clinic patients with significant levels of childhood abuse and non–child abuse trauma, N = 900	High interaction between four SNPs of the FKBP5 gene (rs9296158, rs3800373, rs1360780, and rs9470080) with severity of child abuse as a predictor of adult PTSD	[136]
	PTSD	Subjects linkage and association studies of the genetics of cocaine, opioid, and alcohol dependence, and controls; N = 1146 European Americans (EAs) and 1284 African Americans (AAs)	*AA subjects:* Significant association between one SNP (rs9470080) with childhood abuse eliciting PTSD. *EA subjects:* Significant association of PTSD risk with alcohol dependence and childhood adverse experiences interacting with FKBP5 polymorphisms	[137]
	Anxiety/depression	Patients, newly diagnosed with advanced gastric cancer and supposed to receive first-line chemotherapy, *N* = 130; and control similar patients after two cycles of chemotherapy, *N* = 93	Significant association of anxiety with FKBP5 rs9296158, and marginal association with rs9470080 and rs1360780. Marginal association of depression with FKBP5 rs9470080 and rs9296158	[138]
	Major depression	Patients diagnosed with major depression, *N* = 68; and controls, *N* = 87	Significant interaction between disease status and FKBP5 risk allele carrier status (minor allele T).	[139]

Past clinical reports (demography of the cohorts and major findings) associated with five genes (*NOS-1*, *MMP-9*, *SOD-1*, *BDNF* and *FKBP5*) are reported.

## Results

The diet compositions listed in Table 1 revealed relatively comparable supplements of major nutrients and consumable calories derived from FD and SD. The major differences between the two diet types were attributed to the n-3 PUFA and n-6 PUFA supplements (Table 1).

The mice weighing 10–12 grams at the age of three weeks had a 61.2% increase in body weight as a result of consuming SD for five months; thus the average weight became 30.96 grams (% CV = 6.12). During the same five month period, FD-fed mice showed 67.2% increase in body weight; the average weight became 36.6 grams (% CV = 13.17). It was a near significant difference in the change of body weights between the FD- vs. SD-fed mice, with the FD-fed group gaining 18% more in body mass (*p* = 0.06).

Transcriptomic analysis identified 1,142 genes altered between the mice fed on FD vs. SD (6.6% of total genes). The GOI demonstrated ±1.5 fold changes with FDR<0.05. Figure 1 is a cluster view of these GOI generated using the Pearson correlation algorithm; 659 (58% of GOI) and 483 (42% of GOI) transcripts were elevated and suppressed by FD, respectively. One hundred and fifty two unknown genes with the remaining 990 known genes are documented in Table S2. Figure S1 shows a volcano plot of the same gene set. The entire list is available in the public domain: Gene Expression Omnibus (GEO) and this can be using the Platform ID: GPL7202, Series: GSE46933.

Meeting the objectives of the present study, subsequent convergent functional analysis identified the transcripts linked to the *neurogenesis, neuroviability and neurodegenerative disorders*.

### 3.1. Biofunction analysis

The 1,142 GOI enriched an ensemble of biofunctions. Table 3 reports the top-ranked biofunctions (*p*<0.0001) that are enriched by at least 50 genes. The complete list of biofunctions is shown in the Table S3. We identified four biofunctions pertinent to the present objective: “Neurological disease”, “Nervous system development and function”, “Inflammatory response” and “Cell death”, and these were further classified into subcategories enriched by at least 30 genes. Of note, we found certain enriched pathways, such as “LPS/IL-1 Mediated Inhibition of RXR Function”, “Amyotrophic Lateral Sclerosis Signaling” and “Hepatic Fibrosis/Hepatic Stellate Cell Activation”; however, we failed to derive biologically meaningful information from these pathways. Therefore, we focused on exploring the four biofunctional modules identified herein. Together, these four biofunctions enlisted 247 genes (153 up- and 94 down-regulated by FD). The majority of the genes (99 genes, i.e. 40% of the 247 genes) encode the proteins that are located in the plasma membrane. Curating from the Allen Brain Atlas (mouse.brain-map.org) we linked the 20% of these 247 genes to possible brain regions (Table S4).

**Table 3:** Significant biofunctions (p<0.0001) enriched by more than 50 candidates from the 1,142 genes of interest (GOI).

Biofunction Categories	p-values	Number of Molecules
Genetic Disorder	1.09E-3 to 4.24E-6	432
Neurological Disease	1.01E-3 to 5.69E-5	260
Movement disorder	8.13E-04	93
Neuropathy	2.43E-03	88
Motor neuron disease	1.58E-03	86
Progressive motor neuropathy	1.98E-03	85
Encephalopathy	1.79E-03	85
Neurodegenerative disorder	4.50E-04	76
Alzheimer's disease	1.11E-03	70
Parkinson's disease	1.26E-03	50
Spinal cord disorder	1.89E-04	30
Gastrointestinal Disease	1.61E-3 to 1.37E-4	232
Metabolic Disease	1.73E-3 to 2.27E-6	211
Tissue Development	1.04E-3 to 1.79E-7	202
Immunological Disease	1.07E-3 to 9.63E-5	193
Cellular Growth and Proliferation	1.61E-3 to 5.90E-5	184
Cardiovascular Disease	1.03E-3 to 8.17E-6	180
Endocrine System Disorders	1.73E-3 to 4.57E-5	180
Cellular Development	1.06E-3 to 3.03E-7	179
Cell-To-Cell Signaling and Interaction	1.04E-3 to 1.46E-11	176
Molecular Transport	1.01E-3 to 3.48E-6	169
Hematological Disease	1.03E-3 to 1.18E-5	165
Nervous System Development and Function	1.04E-3 to 7.83E-7	150
Neurogenesis	5.91E-06	73
Neurological process of cells	1.01E-07	51
Neurological process of neurons	7.83E-07	42
Neurotransmission	2.41E-07	37
Synaptic transmission	4.98E-07	35
Development of brain	2.42E-04	33
Neurotransmission of nervous tissue	2.54E-06	30
Organismal Development	1.73E-3 to 2.23E-5	149
Cellular Movement	1.61E-3 to 7.36E-4	123
Embryonic Development	1.54E-3 to 2.23E-5	118
Hematological System Development and Function	1.04E-3 to 6.18E-6	112
Inflammatory Disease	1.60E-3 to 2.06E-5	111
Tissue Morphology	1.44E-3 to 3.96E-5	109
Inflammatory Response	3.03E-3 to 6.18E-6	108
Immune response	1.14E-04	102
Activation of leukocytes	7.25E-05	50
Activation of mononuclear leukocytes	4.61E-05	39
Activation of lymphocytes	1.49E-04	36
Activation of T lymphocytes	7.59E-05	30
Small Molecule Biochemistry	1.10E-3 to 2.10E-4	107
Organ Development	2.19E-3 to 2.23E-5	101
Dermatological Diseases and Conditions	1.73E-3 to 9.63E-5	98
Cell Signaling	1.01E-3 to 3.48E-6	97
Cell Death	1.25E-3 to 1.79E-7	89
Apoptosis	3.03E03 to 8.95E-4	43
Killing	7.62E-4 to 3.03E-7	23
Cytotoxicity	3.03E-3 to 7.98E-5	23
Immune Cell Trafficking	1.04E-3 to 6.18E-6	80
Developmental Disorder	1.72E-3 to 2.66E-3	79
Vitamin and Mineral Metabolism	1.01E-3 to 4.04E-6	75
Lipid Metabolism	1.06E-3 to 2.24E-4	75
Behavior	1.17E-3 to 3.19E-5	74
Skeletal and Muscular Disorders	1.26E-3 to 5.10E-4	67
Organismal Injury and Abnormalities	1.73E-3 to 1.89E-8	65
Connective Tissue Development and Function	1.27E-3 to 3.76E-5	55
Cardiovascular System Development and Function	1.73E-3 to 6.54E-5	55
Skeletal and Muscular System Development and Function	1.58E-3 to 3.76E-5	52

Subcategories enriched by >30 genes associated with the “neurological diseases”, “nervous development and functions”, “inflammatory response” and “cell death” are elaborated.

#### 3.1.i. Neurological diseases

**Figure 2:**
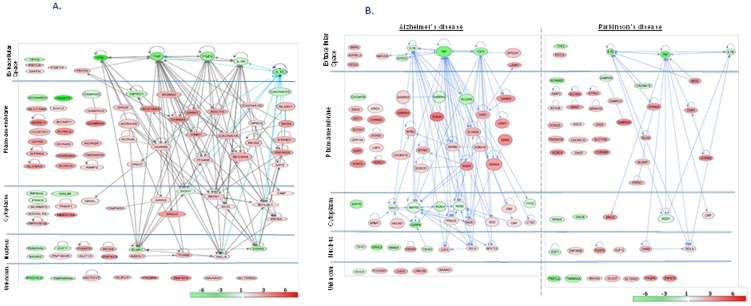
Regulatory networks showing the clusters of differentially regulated genes involved in “neurological diseases”. The nodes represent the genes and the solid lines depict the interactions between two molecules. Red- and green-colored genes show significantly increased and decreased expression (log_1.5_ (FD-/SD-fed mice) in the FD-fed mice compared to the SD-fed mice (a color scale is at the bottom right corner). The genes are clustered based on their proteins' cellular locations in the nucleus, cytoplasm, plasma membrane and extracellular space. The genes without the location information are grouped under ‘unknown’. *Figure 2A* depicts “movement disorder”, the most enriched disease enlisting 70 genes with *p* = 8.13×10^−4^. *Figure 2B* depicts two additional diseases frequently linked with n-3 PUFA diets. Alzheimer's disease (left panel) and Parkinson's disease (right panel) enlist 70 and 50 genes with *p* values of 1.11×10^−3^ and 1.26×10^−3^, respectively.

Two hundred and sixty genes (22.7% of GOI) were associated with the “neurological diseases” with a range of *p* values from 1.01×10^−3^ to 5.96×10^−5^ (Table 3). In particular, we focused on three diseases: (i) movement disorder (MvD) (93 genes, *p* = 8.13×10^−4^) (Figure 2A, Tables 3 and 4), (ii) AD (70 genes, *p* = 1.11×10^−3^) (Figure 2B, Tables 3 and 4) and (iii) PD (50 genes, *p* = 1.26×10^−3^) (Figure 2B, Tables 3 and 4). The movement disorder was the most enriched candidate on the list. We also selected AD and PD for further discussion in light of the suggested implications of n-3 PUFAs on these particular illnesses [9], [10], [12], [51]. Together, these three diseases explained approximately 34% of the transcripts linked to “neurological diseases”, with 16 common genes among three disease types of interest. In addition, 3 transcripts were shared by MvD and AD, and 14 transcripts by MvD and PD.

**Table 4:** The genomic candidates associated with three top-ranked neurological diseases: movement disorder (MvD), Alzheimer's disease (AD) and Parkinson's disease (PD).

Entrez ID	Gene Symbol	Log Ratio	Neurological Disease
16521	KCNJ5	1.45	AD
20660	SORL1	1.32	AD, MvD
14397	GABRA4	1.27	AD, MvD, PD
15360	HMGCS2	1.24	MvD
20508	SLC18A3	1.20	MvD
108068	GRM2	1.18	AD
99738	KCNC4	1.17	AD, MvD, PD
18053	NGFR	1.15	AD
76829	DOK5	1.13	AD
214779	ZNF879	1.12	MvD, PD
12671	CHRM3	1.11	AD, MvD, PD
108043	CHRNB3	1.11	AD, MvD, PD
12661	CHL1	1.10	AD
108073	GRM7	1.05	AD, MvD, PD
94187	ZNF423	1.05	MvD
20618	SNCG	1.03	MvD, PD
68957	PAQR6	1.02	MvD, PD
17441	MOG	1.01	MvD, PD
140741	GPR6	1.01	MvD
110893	SLC8A3	0.98	MvD, PD
14811	GRIN2A	0.98	AD, MvD, PD
16504	KCNC3	0.97	MvD
16772	LAMA1	0.96	AD
114142	FOXP2	0.96	MvD, PD
72605	CA10	0.94	AD
224129	ADCY5	0.92	MvD
12801	CNR1	0.88	AD, MvD, PD
26380	ESRRB	0.86	AD
71137	RFX4	0.85	MvD
63993	SLC5A7	0.84	AD
213262	FSTL5	0.83	AD, MvD, PD
140919	SLC17A6	0.83	MvD, PD
18213	NTRK3	0.80	AD, MvD, PD
15567	SLC6A4	0.80	AD, MvD
16536	KCNQ2	0.78	MvD
19049	PPP1R1B	0.78	MvD
12161	BMP6	0.75	AD
407831	TMEM204	0.74	MvD, PD
19281	PTPRT	0.73	AD, MvD, PD
20191	RYR2	0.73	AD
21834	THRB	0.71	MvD, PD
226922	KCNQ5	0.69	MvD, PD
218763	LRRC3B	0.69	AD
192167	NLGN1	0.68	MvD, PD
230777	HCRTR1	0.67	MvD
18610	PDYN	0.67	MvD
239133	DLEU7	0.66	MvD, PD
12286	CACNA1A	0.66	MvD
23859	DLG2	0.64	MvD, PD
17172	ASCL1	0.64	MvD
213783	PLEKHG1	0.64	AD
72844	KCTD17	0.62	MvD
70357	KCNIP1	0.62	MvD
20745	SPOCK1	0.60	AD
13492	DRD5	0.59	AD, MvD, PD
67703	KIRREL3	0.59	AD
16493	KCNA5	0.59	MvD
14169	FGF14	0.56	MvD
269132	GLT25D2	0.56	MvD, PD
11550	ADRA1D	0.54	AD, MvD, PD
18125	NOS1	0.54	AD, MvD, PD
21955	TNNT1	0.53	MvD
12950	HAPLN1	0.53	AD
12289	CACNA1D	0.52	AD, MvD, PD
16785	RPSA	0.52	MvD
319924	APBA1	0.51	AD
269513	NKAIN3	0.50	AD, MvD, PD
16409	ITGAM	0.49	MvD
94253	HECW1	0.49	AD
12265	CIITA	0.49	AD
11555	ADRB2	0.47	AD, MvD
14407	GABRG3	0.46	AD, MvD, PD
14407	GABRG3	0.46	MvD
58178	SORCS1	0.45	AD
12411	CBS	0.44	AD
12336	CAPNS1	0.44	MvD
12048	BCL2L1	0.44	MvD
435965	LRP3	0.44	AD
16597	KLF12	0.43	MvD, PD
170735	ARR3	0.43	MvD
14708	GNG7	0.43	MvD, PD
241494	ZNF385B	0.43	MvD, PD
16522	KCNJ6	0.41	MvD, PD
29856	SMTN	0.40	MvD
231872	AIMP2	0.39	MvD, PD
56807	SCAMP5	0.39	MvD
54218	B3GALT4	0.37	MvD
14680	GNAL	0.36	MvD
20249	SCD	0.35	AD, MvD
13033	CTSD	0.35	AD
19697	RELA	0.34	AD, MvD, PD
14804	GRID2	0.32	AD, MvD
18753	PRKCD	0.31	AD
12799	CNP	0.30	AD, MvD, PD
17528	MPZ	0.29	MvD
18019	NFATC2	0.27	AD
244431	SGCZ	0.25	MvD, PD
241263	GPR158	0.24	AD
20655	SOD1	−0.32	AD, MvD, PD
16439	ITPR2	−0.33	AD
110796	TSHZ1	−0.34	AD
26419	MAPK8	−0.35	AD
110886	GABRA5	−0.37	AD, MvD, PD
19108	PRKX	−0.37	MvD
239743	KLHL6	−0.39	AD
16176	IL1B	−0.44	AD, MvD, PD
20102	RPS4X	−0.45	MvD, PD
12292	CACNA1S	−0.47	AD, MvD, PD
243931	TSHZ3	−0.47	AD
11813	APOC2	−0.50	AD
17536	MEIS2	−0.50	MvD
50790	ACSL4	−0.51	AD
68272	RBM28	−0.52	AD
52679	E2F7	−0.55	MvD, PD
18386	OPRD1	−0.60	MvD
19366	RAD54L	−0.61	MvD
13653	EGR1	−0.61	MvD
319625	GALM	−0.65	MvD, PD
72519	TMEM55A	−0.67	MvD, PD
13654	EGR2	−0.69	MvD
117591	SLC2A9	−0.71	AD
435726	KCNMB3	−0.79	MvD, PD
77055	KRT76	−0.81	AD
21785	TFF2	−0.85	MvD, PD
14173	FGF2	−0.87	AD, MvD
12363	CASP4	−0.96	AD
16153	IL10	−0.97	MvD, PD
252973	GRHL2	−1.03	AD
76645	PKD1L2	−1.05	MvD, PD
21926	TNF	−1.21	AD, MvD, PD
14686	GNAT2	−1.94	MvD
19109	PRL	−2.23	MvD

The genes are identified by the Entrez ID and gene symbol. The log ratio indicates the log_1.5_ transformed of the average transcriptomic expression from FD-/SD-fed mice.

FD elevated the expression of a substantial subset of transcripts associated with MvD (76%), AD (58%) and PD (74%). Proteins associated with 45 transcripts representing 48% of MvD associated genes are located in membranes; likewise, 42% (29 transcripts) and 54% (27 transcripts) of the proteins related to AD and PD, respectively, are located in the membranes.

A comprehensive FD-induced elevation was observed among the transcripts associated with the calcium and potassium channels (*CACNA1A*, *CACNA1D*, *KCNMB3*, *KCNQ5*, *KCN1P1*, *KCNC4*, *KCNC3*, *KCNA5*, *KCNJ6*, *KCTD7* and *KCNQ2*) except one, namely *CACNA1S*. All transcriptomic members of solute carrier family (*SLC18A3*, *SLC5A7* and *SLC6A4*), and dopamine, glutamate, adrenergic and cholinergic receptors (*DRD5*, *GRID2*, *GRIN2A*, *GRM2*, *GRM7*, *ADRAID*, *ADRB2*, *CHRM3* and *CHRNB3*) were also elevated. Alternatively, FD reduced the expression of the transcripts associated with the cytokines (*IL1B*, *IL10*, *PRL* and *TNF*) and early growth response agents (*EGR1* and *EGR2*). The members of zinc finger family (*ZNF385B* and *ZNF423*) displayed over expression, but those of the teashirt zinc finger family (*TSHZ1* and *TSHZ2*) displayed suppressed expression in the FD-fed mice. Transcripts encoding the G-protein coupled receptor (*GPR6*) were elevated, but that encoding the ionotropic GABA receptors were differentially expressed. FD inhibited the transcripts associated with the domain of alpha 5 (*GABRA5*) and elevated alpha 4 (*GABRA4*), respectively. Although *GABRG3* and *GABRA5* are phenotypically juxtaposed in human chromosome, FD elevated the former and suppressed the later transcript.

#### 3.1.ii. Nervous system development and function

**Figure 3:**
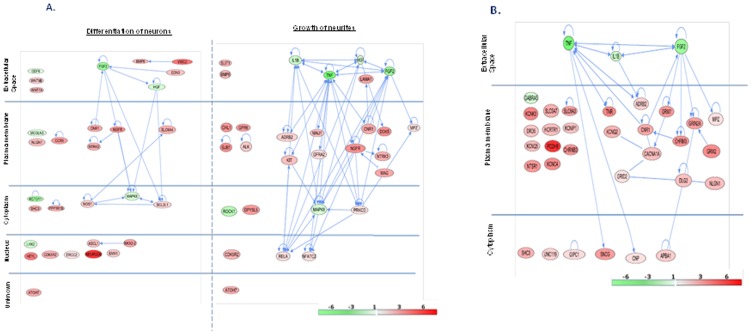
Regulatory networks showing the clusters of differentially-regulated genes involved in “nervous development and functions”. The nodes represent the genes and the solid lines depict the interactions between two molecules. Red- and green-colored genes show significantly increased and decreased expression (log_1.5_ (FD-/SD-fed mice) in the FD-fed mice compared to the SD-fed mice (a color scale is at the bottom right corner). The genes are clustered based on their proteins' cellular locations in the nucleus, cytoplasm, plasma membrane, and extracellular space. The genes without the location information are grouped under ‘unknown’. *Figure 3A* depicts the two top-ranked subcategories of “neurogenesis”. “Differentiation of neurons” (left panel) and “growth of neurites” (right panel) enlist 30 and 29 genes with *p* values of 1.7×10^−31^ and 2.3×10^−24^, respectively. *Figure 3B* depicts “synaptic transmission”, the most significant nervous function. It enlists 34 genes, *p* = 4.9×10^−7^.

One hundred and fifty genes (13.13% of GOI) were associated with the “nervous system development and function” with a range of *p* values from 2.92×10^−3^ to 1.01×10^−7^ (Table 5). Neurogenesis (Ngs) was the most enriched function; 73 transcripts represented 48.6% of all the members associated with the parent function term. Further investigation of Ngs associated genes identified five of the most significantly enriched functional sub-categories, which enlisted 62 genes representing 85% of all genes associated with Ngs. These sub-families were (i) differentiation of neurons (DoN) (30 genes, *p* = 1.7×10^−31^) (Figure 3A, Table 5), (ii) growth of neurites (GoN) (29 genes, *p* = 2.3×10^−24^) (Figure 3A, Table 5), (iii) differentiation of the nervous system (DNS) (29 genes, *p* = 7.6×10^−23^) (Figure S2, Table 5), (iv) formation of plasma membrane projections (FPMP) (26 genes, *p* = 4.71×10^−19^) (Figure S2, Table 5) and (v) neuritogenesis (Ntg) (23 genes, p = 8.02×10^−18^) (Figure S2, Table 5). Among these five closely interconnected sub-families, DoN and GoN collectively enlisted the largest number of unique transcripts (50, 68.5% of the genes associated with Ngs). Therefore, we focused primarily on these two functional sub- categories (Figure 3A).

**Table 5:** The genomic candidates associated with two top-ranked “nervous system development and functions”: neurogenesis (Ngs) and the synaptic transmission (SyT).

Entrez ID	Gene symbol	Log Ratio	Nervous system development and functions
11922	NEUROD6	2.22	Ngs (Ntg, FPMP, DCNS, DoN)
18530	PCDH8	2.12	SyT
211134	LZTS1	1.80	Ngs (Ntg, FPMP, DCNS)
319922	VWC2	1.43	Ngs (DCNS, DoN)
56198	HEYL	1.35	Ngs (DoN)
21960	TNR	1.24	SyT
20508	SLC18A3	1.20	Ngs (Ntg, FPMP)
108068	GRM2	1.18	SyT
14618	GJB1	1.15	Ngs (GoN, DCNS)
18053	NGFR	1.15	Ngs (Ntg, FPMP, GoN, DCNS, DoN)
76829	DOK5	1.13	Ngs (GoN)
12671	CHRM3	1.11	SyT
108043	CHRNB3	1.10	SyT
12661	CHL1	1.10	Ngs (Ntg, FPMP, DCNS, DoN)
18216	NTSR1	1.07	SyT
108073	GRM7	1.05	SyT
20618	SNCG	1.03	SyT
65254	DPYSL5	1.02	Ngs (GoN)
140741	GPR6	1.01	Ngs (GoN)
12774	CCR5	0.99	Ngs (DoN)
110893	SLC8A3	0.98	SyT
14811	GRIN2A	0.98	SyT
16772	LAMA1	0.96	Ngs (Ntg, GoN, FPMP)
18088	NKX2-2	0.93	Ngs (DCNS, DoN)
17136	MAG	0.90	Ngs (Ntg, GoN, FPMP)
12801	CNR1	0.88	SyT, Ngs (GoN, DCNS, DoN)
53404	ATOH7	0.85	Ngs (GoN, DoN)
63993	SLC5A7	0.84	SyT
20418	SHC3	0.80	SyT, Ngs (DCNS, DoN)
18213	NTRK3	0.80	Ngs (Ntg, GoN, FPMP, DoN)
15567	SLC6A4	0.80	Ngs (DCNS, DoN)
20665	SOX10	0.80	Ngs (DCNS)
19049	PPP1R1B	0.78	Ngs (DoN)
12570	CDK5R2	0.77	Ngs (GoN, DCNS, DoN)
12161	BMP6	0.75	Ngs (GoN, DoN)
20562	SLIT1	0.74	Ngs (Ntg, GoN, FPMP, DCNS)
21834	THRB	0.71	Ngs (DCNS)
22422	WNT7B	0.70	Ngs (Ntg, FPMP)
192167	NLGN1	0.68	SyT, Ngs (DoN)
230777	HCRTR1	0.67	SyT
16590	KIT	0.66	Ngs (GoN)
12286	CACNA1A	0.66	Ngs (Ntg, FPMP, DCNS, DoN)
18081	NINJ1	0.66	Ngs (GoN)
22421	WNT7A	0.65	Ngs (Ntg, FPMP, DoN)
23859	DLG2	0.64	SyT
17172	ASCL1	0.64	Ngs (Ntg, FPMP, DCNS, DoN)
13616	EDN3	0.63	Ngs (DoN)
12005	AXIN1	0.62	Ngs (FPMP)
70357	KCNIP1	0.62	SyT
13492	DRD5	0.59	SyT
13796	EMX1	0.56	Ngs (DCNS, DoN)
11682	ALK	0.54	Ngs (GoN)
18125	NOS1	0.54	Ngs (Ntg, FPMP, DoN)
319924	APBA1	0.51	SyT
22412	WNT9B	0.48	Ngs (DoN)
11555	ADRB2	0.47	SyT, Ngs (GoN, DCNS)
22248	UNC119	0.46	SyT
12048	BCL2L1	0.44	Ngs (DCNS, DoN)
14586	GFRA2	0.39	Ngs (Ntg, GoN, FPMP)
67903	GIPC1	0.37	SyT
16825	LDB1	0.37	Ngs (DCNS)
19697	RELA	0.34	Ngs (GoN)
14804	GRID2	0.32	SyT
18753	PRKCD	0.31	Ngs (GoN)
12799	CNP	0.30	SyT, Ngs (Ntg, FPMP)
17528	MPZ	0.29	SyT, Ngs (Ntg, GoN, FPMP)
13871	ERCC2	0.28	Ngs (DCNS, DoN)
18019	NFATC2	0.27	Ngs (GoN)
171166	MCOLN3	−0.22	Ngs (DoN)
241568	LRRC4C	−0.26	Ngs (Ntg, FPMP)
242316	GDF6	−0.28	Ngs (FpMP, DoN)
18126	NOS2	−0.32	Ngs (DCNS)
26419	MAPK8	−0.35	Ngs (Ntg, FPMP, GoN, DCNS, DoN)
110886	GABRA5	−0.37	SyT
15234	HGF	−0.38	Ngs (Ntg, FPMP, GoN, DCNS, DoN)
16870	LHX2	−0.39	Ngs (DCNS, DoN)
16176	IL1B	−0.44	SyT, Ngs (Ntg, FPMP, GoN, DCNS, DoN)
19877	ROCK1	−0.61	Ngs (GoN, FPMP)
13654	EGR2	−0.69	Ngs (DCNS)
216858	KCTD11	−0.73	Ngs (DoN)
14173	FGF2	−0.87	SyT, Ngs (Ntg, FPMP, GoN, DCNS, DoN)
21926	TNF	−1.21	SyT, Ngs (Ntg, GoN, FPMP, DCNS)

The neurogenesis biofunction is subsequently subcategorized into differentiation of neurons (DoN), growth of neurites (GoN), differentiation of nervous system (DNS), formation of plasma membrane projections (FPMP), and neuritogenesis (Ntg). The genes are identified by the Entrez IDs and gene symbols. The log ratio indicates the log_1.5_ transformed of the average transcriptomic expressions from FD-/SD-fed mice.

DoN and GoN shared five transcripts: *BMP6*, *CDK5R2*, *CNR1*, *NTRK3*, and *ATOH7*; all of them were elevated by FD. In addition, there were four transcripts shared by all five sub-categories; *NGFR* was elevated, and *FGF2*, *HGF* and *MAPK8* were all suppressed by FD.

Synaptic transmission (SyT) (Figure 3B, Table 5) was a highly significant sub-family of nervous system development and function (34 molecules, 23.3% of parent function; *p* = 4.9×10^−7^) sharing 10 common genes with Ngs (*CNR1*, *SHC3*, *NLGN1*, *ADRB2*, *CACNA1A*, *CNP*, and *MPZ* were elevated and *TNF*, *FGF2*, and *IL1B* were suppressed by FD).

DoN, GoN and SyT enlisted 80%, 79% and 88% elevated genes, respectively. In DoN, the proteins associated with these transcripts are almost equally distributed among the extracellular space (8 genes), plasma membrane (7 genes), cytoplasm (6 genes) and nucleus (8 genes). On the other hand, proteins encoded by 48% and 71% of the transcripts associated with GoN and SyT, respectively, are localized in the plasma membrane.

FD-elevated transcripts encoding proteins are associated with a wide ensemble of transmembrane receptors and transportation functions (*SOX10*, *CHRNB3*, *GFRA2*, *NGFR*, *APBA1*, *GJB1*, *SLC18A3*, *SLC5A7*, *SLC6A4* and *SLC8A3*) and membrane-based G-protein coupled reception (*ADBR2*, *CCR5*, *CHRM3*, *CNR1*, *DRD5*, *GPR6*, *GRM2*, *GRM7*, *HCRTR1* and *NTSR1*). FD further increased the expression of most of the genes regulating transcription, such as *ASCL1*, *EMX1*, *HEYL*, *LDB1*, *LZTS1*, *NFATC2*, *NKX2-2* and *RELA*, excluding two, *EGR2* and *LHX2*. Alternatively, FD suppressed the transcripts linked to the growth factors (*FGF2*, *HGF* and *GDF6*) and the cytokines (*IL1B* and *TNF*). The transcripts regulating the protein kinases displayed a mixed expression profile; *ALK*, *DLG2*, *KIT*, *NTRK3* and *PRKCD* were elevated, but *MAPK8* and *ROCK1* were suppressed by FD.

#### 3.1.iii. Apoptosis and immune response

Apoptosis and immune response were the two most enriched sub-categories of “cell death” and “inflammatory response” respectively, which explained 48% and 95% of the transcripts enlisted by the respective parent terms. Together, they shared 34 genes; 23 of these were suppressed (Table S5).Most of the proteins encoded by these transcripts associated with apoptosis (30%) and immune response (35%) are localized in the plasma membrane.

The pool of cytokine associated transcripts (*CSF2*, *CXCL11*, *IK*, *IL10*, *IL19*, *IL1B*, *PRL*, *TNF* and *TNFSF14*) was suppressed in FD-fed mice excluding *IL25*elevated. Likewise, a large panel of CD markers excluding *CD4* was suppressed by FD. The list included *CD2*, *CD3G*, *CD80*, *CD28* and *CD7*, which are associated with T-cells; *CD5* and *CD48* are associated with B-cells; *CD244* is associated with both NK-cells and T-cells; and *CD226* is associated with platelets as well as T-cells. Alternatively, all the transcripts associated with transportation (*GJA4*, *LBP* and *SFTPA1*) were elevated. Among the growth factors, *BMP6* and *PGF* transcripts were elevated; whereas *FGF2* and *HGF* were suppressed. All of the kinase-transcripts encoding protein localized in the plasma membrane were elevated (*ALK*, *KIT* and *NTRK3*), while the rest (*CHEK1*, *MAPK8*, *PDPK1*, *PRKX* and *ROCK1*) were suppressed by FD.

### 3.2 Validation of gene expression by the real-time PCR analysis

**Figure 4:**
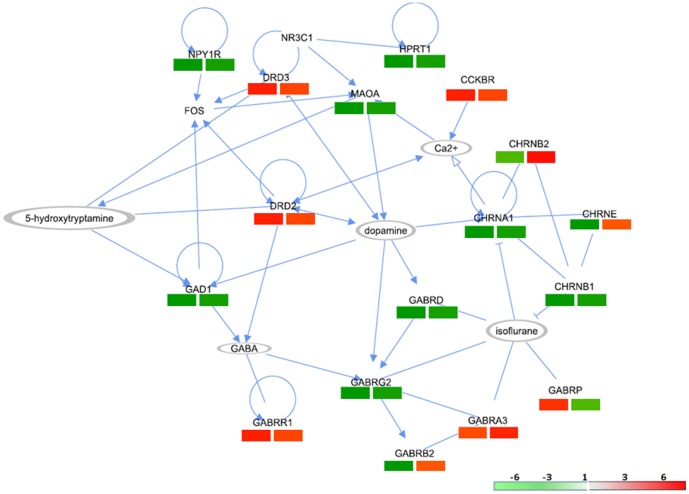
Regulatory networks showing clusters of differentially-regulated genes predicted by mRNA microarray and validated by 96-well qPCR assay. The nodes represent the genes and the solid lines depict the interactions between two molecules. Red- and green-colored panels show significantly increased and decreased expression in the FD-fed mice compared to the SD-fed mice (log_1.5_ (FD-/SD-fed mice). The right and left panels represent the mRNA microarray and qPCR output, respectively. The molecules without color are not mined by the present analysis; they are used to build the networks. These molecules constitute the major hubs of this network, but are not identified in the data shown here.

The elevated expressions of *DRD3*, *GABRR1*, *CCKBR* and *GABRA3* encoding proteins localized in the membrane were validated by PCR array (Figure 4). The suppressed genes validated by qPCR included *MAOA*, *GAD1*, *HPRT1*, *NPY1R*, *CHRNA1*, *CHRNB1*, *GABRD* and *GABRG2*. The assay could not confirm the expressions of *CHRNB2*, *CHRNE*, *GABRP* and *GABRB2*.

**Figure 5:**
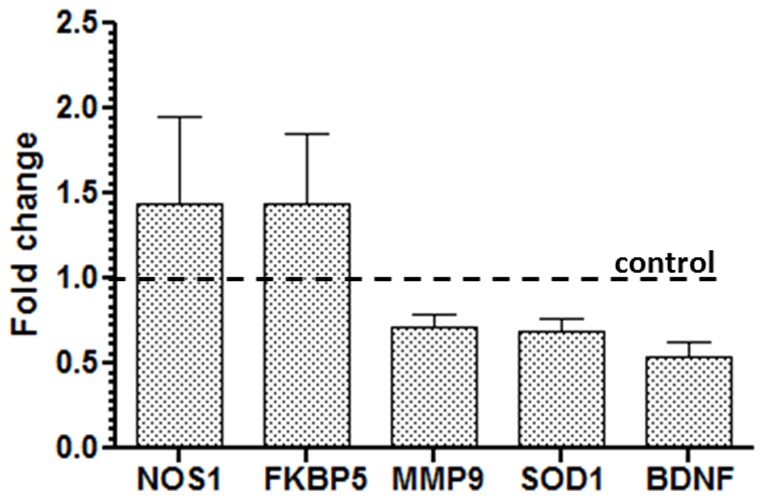
Genomic regulation of five genes associated with physiological stress (*NOS1*, *FKBP5*, *MMP9*, *SOD1* and *BDNF*) validated by real-time PCR assay. The bar graph shows the genomic fold changes (FD-fed/SD-fed mice) with ± SEM. The horizontal line represents the control line depicting the fold change due to FD consumption.

By using the low throughput qPCR assay (Figure 5), we confirmed that *FKBP5* and *NOS-1* were elevated by FD while *BDNF*, *MMP9* and *SOD1* were suppressed.

Altogether, the qPCR assay validated 76% of the genomic expressions tested herein.

## Discussion

A multitude of *in vitro* results suggests potential benefits of n-3 PUFA enriched diets typically supplemented by fish oil, but translating the knowledge to a viable therapeutic model remains challenging [10], [12], [13], [52]–[54]. There is a necessity for a more systematic comprehension of the etiologic relationships between the fish oil and their reported effects on various illnesses.

In light of the several studies evaluating the range of the compositions of n-3/n-6 PUFA [21]–[25], [55], [56], our study focused on investigating one particular dietary supplement, namely the fish oil enriched with high n-3 PUFAs, EPA and DHA in particular. This high fat composition with a consequence of increased body mass is certainly a concern [57] that should be addressed by a more comprehensive investigation in the future. Furthermore, we investigated the effects on the hemibrains only; dietary effects on various brain sub-regions were not assessed as reported by others [40]. Present study also did not attempt to quantify the shift of fatty acid levels in the brain as a result of consuming a high fat diet that investigated in past [58]. The genomic profiling of the hemibarns could be further biased by the collection of the small midline structures such as thalamic and hypothalamic nuclei.

It is important to note that the present study solicited the molecular impacts of the fish oil, not the consequences of n-3 PUFA. The study was designed to critically comprehend the performances of the fish oil including its benefits and adverse effects. Therefore FD delivered more fish oil derived fatty acids (high n-3 PUFA; but sub-optimal n-6 PUFA) than the controls following many such precedents [42], [59]–[63]. SD with nearly equivalent caloric contribution had unadjusted fat supplements like many other studies [42], [59]–[63]. Adjusting the fatty acid presentation in the diet potentially derived from other sources, such as soybeans might alter the molecular makeups of the rodents risking confounding the outcome.

The FD contained 5.2% of n-3 PUFA and 0.65% of n-6 PUFA, presenting a ratio of 8∶1 of n-3 PUFA: n-6 PUFA; the SD contained 0.2% and 1.22% of n-3 and n-6 PUFA, respectively, with a ratio of 1∶6. By mass, FD consisted of 16% fish oil, and the total fat composition in FD was 1.5 fold higher than SD. FD contained twenty times more n-3 PUFA and 50% less n-6 PUFA as present in SD. Furthermore, FD contained a 1.5∶1 ratio of the EPA: DHA and a 7∶1 ratio of EPA: AA, which was in agreement with the literature showing health benefits [23]–[26], [56], [64]. By weight, other major diet components including protein, carbohydrate, vitamins and minerals were similar in FD and SD, and the gross energy levels were also equivalent (Table 1).

FD and SD were introduced to randomly chosen mouse groups at three weeks of age. The animals consumed their respective diets for another five months, which is equivalent to their late adolescence. Subsequently, the mice were euthanized to collect the brain samples for transcriptomic analysis.

The two mouse populations gained body weight differently. FD mice gained 18% more weight with a marginal significance (*p* = 0.06) in accordance with a previous study [65]. This could possibly be a negative effect of the high lipid diet [57]; further optimization of the dietary components is required to control the weight gain and consequent health issues. It is, however, beyond the scope of the present study to discuss the potential markers associated with obesity, such as the genes associated with fatty acids (*SLC27a4*, *FADS2*, *FA2H*, and *FADS6*; all up-regulated by FD) and insulin treatment (*INSIG1* up-regulated and *IRS1*, *IGFBP2*; all down-regulated by FD) (Table S2).

### 4.1. Fish oil mediated augmentation of the brain and nervous system

The functional consequences of synaptic plasticity include the power of retention and retrieval of memory; and shifting of learning and behavioral paradigms [66], [67]. Environments combined with diets can critically modify the synaptic networks that involve formations, differentiation, repositioning, and elimination of synapse.

The NMDA receptors (NMDARs) are the critical regulators of synaptic plasticity and particularly of the long term consequences such as memory retention [43], [66], [68], [69]. Src-mediated direct phosphorylation of NMDARs initiates the presynaptic influx of Ca^+2^ [68]. FD apparently activated this presynaptic pathway by elevating the key transcriptomic members, including *nSHC* (also known as *SHC3*), *NMDAR2A* (also known as *GRIN2A*) and *CACNA1A*. Promoting the excitatory against the inhibitory synaptic transmissions [70], *CACNA1A* contributes to a number of neurological disorders [71]. . Unlike *CACNA1A*, which closely regulates the nervous system, *CACNA1S* suppressed by FD is associated with photoreception and thermoregulation [72], [73].

NMDAR-mediated influx of Ca^+2^ contributes to the long term potentiation, thereby controlling the behavioral learning, fear response and extinction [66], [69], [74]. Related observation of elevated *mGluR2* (*GRM2*) in FD-fed mice was rather counterintuitive. *mGluR2* in association with moderate Ca^+2^ influx is linked to long term depression [75]. Addressing the paradox, literatures suggest that the temporal pattern of Ca^+2^ deposits may shift the consequence from depression to potentiation, and differential buffering of intracellular Ca^+2^ may also shift the balance [68]. Although we could not rule out the possibility of FD-induced long term depression, our results described hereafter increasingly favored the possibility that FD preferentially promoted long term potentiation.

Supporting the hypothesis, FD enhanced glutamatergic activity ( elevated *DRD-2/-3/-5*; *GRM-2/-7*; and *GRI-D2/-N2a* in the FD-fed mice) driving the calcium and dopamine messengers to phosphorylate the protein phosphatase 1 regulatory subunit 1B [69]. Elevated *PPP1R1B*, also observed by administering DHA-enriched diet [40] is critically related to the emotional learning associated with long term potentiation [76]. In addition, FD elevated a group of transcriptional members associated with choline influx, such as *CHRM3*, *CHRNB3* and *SLC5A*, which typically promote long term potentiation [77]. FD induced elevation of *PDPK1* and *AKT* (alternate name: *PKB*), two integral downstream members of PI3K pathways, is likely to cause spine enlargement and stability, thus promoting long term potentiation maintenance [78].

Together, the accumulated evidence from this study increasingly linked FD to long term potentiation, as opposed to long term depression.

Emerging knowledge elucidating the complexity of BDNF activity regulation begins to dissociate BDNF from the robust synapse as suggested in past [79]–[81]. Researchers have found that the TRKB-independent pro-BDNF signaling pathway is the causative factor of compromised synapse [82], and potentially eliciting depression [83]. In comparison to the remote PTSD patients, recent PTSD patients have been diagnosed with higher levels of BDNF that likely contribute to the biased consolidation of fear memory [84]. Evidently, the memory consolidation process is exon-specific. Prolonged stress applied on the rodents significantly elevated BDNF mRNA levels in exon I (35%), IV (20%) and IX (16%), and substantially elevated mRNA of exon II (20%) [85]. In this context, our microarray data predicted suppressed exon II of BDNF (NM_007540, vendor-delivered information, Table S1) in FD-fed mice, and the qPCR assay (Figure 5) validated the outcome.

Conversion of pro-BDNF to mature BDNF is mediated by MMP-9, [86], [87], which was transcriptionally suppressed by FD. Supporting evidence reported the n-3 PUFA-induced inhibition of MMP-9 in brain cells challenged by an endotoxic assault *in vitro* [87].

Hence, we postulate that FD promotes axonal growth by steering a BDNF-independent network critically regulated by Src to activate PI3K-AKT-PKC downstream. In fact, the independent actions of Src and BDNF have been reported in the past as a response to a dietary imbalance caused by zinc deficiency [88].

### 4.2 Fish oil mediated neuroprotection

Consistent with a past study [40], here we also reported a host of elevated genes in FD-fed mice associated with the myelin accumulation and preservation, including *MOG*, *MPZ* and *MAG*. Demyelinated lesions in neuronal cells are early indicators of inflammation that lead to the rapid destruction of the myelin sheath, causing increased vulnerability of the axons [89]. In fact, selective loss of MOG was identified as a serologic marker of inflammatory brain disease [90].

Pro-inflammatory cytokines such as *TNF-a*, *IL-1B*, *IL-10* and *TNFSF1* showed decreased transcriptomic expression, while the transcription of interferon gamma antagonist (*IK*) was elevated by FD in accordance to many past studies [91]–[94]. A study conducted on healthy young human adults identified the lower levels of n-3 PUFA (or a higher n-6: n-3 ratio) in serum as the primary cause of the larger influx of IFN-γ and TNF-α [6], [95]. Of particular note, we observed elevated *RELA* in FD mice; a rather counterintuitive observation, particularly in light of the suppressed expression of related genes like *TNF*, and the relevant past studies [96]. One possible reason could be the near obesity condition of the FD-fed mice; a high fat diet allegedly increases NF-κB activities in rodents [97].

The immunological implications of fish oil were further supported by the comprehensive FD induced suppression of CD markers associated with T-cells, B-cells, NK-cells and platelets. Emerging knowledge suggests that there are inhibitory effects of n-3 PUFA on CD cells, the potential markers of depression [98].

Down-regulation of apoptotic pathways and promotion of cell survival are known implications of n-3 PUFA enriched diets. Elevated *AKT* and *BCL21A* in FD-fed mice are the established markers of cell survival [99] [100]. The suppressed genes encoding the caspase family (*Casp -1/-4/-7/-14*) may suggested the diet's potential protective roles against inflammation by regulating the caspase family members [101]–[103].

### 4.3 Potential therapeutic efficacy of FD treating neurodegenarative disorders

The motivation of testing fish oil as the dietary supplements could be derived from the ongoing clinical trial activities evaluating the agonists of GABRG-3, GABRA-4, DRD-5, and THRB (all were elevated by FD), and the antagonists of GABRA-5 (suppressed by FD) for treating depression (www.clinicaltrials.gov). On the other hand, the antagonists targeting GABRG-3, GABRG-4, DRD5, ADR -A1D/–B2, CHRM-3, and SLC6A4 (all elevated by FD) are under investigation for treating bipolar disorder and schizophrenia.

In contrast, FD-regulated transcripts could be relevant in eliciting the risk of the schizophrenia, bipolar disorder, and suicidal inclination. FD, for instance activated *ADR-A1D/-B2* and *SLC6A4*; and the activated adrenergic and serotoninergic pathways are linked to augment the suicidal psychopathology [104]. In addition, several of the GABA family members (*GABRG-3*, *GABRG-4*) and key dopamine receptors (*DRD5*) were also elevated by FD. The activities of GABA families and dopaminergic hyperactivity are positively correlated with the prevalence of bipolar disorder and schizophrenia [105] [106]. Contrastingly, dopaminergic hypoactivity has been recognized as a possible precursor of depression [107]. This observation emphasizes the fact that the efficacy of fish oil potentially depends on the disease pathophysiology.

Affective disorder is typically triggered by the decreased activities of antioxidants such as glutathione and the depletion of serological markers such as BDNF and somatostatin [108]. Our report showed FD-induced elevation of a number of transcripts related to transmembrane glutathione transfer (*Gstm -4/-6*, and *Gstt-1*) (Table S2) accompanied by *CORT* encoding cortistatin, and thereby likely activated of the somatostatin receptors. Among other neurodegenerative diseases, the reported impacts of FD on AD and PD were in accordance with a multitude of existing evidence [9], [10], [12], [31]. FD suppressed *APBA1*, a key gene encoding the amyloid beta (A4) precursor protein-binding family; thereby potentially suppressed the amyloidal toxicity, a vital precursor of AD progression [109]. Possible activation of the dopaminergic cascade, evident from the elevated receptors (*DRD-2/-3/-5*), indicated a stronger efficacy of FD in treating PD.

In addition, FD altered a collection of transcriptomic expression linked to AD and PD onset [110], [111], such as *NOS-2*, *SOD1* and caspases (*CASP-1/-4/-7*). Accumulated evidences underscore the importance of the beta-adrenergic receptors and MAPK signaling towards AD pathology [112]; members of these respective networks (*ADR-A1D/-B2* and *MAPK-8*) were altered by FD.

FD mice showed a comprehensive elevation of the transcripts associated with voltage-gated potassium channels (*KCNMB3*, *KCNQ5*, *KCN1P1*, *KCNC4*, *KCNC3*, *KCNA5*, *KCNJ6*, *KCTD7* and *KCNQ2*) and cholinergic modulation (*CHRM3 and CHRNB3*), which are associated with memory consolidation and stress management [113]–[115]. Among another psychological illnesses, FD showed positive implications in PTSD, regulating some of the potential therapeutic markers like *BDNF*, *FKBP5* and *SLC6A4* [116] (Table 2). In addition, Table 2 shows a list of psychological debilities associated with a panel of transcripts investigated by qPCR.

## Conclusions

The biomechanisms exhibited by the fish oil that facilitate neurotrophic regulation are still elusive. The knowledge gap limits our predictive aptitude in realistically estimating the efficacy of this dietary supplement. To address this challenge, we customized a diet enriched of 16% fish oil by weight, the optimum fraction of oil added to the rodent food without altering its texture from the standard lab diet. Two randomly chosen groups of C57BL/6J mice consumed one of the two diets from weaning age until their young adulthood. We sacrificed five month old mice and investigated their hemibrains using a genome-wide transcriptomic platform.

Functional analyses of the transcriptomic data focused on the areas of the *neurogenesis, neuroviability and neurodegenerative disorders.* The GOI were clustered into four most significant functions: “Neurological disease”, “Nervous system development and function”, “Immune response” and “Apoptosis”. Together these functions encompassed 247 transcripts (∼20%) identified from 1,142 genes displaying the most differential expression between the FD- and SD-fed mice. Approximately 40% of the proteins associated with these genes are located in the plasma membrane, suggesting a biased impact of the fish oil on the membrane-associated activities. Leveraging from the mouse brain atlas (mouse.brain-map.org) we found several of these genes found enriching the brain regions (Table S4) such as olfactory bulb, cerebellar cortex, thalamus and amygdala typically associated with the motor activities, information relay and neuropsychology.

In FD-fed mice, a Src-mediated calcium-controlled network was suggested to reinforce synapse that possibly operated in a BDNF-MMP-9 independent pathway. FD suppressed the transcription of BDNF exons II, which is known to express in response to stress [85]. Activated PI3K-AKT-PKC cascades operated in concert. We further acknowledged a risk of spurring depression through FD-mediated activation of mGluR2/Ca^+2^ [75]. However, the FD-induced long term potentiation was deemed as more plausible endpoint in cognition of the additional evidences, including the stimulation of NMDAR and downstream glutaminergic/dopaminergic/cholinergic networks [69], [77] and the elevated transcription of WNT family genes [117], *PPP1R1B* [76] and *NEUROD6* [118]. The risk of Ca^+2^ -induced biased recurrence of excitatory synaptic transmission was possibly mitigated by the FD-induced *NLGN1* that typically helps in restoring the balance between the excitatory and inhibitory synapses [119].

FD-induced attenuation of oxidative stress and reduction of amyloidal burden coupled with suppressed inflammatory and apoptosis networks affirmed the potential efficacy of fish oil in treating a range of neurodegenerative debilities such as affective, Alzheimer's and Parkinson's disorders. In contrast, the FD-stimulated neuromodulators like dopamine, choline, GABA and glutamate may diminish the efficacy of the fish oil in treating bipolar disorder, schizophrenia and suicidal inclination, which has been proposed to be triggered by the hyperactivity of the same pool of molecular messengers [105], [106]. Taken together, our results suggested a potential susceptibility of the fish oil's efficacy on the disease pathology and highlighted the need for strategic repositioning of the fish oil as a therapeutic adjuvant. Supporting our results a number of past in vitro rodent models [41] [37] [35], [36], [38], [39] reported n-3 PUFA benefits in the restoration of myelin sheath, activation of dopaminergic networks with downstream elevation of *PPP1R1B*, activation of synaptic plasticity and comprehensive attenuation of inflammasome networks.

In conclusion, fish oil could provide therapeutic benefits for a number of neurodegenerative diseases, while there is a valid concern about its therapeutic efficacy in treating certain other illnesses. The comprehensive investigation to confirm these findings are exigent and the effectiveness of this fish oil diet should be validated by introducing this diet type to specific disease models.

## Supporting Information

Figure S1 **The volcano plot of the GOI depicting the expression distribution in FD and SD fed murine hemibrains.** Here the x-axis depicts the log_2_(fold change) and y-axis depicts -log_10_(*p*-value), where p-value is calculated by FDR algorithm. The red and green dots represent the up- and down-regulated transcripts, respectively.
(TIF)

Figure S2 **Regulatory networks showing an additional three clusters of differentially-regulated genes involved in “neurogenesis”.** The nodes represent the genes and the solid lines depict the interactions between two molecules. Red- and green-colored genes show significantly increased and decreased expression (log_1.5_ (FD-/SD-fed mice) in the FD-fed mice compared to the SD-fed mice (a color scale is at the bottom right corner). The genes are clustered based on their proteins' cellular locations in the nucleus, cytoplasm, plasma membrane, and extracellular space. Three subgroups are “differentiation of nervous system” (left panel), “formation of plasma membrane projections” (middle panel) and “neuritogenesis” (right panel). They enlist 29, 26, and 23 genes with *p* values of 7.6×10^−23^, 4.71×10^−19^ and 8.02×10^−18^, respectively.
(TIF)

Table S1 **List of primers and probes used in qPCR assay.**
(DOCX)

Table S2 **List of 1,142 genes of interest (GOI).** The genes are identified by the Gene Name and gene symbol. The log ratio indicates the log_1.5_ transformed of the average transcriptomic expressions from FD-/SD-fed mice.
(DOCX)

Table S3 **Complete list of biofunction categories enriched by 1,142 genes.** For individual functional categories, corresponding range of *p* values and the number of enriching genomic members are reported.
(DOCX)

Table S4 **The brain regions enriched by the genes mined by present study relevant to “Neurological disease”, “Nervous system development and function”, “Inflammatory response” and “Cell death”.**
(DOCX)

Table S5 **The genomic candidates associated with apoptosis (Apop) and immune response (ImRes).** The genes are identified by the Entrez IDs and gene symbols. The log ratio indicates the log_1.5_ transformed of the average transcriptomic expressions from FD-/SD-fed mice.
(DOCX)

## References

[1] Deckelbaum RJ, Torrejon C (2012) The omega-3 fatty acid nutritional landscape: health benefits and sources. J Nutr 142: 587S–591S. 22323763 10.3945/jn.111.148080 PMC3278270

[2] Seo T, Blaner WS, Deckelbaum RJ (2005) Omega-3 fatty acids: molecular approaches to optimal biological outcomes. Curr Opin Lipidol 16: 11–18. 15650558 10.1097/00041433-200502000-00004

[3] Amminger GP, Schafer MR, Papageorgiou K, Klier CM, Cotton SM, et al (2010) Long-chain omega-3 fatty acids for indicated prevention of psychotic disorders: a randomized, placebo-controlled trial. Arch Gen Psychiatry 67: 146–154. 20124114 10.1001/archgenpsychiatry.2009.192

[4] Kiecolt-Glaser JK, Belury MA, Andridge R, Malarkey WB, Hwang BS, et al (2012) Omega-3 supplementation lowers inflammation in healthy middle-aged and older adults: a randomized controlled trial. Brain Behav Immun 26: 988–995. 22640930 10.1016/j.bbi.2012.05.011 PMC3398219

[5] Im DS (2012) Omega-3 fatty acids in anti-inflammation (pro-resolution) and GPCRs. Prog Lipid Res 51: 232–237. 22542696 10.1016/j.plipres.2012.02.003

[6] Kiecolt-Glaser JK, Belury MA, Andridge R, Malarkey WB, Glaser R (2011) Omega-3 supplementation lowers inflammation and anxiety in medical students: a randomized controlled trial. Brain Behav Immun 25: 1725–1734. 21784145 10.1016/j.bbi.2011.07.229 PMC3191260

[7] Ho L, van Leeuwen R, Witteman JC, van Duijn CM, Uitterlinden AG, et al (2011) Reducing the genetic risk of age-related macular degeneration with dietary antioxidants, zinc, and omega-3 fatty acids: the Rotterdam study. Arch Ophthalmol 129: 758–766. 21670343 10.1001/archophthalmol.2011.141

[8] Tuo J, Ross RJ, Herzlich AA, Shen D, Ding X, et al (2009) A high omega-3 fatty acid diet reduces retinal lesions in a murine model of macular degeneration. Am J Pathol 175: 799–807. 19608872 10.2353/ajpath.2009.090089 PMC2716974

[9] Hooijmans CR, Pasker-de Jong PC, de Vries RB, Ritskes-Hoitinga M (2012) The effects of long-term omega-3 fatty acid supplementation on cognition and Alzheimer's pathology in animal models of Alzheimer's disease: a systematic review and meta-analysis. J Alzheimers Dis 28: 191–209. 22002791 10.3233/JAD-2011-111217

[10] Jicha GA, Markesbery WR (2010) Omega-3 fatty acids: potential role in the management of early Alzheimer's disease. Clin Interv Aging 5: 45–61. 20396634 10.2147/cia.s5231 PMC2854051

[11] Bousquet M, Calon F, Cicchetti F (2011) Impact of omega-3 fatty acids in Parkinson's disease. Ageing Res Rev 10: 453–463. 21414422 10.1016/j.arr.2011.03.001

[12] da Silva TM, Munhoz RP, Alvarez C, Naliwaiko K, Kiss A, et al (2008) Depression in Parkinson's disease: a double-blind, randomized, placebo-controlled pilot study of omega-3 fatty-acid supplementation. J Affect Disord 111: 351–359. 18485485 10.1016/j.jad.2008.03.008

[13] Lesperance F, Frasure-Smith N, St-Andre E, Turecki G, Lesperance P, et al (2011) The efficacy of omega-3 supplementation for major depression: a randomized controlled trial. J Clin Psychiatry 72: 1054–1062. 20584525 10.4088/JCP.10m05966blu

[14] Logan AC (2004) Omega-3 fatty acids and major depression: a primer for the mental health professional. Lipids Health Dis 3: 25. 15535884 10.1186/1476-511X-3-25 PMC533861

[15] Ross BM (2009) Omega-3 polyunsaturated fatty acids and anxiety disorders. Prostaglandins Leukot Essent Fatty Acids 81: 309–312. 19906519 10.1016/j.plefa.2009.10.004

[16] Brenna JT, Diau GY (2007) The influence of dietary docosahexaenoic acid and arachidonic acid on central nervous system polyunsaturated fatty acid composition. Prostaglandins Leukot Essent Fatty Acids 77: 247–250. 18023566 10.1016/j.plefa.2007.10.016 PMC2174532

[17] Cole GM, Ma QL, Frautschy SA (2009) Omega-3 fatty acids and dementia. Prostaglandins Leukot Essent Fatty Acids 81: 213–221. 19523795 10.1016/j.plefa.2009.05.015 PMC4019002

[18] Duffy EM, Meenagh GK, McMillan SA, Strain JJ, Hannigan BM, et al (2004) The clinical effect of dietary supplementation with omega-3 fish oils and/or copper in systemic lupus erythematosus. J Rheumatol 31: 1551–1556. 15290734

[19] Matsuoka Y (2011) Clearance of fear memory from the hippocampus through neurogenesis by omega-3 fatty acids: a novel preventive strategy for posttraumatic stress disorder? Biopsychosoc Med 5: 3. 21303552 10.1186/1751-0759-5-3 PMC3045887

[20] Ross BM, Seguin J, Sieswerda LE (2007) Omega-3 fatty acids as treatments for mental illness: which disorder and which fatty acid? Lipids Health Dis 6: 21. 17877810 10.1186/1476-511X-6-21 PMC2071911

[21] Martins JG (2009) EPA but not DHA appears to be responsible for the efficacy of omega-3 long chain polyunsaturated fatty acid supplementation in depression: evidence from a meta-analysis of randomized controlled trials. J Am Coll Nutr 28: 525–542. 20439549 10.1080/07315724.2009.10719785

[22] Freeman MP, Hibbeln JR, Wisner KL, Brumbach BH, Watchman M, et al (2006) Randomized dose-ranging pilot trial of omega-3 fatty acids for postpartum depression. Acta Psychiatr Scand 113: 31–35. 16390366 10.1111/j.1600-0447.2005.00660.x

[23] Adams PB, Lawson S, Sanigorski A, Sinclair AJ (1996) Arachidonic acid to eicosapentaenoic acid ratio in blood correlates positively with clinical symptoms of depression. Lipids 31 Suppl: S157–161. 8729112 10.1007/BF02637069

[24] Conklin SM, Manuck SB, Yao JK, Flory JD, Hibbeln JR, et al (2007) High omega-6 and low omega-3 fatty acids are associated with depressive symptoms and neuroticism. Psychosom Med 69: 932–934. 17991818 10.1097/PSY.0b013e31815aaa42

[25] Lucas M, Mirzaei F, O'Reilly EJ, Pan A, Willett WC, et al (2011) Dietary intake of n-3 and n-6 fatty acids and the risk of clinical depression in women: a 10-y prospective follow-up study. Am J Clin Nutr 93: 1337–1343. 21471279 10.3945/ajcn.111.011817 PMC3095504

[26] Simopoulos AP (2011) Evolutionary aspects of diet: the omega-6/omega-3 ratio and the brain. Mol Neurobiol 44: 203–215. 21279554 10.1007/s12035-010-8162-0

[27] Bourre JM, Bonneil M, Dumont O, Piciotti M, Calaf R, et al (1990) Effect of increasing amounts of dietary fish oil on brain and liver fatty composition. Biochim Biophys Acta 1043: 149–152. 2317525 10.1016/0005-2760(90)90288-9

[28] Bourre JM, Bonneil M, Dumont O, Piciotti M, Nalbone G, et al (1988) High dietary fish oil alters the brain polyunsaturated fatty acid composition. Biochim Biophys Acta 960: 458–461. 3382685 10.1016/0005-2760(88)90055-0

[29] Bhatia HS, Agrawal R, Sharma S, Huo YX, Ying Z, et al (2011) Omega-3 fatty acid deficiency during brain maturation reduces neuronal and behavioral plasticity in adulthood. PLoS One 6: e28451. 22163304 10.1371/journal.pone.0028451 PMC3233581

[30] Chung WL, Chen JJ, Su HM (2008) Fish oil supplementation of control and (n-3) fatty acid-deficient male rats enhances reference and working memory performance and increases brain regional docosahexaenoic acid levels. J Nutr 138: 1165–1171. 18492851 10.1093/jn/138.6.1165

[31] Bousquet M, Gue K, Emond V, Julien P, Kang JX, et al (2011) Transgenic conversion of omega-6 into omega-3 fatty acids in a mouse model of Parkinson's disease. J Lipid Res 52: 263–271. 21115966 10.1194/jlr.M011692 PMC3023546

[32] Lim GP, Calon F, Morihara T, Yang F, Teter B, et al (2005) A diet enriched with the omega-3 fatty acid docosahexaenoic acid reduces amyloid burden in an aged Alzheimer mouse model. J Neurosci 25: 3032–3040. 15788759 10.1523/JNEUROSCI.4225-04.2005 PMC6725084

[33] Hennebelle M, Balasse L, Latour A, Champeil-Potokar G, Denis S, et al (2012) Influence of omega-3 fatty acid status on the way rats adapt to chronic restraint stress. PLoS One 7: e42142. 22860066 10.1371/journal.pone.0042142 PMC3408452

[34] Ferraz AC, Delattre AM, Almendra RG, Sonagli M, Borges C, et al (2011) Chronic omega-3 fatty acids supplementation promotes beneficial effects on anxiety, cognitive and depressive-like behaviors in rats subjected to a restraint stress protocol. Behav Brain Res 219: 116–122. 21192985 10.1016/j.bbr.2010.12.028

[35] Barcelo-Coblijn G, Hogyes E, Kitajka K, Puskas LG, Zvara A, et al (2003) Modification by docosahexaenoic acid of age-induced alterations in gene expression and molecular composition of rat brain phospholipids. Proc Natl Acad Sci U S A 100: 11321–11326. 13679584 10.1073/pnas.1734008100 PMC208755

[36] Harbeby E, Jouin M, Alessandri JM, Lallemand MS, Linard A, et al (2012) n-3 PUFA status affects expression of genes involved in neuroenergetics differently in the fronto-parietal cortex compared to the CA1 area of the hippocampus: effect of rest and neuronal activation in the rat. Prostaglandins Leukot Essent Fatty Acids 86: 211–220. 22579067 10.1016/j.plefa.2012.04.008

[37] Kitajka K, Puskas LG, Zvara A, Hackler L Jr, Barcelo-Coblijn G, et al (2002) The role of n-3 polyunsaturated fatty acids in brain: modulation of rat brain gene expression by dietary n-3 fatty acids. Proc Natl Acad Sci U S A 99: 2619–2624. 11880617 10.1073/pnas.042698699 PMC122397

[38] Kitajka K, Sinclair AJ, Weisinger RS, Weisinger HS, Mathai M, et al (2004) Effects of dietary omega-3 polyunsaturated fatty acids on brain gene expression. Proc Natl Acad Sci U S A 101: 10931–10936. 15263092 10.1073/pnas.0402342101 PMC503722

[39] Begg DP, Puskas LG, Kitajka K, Menesi D, Allen AM, et al (2012) Hypothalamic gene expression in omega-3 PUFA-deficient male rats before, and following, development of hypertension. Hypertens Res 35: 381–387. 22072108 10.1038/hr.2011.194

[40] Le-Niculescu H, Case NJ, Hulvershorn L, Patel SD, Bowker D, et al (2011) Convergent functional genomic studies of omega-3 fatty acids in stress reactivity, bipolar disorder and alcoholism. Transl Psychiatry 1: e4. 22832392 10.1038/tp.2011.1 PMC3309466

[41] Puskas LG, Bereczki E, Santha M, Vigh L, Csanadi G, et al (2004) Cholesterol and cholesterol plus DHA diet-induced gene expression and fatty acid changes in mouse eye and brain. Biochimie 86: 817–824. 15589691 10.1016/j.biochi.2004.10.004

[42] Yon MA, Mauger SL, Pickavance LC (2013) Relationships between dietary macronutrients and adult neurogenesis in the regulation of energy metabolism. Br J Nutr 109: 1573–1589. 23433235 10.1017/S000711451200579X

[43] Balcombe JP, Barnard ND, Sandusky C (2004) Laboratory routines cause animal stress. Contemp Top Lab Anim Sci 43: 42–51. 15669134

[44] Bourre JM, Piciotti M, Dumont O, Pascal G, Durand G (1990) Dietary linoleic acid and polyunsaturated fatty acids in rat brain and other organs. Minimal requirements of linoleic acid. Lipids 25: 465–472. 2120529 10.1007/BF02538090

[45] Cunnane SC, Keeling PW, Thompson RP, Crawford MA (1984) Linoleic acid and arachidonic acid metabolism in human peripheral blood leucocytes: comparison with the rat. Br J Nutr 51: 209–217. 6422980 10.1079/bjn19840025

[46] Hammamieh R, Chakraborty N, De Lima TC, Meyerhoff J, Gautam A, et al (2012) Murine model of repeated exposures to conspecific trained aggressors simulates features of post-traumatic stress disorder. Behav Brain Res 235: 55–66. 22824590 10.1016/j.bbr.2012.07.022

[47] Hammamieh R, Chakraborty N, Miller SA, Waddy E, Barmada M, et al (2007) Differential effects of omega-3 and omega-6 Fatty acids on gene expression in breast cancer cells. Breast Cancer Res Treat 101: 7–16. 16823509 10.1007/s10549-006-9269-x

[48] Berger JA, Hautaniemi S, Jarvinen AK, Edgren H, Mitra SK, et al (2004) Optimized LOWESS normalization parameter selection for DNA microarray data. BMC Bioinformatics 5: 194. 15588297 10.1186/1471-2105-5-194 PMC539276

[49] Al-Shahrour F, Minguez P, Tarraga J, Medina I, Alloza E, et al (2007) FatiGO +: a functional profiling tool for genomic data. Integration of functional annotation, regulatory motifs and interaction data with microarray experiments. Nucleic Acids Res 35: W91–96. 17478504 10.1093/nar/gkm260 PMC1933151

[50] Hammamieh R, Chakraborty N, Wang Y, Laing M, Liu Z, et al (2007) GeneCite: a stand-alone open source tool for high-throughput literature and pathway mining. OMICS 11: 143–151. 17594234 10.1089/omi.2007.4322

[51] Clark WF, Parbtani A (1994) Omega-3 fatty acid supplementation in clinical and experimental lupus nephritis. Am J Kidney Dis 23: 644–647. 8172205 10.1016/s0272-6386(12)70273-1

[52] Sublette ME, Ellis SP, Geant AL, Mann JJ (2011) Meta-analysis of the effects of eicosapentaenoic acid (EPA) in clinical trials in depression. J Clin Psychiatry 72: 1577–1584. 21939614 10.4088/JCP.10m06634 PMC3534764

[53] Bloch MH, Hannestad J (2012) Omega-3 fatty acids for the treatment of depression: systematic review and meta-analysis. Mol Psychiatry 17: 1272–1282. 21931319 10.1038/mp.2011.100 PMC3625950

[54] Sarris J, Mischoulon D, Schweitzer I (2012) Omega-3 for bipolar disorder: meta-analyses of use in mania and bipolar depression. J Clin Psychiatry 73: 81–86. 21903025 10.4088/JCP.10r06710

[55] Ross BM (2007) Omega-3 fatty acid deficiency in major depressive disorder is caused by the interaction between diet and a genetically determined abnormality in phospholipid metabolism. Med Hypotheses 68: 515–524. 17045757 10.1016/j.mehy.2006.07.054

[56] Simopoulos AP (2011) Importance of the omega-6/omega-3 balance in health and disease: evolutionary aspects of diet. World Rev Nutr Diet 102: 10–21. 21865815 10.1159/000327785

[57] von Au D, Brandle M, Rupp H, Jacob R (1988) Influence of a diet rich in fish oil on blood pressure, body weight and cardiac hypertrophy in spontaneously hypertensive rats. Eur J Appl Physiol Occup Physiol 58: 97–99. 2974415 10.1007/BF00636610

[58] Yu H, Bi Y, Ma W, He L, Yuan L, et al (2010) Long-term effects of high lipid and high energy diet on serum lipid, brain fatty acid composition, and memory and learning ability in mice. Int J Dev Neurosci 28: 271–276. 20015474 10.1016/j.ijdevneu.2009.12.001

[59] Grant WF, Gillingham MB, Batra AK, Fewkes NM, Comstock SM, et al (2011) Maternal high fat diet is associated with decreased plasma n-3 fatty acids and fetal hepatic apoptosis in nonhuman primates. PLoS One 6: e17261. 21364873 10.1371/journal.pone.0017261 PMC3045408

[60] Heerwagen MJ, Stewart MS, de la Houssaye BA, Janssen RC, Friedman JE (2013) Transgenic increase in N-3/n-6 Fatty Acid ratio reduces maternal obesity-associated inflammation and limits adverse developmental programming in mice. PLoS One 8: e67791. 23825686 10.1371/journal.pone.0067791 PMC3692451

[61] Lei X, Zhang W, Liu T, Xiao H, Liang W, et al (2013) Perinatal supplementation with omega-3 polyunsaturated Fatty acids improves sevoflurane-induced neurodegeneration and memory impairment in neonatal rats. PLoS One 8: e70645. 23967080 10.1371/journal.pone.0070645 PMC3742769

[62] Wakefield SL, Lane M, Schulz SJ, Hebart ML, Thompson JG, et al (2008) Maternal supply of omega-3 polyunsaturated fatty acids alter mechanisms involved in oocyte and early embryo development in the mouse. Am J Physiol Endocrinol Metab 294: E425–434. 18073322 10.1152/ajpendo.00409.2007

[63] Woodworth HL, McCaskey SJ, Duriancik DM, Clinthorne JF, Langohr IM, et al (2010) Dietary fish oil alters T lymphocyte cell populations and exacerbates disease in a mouse model of inflammatory colitis. Cancer Res 70: 7960–7969. 20798218 10.1158/0008-5472.CAN-10-1396

[64] Freeman MP (2006) Omega-3 fatty acids and perinatal depression: a review of the literature and recommendations for future research. Prostaglandins Leukot Essent Fatty Acids 75: 291–297. 16930971 10.1016/j.plefa.2006.07.007

[65] Guo J, Jou W, Gavrilova O, Hall KD (2009) Persistent diet-induced obesity in male C57BL/6 mice resulting from temporary obesigenic diets. PLoS One 4: e5370. 19401758 10.1371/journal.pone.0005370 PMC2670508

[66] Caroni P, Donato F, Muller D (2012) Structural plasticity upon learning: regulation and functions. Nat Rev Neurosci 13: 478–490. 22714019 10.1038/nrn3258

[67] Gogolla N, Galimberti I, Caroni P (2007) Structural plasticity of axon terminals in the adult. Curr Opin Neurobiol 17: 516–524. 17950593 10.1016/j.conb.2007.09.002

[68] Sheng M, Kim MJ (2002) Postsynaptic signaling and plasticity mechanisms. Science 298: 776–780. 12399578 10.1126/science.1075333

[69] Kotaleski JH, Blackwell KT (2010) Modelling the molecular mechanisms of synaptic plasticity using systems biology approaches. Nat Rev Neurosci 11: 239–251. 20300102 10.1038/nrn2807 PMC4831053

[70] Caddick SJ, Wang C, Fletcher CF, Jenkins NA, Copeland NG, et al (1999) Excitatory but not inhibitory synaptic transmission is reduced in lethargic (Cacnb4(lh)) and tottering (Cacna1atg) mouse thalami. J Neurophysiol 81: 2066–2074. 10322048 10.1152/jn.1999.81.5.2066

[71] Pietrobon D (2002) Calcium channels and channelopathies of the central nervous system. Mol Neurobiol 25: 31–50. 11890456 10.1385/MN:25:1:031

[72] Specht D, Wu SB, Turner P, Dearden P, Koentgen F, et al (2009) Effects of presynaptic mutations on a postsynaptic Cacna1s calcium channel colocalized with mGluR6 at mouse photoreceptor ribbon synapses. Invest Ophthalmol Vis Sci 50: 505–515. 18952919 10.1167/iovs.08-2758

[73] Kil TH, Kim JB (2010) Severe respiratory phenotype caused by a de novo Arg528Gly mutation in the CACNA1S gene in a patient with hypokalemic periodic paralysis. Eur J Paediatr Neurol 14: 278–281. 19822448 10.1016/j.ejpn.2009.08.004

[74] De Roo M, Klauser P, Garcia PM, Poglia L, Muller D (2008) Spine dynamics and synapse remodeling during LTP and memory processes. Prog Brain Res 169: 199–207. 18394475 10.1016/S0079-6123(07)00011-8

[75] Burnashev N (1998) Calcium permeability of ligand-gated channels. Cell Calcium 24: 325–332. 10091002 10.1016/s0143-4160(98)90056-2

[76] Curcic-Blake B, Swart M, Ter Horst GJ, Langers DR, Kema IP, et al (2012) Variation of the gene coding for DARPP-32 (PPP1R1B) and brain connectivity during associative emotional learning. Neuroimage 59: 1540–1550. 21878394 10.1016/j.neuroimage.2011.08.036

[77] Conner JM, Kulczycki M, Tuszynski MH (2010) Unique contributions of distinct cholinergic projections to motor cortical plasticity and learning. Cereb Cortex 20: 2739–2748. 20181623 10.1093/cercor/bhq022 PMC2951849

[78] Vanhaesebroeck B, Alessi DR (2000) The PI3K-PDK1 connection: more than just a road to PKB. Biochem J 346 (Pt 3) 561–576. 10698680 PMC1220886

[79] Bousquet M, Gibrat C, Saint-Pierre M, Julien C, Calon F, et al (2009) Modulation of brain-derived neurotrophic factor as a potential neuroprotective mechanism of action of omega-3 fatty acids in a parkinsonian animal model. Prog Neuropsychopharmacol Biol Psychiatry 33: 1401–1408. 19632286 10.1016/j.pnpbp.2009.07.018

[80] Cysneiros RM, Ferrari D, Arida RM, Terra VC, de Almeida AC, et al (2010) Qualitative analysis of hippocampal plastic changes in rats with epilepsy supplemented with oral omega-3 fatty acids. Epilepsy Behav 17: 33–38. 19969506 10.1016/j.yebeh.2009.11.006

[81] Kovalchuk Y, Hanse E, Kafitz KW, Konnerth A (2002) Postsynaptic Induction of BDNF-Mediated Long-Term Potentiation. Science 295: 1729–1734. 11872844 10.1126/science.1067766

[82] Zagrebelsky M, Holz A, Dechant G, Barde YA, Bonhoeffer T, et al (2005) The p75 neurotrophin receptor negatively modulates dendrite complexity and spine density in hippocampal neurons. J Neurosci 25: 9989–9999. 16251447 10.1523/JNEUROSCI.2492-05.2005 PMC6725571

[83] Woo NH, Teng HK, Siao CJ, Chiaruttini C, Pang PT, et al (2005) Activation of p75NTR by proBDNF facilitates hippocampal long-term depression. Nat Neurosci 8: 1069–1077. 16025106 10.1038/nn1510

[84] Hauck S, Kapczinski F, Roesler R, de Moura Silveira E Jr, Magalhaes PV, et al (2010) Serum brain-derived neurotrophic factor in patients with trauma psychopathology. Prog Neuropsychopharmacol Biol Psychiatry 34: 459–462. 20097247 10.1016/j.pnpbp.2010.01.010

[85] Takei S, Morinobu S, Yamamoto S, Fuchikami M, Matsumoto T, et al (2011) Enhanced hippocampal BDNF/TrkB signaling in response to fear conditioning in an animal model of posttraumatic stress disorder. J Psychiatr Res 45: 460–468. 20863519 10.1016/j.jpsychires.2010.08.009

[86] Lee R, Kermani P, Teng KK, Hempstead BL (2001) Regulation of cell survival by secreted proneurotrophins. Science 294: 1945–1948. 11729324 10.1126/science.1065057

[87] Liuzzi GM, Latronico T, Rossano R, Viggiani S, Fasano A, et al (2007) Inhibitory effect of polyunsaturated fatty acids on MMP-9 release from microglial cells–implications for complementary multiple sclerosis treatment. Neurochem Res 32: 2184–2193. 17624613 10.1007/s11064-007-9415-9

[88] Xu H, Gao HL, Zheng W, Xin N, Chi ZH, et al (2011) Lactational zinc deficiency-induced hippocampal neuronal apoptosis by a BDNF-independent TrkB signaling pathway. Hippocampus 21: 495–501. 20101602 10.1002/hipo.20767

[89] Kucharova K, Chang Y, Boor A, Yong VW, Stallcup WB (2011) Reduced inflammation accompanies diminished myelin damage and repair in the NG2 null mouse spinal cord. J Neuroinflammation 8: 158. 22078261 10.1186/1742-2094-8-158 PMC3229456

[90] Aboul-Enein F, Rauschka H, Kornek B, Stadelmann C, Stefferl A, et al (2003) Preferential loss of myelin-associated glycoprotein reflects hypoxia-like white matter damage in stroke and inflammatory brain diseases. J Neuropathol Exp Neurol 62: 25–33. 12528815 10.1093/jnen/62.1.25

[91] Caughey GE, Mantzioris E, Gibson RA, Cleland LG, James MJ (1996) The effect on human tumor necrosis factor alpha and interleukin 1 beta production of diets enriched in n-3 fatty acids from vegetable oil or fish oil. Am J Clin Nutr 63: 116–122. 8604658 10.1093/ajcn/63.1.116

[92] Endres S, Meydani SN, Ghorbani R, Schindler R, Dinarello CA (1993) Dietary supplementation with n-3 fatty acids suppresses interleukin-2 production and mononuclear cell proliferation. J Leukoc Biol 54: 599–603. 8245713

[93] Chandrasekar B, Fernandes G (1994) Decreased pro-inflammatory cytokines and increased antioxidant enzyme gene expression by omega-3 lipids in murine lupus nephritis. Biochem Biophys Res Commun 200: 893–898. 8179624 10.1006/bbrc.1994.1534

[94] Gorjao R, Azevedo-Martins AK, Rodrigues HG, Abdulkader F, Arcisio-Miranda M, et al (2009) Comparative effects of DHA and EPA on cell function. Pharmacol Ther 122: 56–64. 19318040 10.1016/j.pharmthera.2009.01.004

[95] Maes M, Christophe A, Bosmans E, Lin A, Neels H (2000) In humans, serum polyunsaturated fatty acid levels predict the response of proinflammatory cytokines to psychologic stress. Biol Psychiatry 47: 910–920. 10807964 10.1016/s0006-3223(99)00268-1

[96] Suk K, Lee H, Kang SS, Cho GJ, Choi WS (2003) Flavonoid baicalein attenuates activation-induced cell death of brain microglia. J Pharmacol Exp Ther 305: 638–645. 12606597 10.1124/jpet.102.047373

[97] Carlsen H, Haugen F, Zadelaar S, Kleemann R, Kooistra T, et al (2009) Diet-induced obesity increases NF-kappaB signaling in reporter mice. Genes Nutr 4: 215–222. 19707810 10.1007/s12263-009-0133-6 PMC2745749

[98] Rizzo AM, Corsetto PA, Montorfano G, Opizzi A, Faliva M, et al (2012) Comparison between the AA/EPA ratio in depressed and non depressed elderly females: omega-3 fatty acid supplementation correlates with improved symptoms but does not change immunological parameters. Nutr J 11: 82. 23046564 10.1186/1475-2891-11-82 PMC3499393

[99] Matsuda Y, Kusano H, Tsujimoto Y (1996) Chromosomal assignment of the Bcl2-related genes, Bcl2l and Bax, in the mouse and rat. Cytogenet Cell Genet 74: 107–110. 8893813 10.1159/000134393

[100] Zhang Y, Park TS, Gidday JM (2007) Hypoxic preconditioning protects human brain endothelium from ischemic apoptosis by Akt-dependent survivin activation. Am J Physiol Heart Circ Physiol 292: H2573–2581. 17337592 10.1152/ajpheart.01098.2006

[101] Muhie S, Hammamieh R, Cummings C, Yang D, Jett M (2013) Transcriptome characterization of immune suppression from battlefield-like stress. Genes Immun 14: 19–34. 23096155 10.1038/gene.2012.49 PMC3564018

[102] Alkhalil A, Hammamieh R, Hardick J, Ichou MA, Jett M, et al (2010) Gene expression profiling of monkeypox virus-infected cells reveals novel interfaces for host-virus interactions. Virol J 7: 173. 20667104 10.1186/1743-422X-7-173 PMC2920256

[103] Mills JD, Bailes JE, Sedney CL, Hutchins H, Sears B (2011) Omega-3 fatty acid supplementation and reduction of traumatic axonal injury in a rodent head injury model. J Neurosurg 114: 77–84. 20635852 10.3171/2010.5.JNS08914

[104] Mann JJ, Currier D (2012) Medication in Suicide Prevention Insights from Neurobiology of Suicidal Behavior. In: Dwivedi Y, editor. The Neurobiological Basis of Suicide. Boca Raton (FL). 23035282

[105] Torrey EF, Barci BM, Webster MJ, Bartko JJ, Meador-Woodruff JH, et al (2005) Neurochemical markers for schizophrenia, bipolar disorder, and major depression in postmortem brains. Biol Psychiatry 57: 252–260. 15691526 10.1016/j.biopsych.2004.10.019

[106] Marin Bivens CL, Grondahl C, Murray A, Blume T, Su YQ, et al (2004) Meiosis-activating sterol promotes the metaphase I to metaphase II transition and preimplantation developmental competence of mouse oocytes maturing in vitro. Biol Reprod 70: 1458–1464. 14736819 10.1095/biolreprod.103.026351

[107] Meyer JH, Kruger S, Wilson AA, Christensen BK, Goulding VS, et al (2001) Lower dopamine transporter binding potential in striatum during depression. Neuroreport 12: 4121–4125. 11742250 10.1097/00001756-200112210-00052

[108] Post RM (2010) Mechanisms of illness progression in the recurrent affective disorders. Neurotox Res 18: 256–271. 20390474 10.1007/s12640-010-9182-2

[109] Lorenzo A, Yuan M, Zhang Z, Paganetti PA, Sturchler-Pierrat C, et al (2000) Amyloid beta interacts with the amyloid precursor protein: a potential toxic mechanism in Alzheimer's disease. Nat Neurosci 3: 460–464. 10769385 10.1038/74833

[110] Blandini F, Sinforiani E, Pacchetti C, Samuele A, Bazzini E, et al (2006) Peripheral proteasome and caspase activity in Parkinson disease and Alzheimer disease. Neurology 66: 529–534. 16505307 10.1212/01.wnl.0000198511.09968.b3

[111] McGeer PL, McGeer EG (1995) The inflammatory response system of brain: implications for therapy of Alzheimer and other neurodegenerative diseases. Brain Res Brain Res Rev 21: 195–218. 8866675 10.1016/0165-0173(95)00011-9

[112] Wang D, Fu Q, Zhou Y, Xu B, Shi Q, et al (2013) beta2 adrenergic receptor, protein kinase A (PKA) and c-Jun N-terminal kinase (JNK) signaling pathways mediate tau pathology in Alzheimer's disease models. J Biol Chem 10.1074/jbc.M112.415141 PMC3624413 23430246

[113] Heck A, Vogler C, Gschwind L, Ackermann S, Auschra B, et al (2011) Statistical epistasis and functional brain imaging support a role of voltage-gated potassium channels in human memory. PLoS One 6: e29337. 22216252 10.1371/journal.pone.0029337 PMC3244442

[114] Sanchez-Resendis O, Medina AC, Serafin N, Prado-Alcala RA, Roozendaal B, et al (2012) Glucocorticoid-cholinergic interactions in the dorsal striatum in memory consolidation of inhibitory avoidance training. Front Behav Neurosci 6: 33. 22737110 10.3389/fnbeh.2012.00033 PMC3381328

[115] Takuma K, Mizoguchi H, Funatsu Y, Hoshina Y, Himeno Y, et al (2012) Combination of chronic stress and ovariectomy causes conditioned fear memory deficits and hippocampal cholinergic neuronal loss in mice. Neuroscience 207: 261–273. 22314316 10.1016/j.neuroscience.2012.01.034

[116] Kolassa IT, Ertl V, Eckart C, Glockner F, Kolassa S, et al (2010) Association study of trauma load and SLC6A4 promoter polymorphism in posttraumatic stress disorder: evidence from survivors of the Rwandan genocide. J Clin Psychiatry 71: 543–547. 20441718 10.4088/JCP.08m04787blu

[117] Gogolla N, Galimberti I, Deguchi Y, Caroni P (2009) Wnt signaling mediates experience-related regulation of synapse numbers and mossy fiber connectivities in the adult hippocampus. Neuron 62: 510–525. 19477153 10.1016/j.neuron.2009.04.022

[118] Uittenbogaard M, Baxter KK, Chiaramello A (2010) NeuroD6 genomic signature bridging neuronal differentiation to survival via the molecular chaperone network. J Neurosci Res 88: 33–54. 19610105 10.1002/jnr.22182 PMC2784025

[119] Prange O, Wong TP, Gerrow K, Wang YT, El-Husseini A (2004) A balance between excitatory and inhibitory synapses is controlled by PSD-95 and neuroligin. Proc Natl Acad Sci U S A 101: 13915–13920. 15358863 10.1073/pnas.0405939101 PMC518853

[120] Gao SF, Qi XR, Zhao J, Balesar R, Bao AM, et al (2012) Decreased NOS1 Expression in the Anterior Cingulate Cortex in Depression. Cereb Cortex 10.1093/cercor/bhs285 22989585

[121] Reif A, Grunblatt E, Herterich S, Wichart I, Rainer MK, et al (2011) Association of a functional NOS1 promoter repeat with Alzheimer's disease in the VITA cohort. J Alzheimers Dis 23: 327–333. 21098972 10.3233/JAD-2010-101491

[122] Cui H, Nishiguchi N, Yanagi M, Fukutake M, Mouri K, et al (2010) A putative cis-acting polymorphism in the NOS1 gene is associated with schizophrenia and NOS1 immunoreactivity in the postmortem brain. Schizophr Res 121: 172–178. 20605417 10.1016/j.schres.2010.05.003

[123] Silberberg G, Ben-Shachar D, Navon R (2010) Genetic analysis of nitric oxide synthase 1 variants in schizophrenia and bipolar disorder. Am J Med Genet B Neuropsychiatr Genet 153B: 1318–1328. 20645313 10.1002/ajmg.b.31112

[124] Grossetete M, Phelps J, Arko L, Yonas H, Rosenberg GA (2009) Elevation of matrix metalloproteinases 3 and 9 in cerebrospinal fluid and blood in patients with severe traumatic brain injury. Neurosurgery 65: 702–708. 19834375 10.1227/01.NEU.0000351768.11363.48 PMC2764327

[125] Rybakowski JK, Remlinger-Molenda A, Czech-Kucharska A, Wojcicka M, Michalak M, et al (2013) Increased serum matrix metalloproteinase-9 (MMP-9) levels in young patients during bipolar depression. J Affect Disord 146: 286–289. 22858217 10.1016/j.jad.2012.07.019

[126] Stomrud E, Bjorkqvist M, Janciauskiene S, Minthon L, Hansson O (2010) Alterations of matrix metalloproteinases in the healthy elderly with increased risk of prodromal Alzheimer's disease. Alzheimers Res Ther 2: 20. 20576109 10.1186/alzrt44 PMC2919700

[127] Lorenzl S, Albers DS, Relkin N, Ngyuen T, Hilgenberg SL, et al (2003) Increased plasma levels of matrix metalloproteinase-9 in patients with Alzheimer's disease. Neurochem Int 43: 191–196. 12689599 10.1016/s0197-0186(03)00004-4

[128] Rybakowski JK, Skibinska M, Kapelski P, Kaczmarek L, Hauser J (2009) Functional polymorphism of the matrix metalloproteinase-9 (MMP-9) gene in schizophrenia. Schizophr Res 109: 90–93. 19264454 10.1016/j.schres.2009.02.005

[129] Somerville MJ, Percy ME, Bergeron C, Yoong LK, Grima EA, et al (1991) Localization and quantitation of 68 kDa neurofilament and superoxide dismutase-1 mRNA in Alzheimer brains. Brain Res Mol Brain Res 9: 1–8. 1850065 10.1016/0169-328x(91)90123-f

[130] Gulesserian T, Seidl R, Hardmeier R, Cairns N, Lubec G (2001) Superoxide dismutase SOD1, encoded on chromosome 21, but not SOD2 is overexpressed in brains of patients with Down syndrome. J Investig Med 49: 41–46. 10.2310/6650.2001.34089 11217146

[131] Grassi-Oliveira R, Stein LM, Lopes RP, Teixeira AL, Bauer ME (2008) Low plasma brain-derived neurotrophic factor and childhood physical neglect are associated with verbal memory impairment in major depression–a preliminary report. Biol Psychiatry 64: 281–285. 18406398 10.1016/j.biopsych.2008.02.023

[132] Green EK, Raybould R, Macgregor S, Hyde S, Young AH, et al (2006) Genetic variation of brain-derived neurotrophic factor (BDNF) in bipolar disorder: case-control study of over 3000 individuals from the UK. Br J Psychiatry 188: 21–25. 16388065 10.1192/bjp.bp.105.009969

[133] Lee Y, Lim SW, Kim SY, Chung JW, Kim J, et al (2013) Association between the BDNF Val66Met Polymorphism and Chronicity of Depression. Psychiatry Investig 10: 56–61. 10.4306/pi.2013.10.1.56 PMC3590431 23482723

[134] Lin CH, Wu RM, Tai CH, Chen ML, Hu FC (2011) Lrrk2 S1647T and BDNF V66M interact with environmental factors to increase risk of Parkinson's disease. Parkinsonism Relat Disord 17: 84–88. 21167764 10.1016/j.parkreldis.2010.11.011

[135] Laske C, Stellos K, Hoffmann N, Stransky E, Straten G, et al (2011) Higher BDNF serum levels predict slower cognitive decline in Alzheimer's disease patients. Int J Neuropsychopharmacol 14: 399–404. 20860877 10.1017/S1461145710001008

[136] Binder EB, Bradley RG, Liu W, Epstein MP, Deveau TC, et al (2008) Association of FKBP5 polymorphisms and childhood abuse with risk of posttraumatic stress disorder symptoms in adults. JAMA 299: 1291–1305. 18349090 10.1001/jama.299.11.1291 PMC2441757

[137] Xie P, Kranzler HR, Poling J, Stein MB, Anton RF, et al (2010) Interaction of FKBP5 with childhood adversity on risk for post-traumatic stress disorder. Neuropsychopharmacology 35: 1684–1692. 20393453 10.1038/npp.2010.37 PMC2946626

[138] Kang JI, Chung HC, Jeung HC, Kim SJ, An SK, et al (2012) FKBP5 polymorphisms as vulnerability to anxiety and depression in patients with advanced gastric cancer: a controlled and prospective study. Psychoneuroendocrinology 37: 1569–1576. 22459275 10.1016/j.psyneuen.2012.02.017

[139] Menke A, Klengel T, Rubel J, Bruckl T, Pfister H, et al (2013) Genetic variation in FKBP5 associated with the extent of stress hormone dysregulation in major depression. Genes Brain Behav 10.1111/gbb.12026 23406438

