# Supplementary figures and images for: Transcriptomic Analysis of the Effects of a Fish Oil Enriched Diet on Murine Brains
Source: PLoS One. 2014 Mar 14;9(3):e90425. doi: 10.1371/journal.pone.0090425 (PMC3954562; doi:10.1371/journal.pone.0090425)

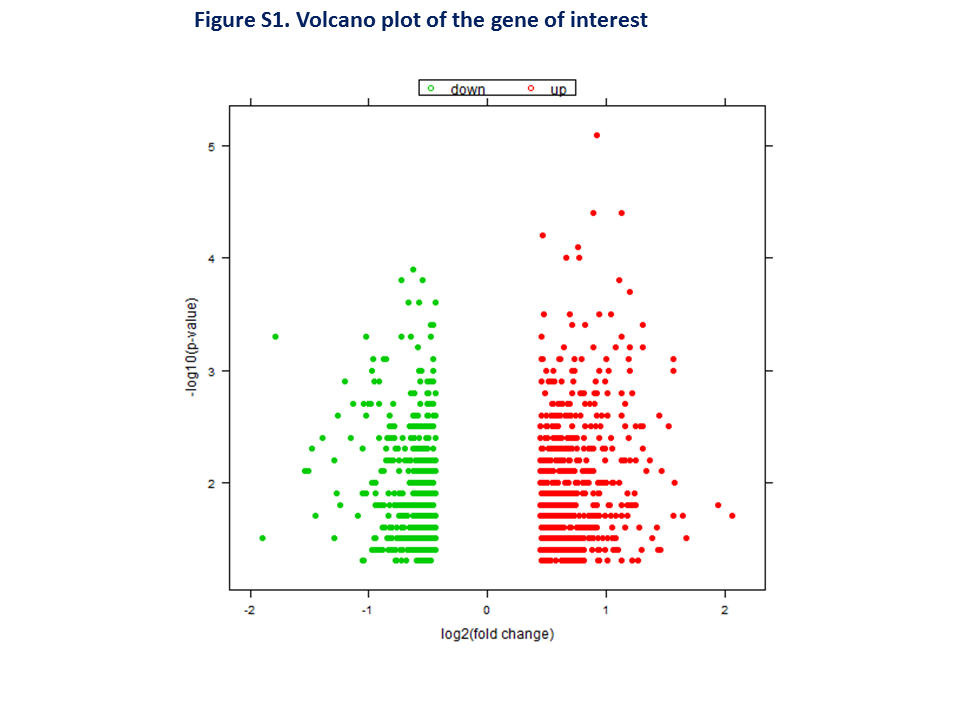

Supplement: Figure S1 — The volcano plot of the GOI depicting the expression distribution in FD and SD fed murine hemibrains. Here the x-axis depicts the log2(fold change) and y-axis depicts -log10(p-value), where p-value is calculated by FDR algorithm. The red and green dots represent the up- and down-regulated transcripts, respectively. (TIF) [file pone.0090425.s001.tif]

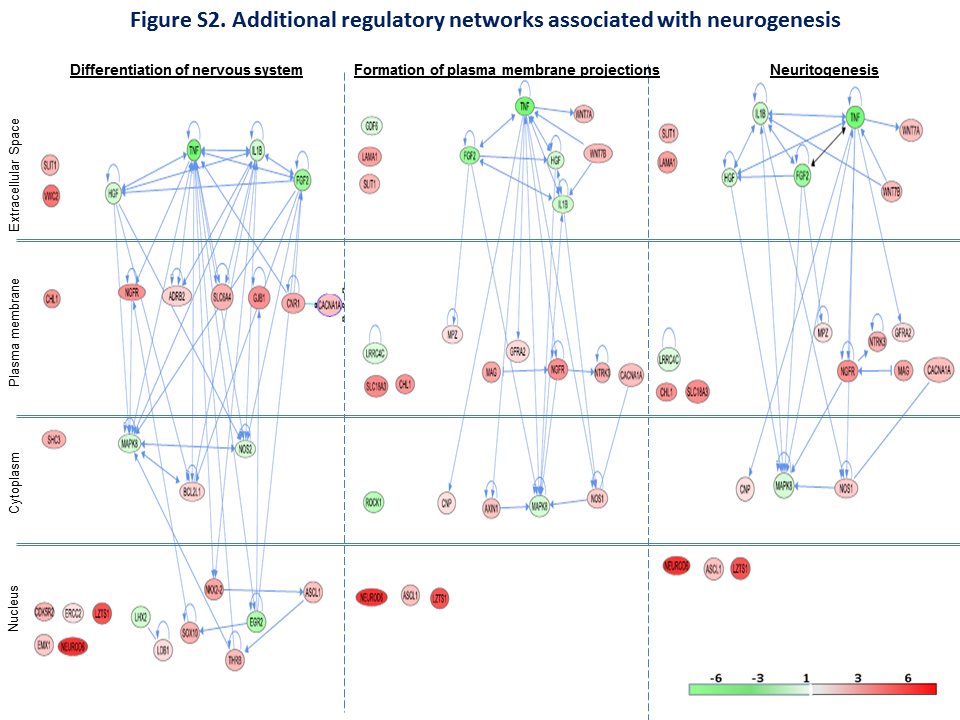

Supplement: Figure S2 — Regulatory networks showing an additional three clusters of differentially-regulated genes involved in “neurogenesis”. The nodes represent the genes and the solid lines depict the interactions between two molecules. Red- and green-colored genes show significantly increased and decreased expression (log1.5 (FD-/SD-fed mice) in the FD-fed mice compared to the SD-fed mice (a color scale is at the bottom right corner). The genes are clustered based on their proteins' cellular locations in the nucleus, cytoplasm, plasma membrane, and extracellular space. Three subgroups are “differentiation of nervous system” (left panel), “formation of plasma membrane projections” (middle panel) and “neuritogenesis” (right panel). They enlist 29, 26, and 23 genes with p values of 7.6×10^−23^, 4.71×10^−19^ and 8.02×10^−18^, respectively. (TIF) [file pone.0090425.s002.tif]
