# Supplementary material for: Transcriptomic Analysis of the Effects of a Fish Oil Enriched Diet on Murine Brains
Source: PLoS One. 2014 Mar 14;9(3):e90425. doi: 10.1371/journal.pone.0090425 (PMC3954562; doi:10.1371/journal.pone.0090425)
Supplement: Table S1 — List of primers and probes used in qPCR assay. (DOCX) [file pone.0090425.s003.docx]

# Table S1. List of primers and probes used in qPCR assay

| **Gene symbol** | **Sequence ( 3'-5')** | | |
| --- | --- | --- | --- |
|  | **Forward primer** | **Reverse primer** | **Probe** |
| NOS1 | CAAGACCCTGTGCGAGAT | GCACGATGTCATATTCCTCCAT | CGATGCCAAGGCTATGTCCA |
| FKBP5 | ACATTCCGATTGGGATCGAC | CGTACATAAGCTCAGCATTGG | TAAGTTTGGCATTGACCC |
| MMP9 | GGCTTAGATCATTCCAGCGT | TTAGAGCCACGACCATACAG | ACGGCATCCAGTATCTG |
| SOD1 | CGTACAATGGTGGTCCATGA | CCACACAGGGAATGTTTACTG | GAGTGATTGGGATTGCGC |
| BDNF | GCGCCCATGAAAGAAGT | CGTGCTCAAAAGTGTCAG | GACGACGACATCACTGGC |
