# Supplementary material for: Transcriptomic Analysis of the Effects of a Fish Oil Enriched Diet on Murine Brains
Source: PLoS One. 2014 Mar 14;9(3):e90425. doi: 10.1371/journal.pone.0090425 (PMC3954562; doi:10.1371/journal.pone.0090425)
Supplement: Table S2 — List of 1,142 genes of interest (GOI). The genes are identified by the Gene Name and gene symbol. The log ratio indicates the log1.5 transformed of the average transcriptomic expressions from FD-/SD-fed mice. (DOCX) [file pone.0090425.s004.docx]

# Table S2. List of 1142 genes of interest (GOI). The genes are identified by the Gene Name and gene symbol. The log ratio indicates the log1.5 transformed of the average transcriptomic expression from FD- / SD-fed mice.

| **Gene Name** | **Gene Symbol** | **Fold Change** |
| --- | --- | --- |
| NLR family, apoptosis inhibitory protein 6 | Naip6 | 3.90 |
| RFNG O-fucosylpeptide 3-beta-N-acetylglucosaminyltransferase | Rfng | 3.37 |
| cytochrome P450, family 27, subfamily a, polypeptide 1 | Cyp27a1 | 3.15 |
| family with sequence similarity 135, member B | Fam135b | 3.05 |
| hedgehog acyltransferase-like | Hhatl | 2.94 |
| oxoglutarate dehydrogenase-like | Ogdhl | 2.93 |
| potassium channel, subfamily K, member 12 | Kcnk12 | 2.92 |
| superoxide dismutase 3, extracellular | Sod3 | 2.88 |
| solute carrier family 18 (vesicular monoamine), member 3 | Slc18a3 | 2.83 |
| splicing factor 3a, subunit 2 | Sf3a2 | 2.79 |
| sialidase 4 | Neu4 | 2.76 |
| killer cell lectin-like receptor subfamily B member 1C | Klrb1c | 2.74 |
| EPS8-like 2 | Eps8l2 | 2.67 |
| aldehyde dehydrogenase family 7, member A1 | Aldh7a1 | 2.62 |
| G protein-coupled receptor 4 | Gpr4 | 2.59 |
| urocortin | Ucn | 2.58 |
| src homology three (SH3) and cysteine rich domain | Stac | 2.53 |
| proline rich 5 (renal) | Prr5 | 2.51 |
| ras homolog gene family, member V | Rhov | 2.44 |
| family with sequence similarity 110, member A | Fam110a | 2.44 |
| hairy/enhancer-of-split related with YRPW motif-like | Heyl | 2.43 |
| piccolo (presynaptic cytomatrix protein) | Pclo | 2.41 |
| RIKEN cDNA 9630041G16 gene | 9630041G16Rik | 2.37 |
| malate dehydrogenase 1B, NAD (soluble) | Mdh1b | 2.37 |
| cytochrome P450, family 3, subfamily a, polypeptide 11 | Cyp3a11 | 2.35 |
| cadherin 18 | Cdh18 | 2.35 |
| RIKEN cDNA 6030405A18 gene | 6030405A18Rik | 2.34 |
| basal cell adhesion molecule | Bcam | 2.33 |
| RIKEN cDNA B930041F14 gene | B930041F14Rik | 2.32 |
| sorting nexin 22 | Snx22 | 2.28 |
| melanoma antigen, family L, 2 | Magel2 | 2.28 |
| potassium inwardly-rectifying channel, subfamily J, member 4 | Kcnj4 | 2.28 |
| proline rich Gla (G-carboxyglutamic acid) 3 (transmembrane) | Prrg3 | 2.27 |
| fatty acid 2-hydroxylase | Fa2h | 2.25 |
| family with sequence similarity 5, member B | Fam5b | 2.23 |
| sterile alpha motif domain containing 1 | Samd1 | 2.23 |
| VPS10 domain receptor protein SORCS 1 | Sorcs1 | 2.22 |
| RIKEN cDNA 1500009C09 gene | 1500009C09Rik | 2.20 |
| purinergic receptor P2X, ligand-gated ion channel, 5 | P2rx5 | 2.19 |
| neurotensin receptor 1 | Ntsr1 | 2.19 |
| protein kinase C, gamma | Prkcc | 2.18 |
| calcium channel, voltage-dependent, gamma subunit 8 | Cacng8 | 2.18 |
| ring finger protein 152 | Rnf152 | 2.17 |
| potassium channel, subfamily K, member 3 | Kcnk3 | 2.16 |
| short stature homeobox 2 | Shox2 | 2.16 |
| cadherin 7, type 2 | Cdh7 | 2.16 |
| fascin homolog 1, actin bundling protein (Strongylocentrotus purpuratus) | Fscn1 | 2.15 |
| DNA binding protein with his-thr domain | Dbpht2 | 2.14 |
| transmembrane protein 82 | Tmem82 | 2.12 |
| RIKEN cDNA 4930539E08 gene | 4930539E08Rik | 2.12 |
| synuclein, gamma | Sncg | 2.12 |
| forkhead box P2 | Foxp2 | 2.11 |
| carbonic anhydrase 10 | Car10 | 2.08 |
| solute carrier family 17 (sodium-dependent inorganic phosphate cotransporter), member 6 | Slc17a6 | 2.07 |
| potassium inwardly rectifying channel, subfamily J, member 11 | Kcnj11 | 2.06 |
| tubulin tyrosine ligase-like family, member 9 | Ttll9 | 2.05 |
| complement component 1, q subcomponent-like 2 | C1ql2 | 2.02 |
| protein kinase C, delta | Prkcd | 2.02 |
| RIKEN cDNA 4932438H23 gene | 4932438H23Rik | 2.01 |
| leucine-rich repeats and transmembrane domains 2 | Lrtm2 | 2.00 |
| Smith-Magenis syndrome chromosome region, candidate 7 homolog (human) | Smcr7 | 1.99 |
| adhesion molecule with Ig like domain 1 | Amigo1 | 1.99 |
| gap junction protein, beta 1 | Gjb1 | 1.99 |
| zinc finger, DBF-type containing 2 | Zdbf2 | 1.98 |
| potassium channel, subfamily U, member 1 | Kcnu1 | 1.97 |
| nitrogen permease regulator-like 3 (S. cerevisiae) | Nprl3 | 1.96 |
| zinc figer protein 879 | Zfp879 | 1.95 |
| zinc finger protein 423 | Zfp423 | 1.95 |
| neuritin 1-like | Nrn1l | 1.92 |
| solute carrier family 25 (mitochondrial carrier), member 18 | Slc25a18 | 1.92 |
| calcium channel, voltage-dependent, gamma subunit 4 | Cacng4 | 1.91 |
| EF hand calcium binding domain 1 | Efcab1 | 1.91 |
| naked cuticle 2 homolog (Drosophila) | Nkd2 | 1.89 |
| G protein-coupled receptor 62 | Gpr62 | 1.89 |
| neurofascin | Nfasc | 1.86 |
| forkhead box P4 | Foxp4 | 1.85 |
| regulatory factor X, 4 (influences HLA class II expression) | Rfx4 | 1.84 |
| kelch domain containing 7A | Klhdc7a | 1.84 |
| myosin, heavy polypeptide 11, smooth muscle | Myh11 | 1.83 |
| dendrin | Ddn | 1.83 |
| RIKEN cDNA E030010A14 gene | E030010A14Rik | 1.83 |
| SRY-box containing gene 17 | Sox17 | 1.82 |
| junction plakoglobin | Jup | 1.82 |
| dual specificity phosphatase-like 15 | Dusp15 | 1.82 |
| progestin and adipoQ receptor family member VI | Paqr6 | 1.82 |
| tweety homolog 2 (Drosophila) | Ttyh2 | 1.81 |
| expressed sequence AI848285 | AI848285 | 1.81 |
| leucine rich repeat and Ig domain containing 4 | Lingo4 | 1.81 |
| G protein-coupled receptor 137 | Gpr137 | 1.81 |
| RIKEN cDNA C030046I01 gene | C030046I01Rik | 1.80 |
| solute carrier family 13 (sodium-dependent citrate transporter), member 5 | Slc13a5 | 1.80 |
| minichromosome maintenance deficient 2 mitotin (S. cerevisiae) | Mcm2 | 1.78 |
| acyl-Coenzyme A dehydrogenase, short chain | Acads | 1.78 |
| cysteine-rich secretory protein LCCL domain containing 2 | Crispld2 | 1.78 |
| Kv channel-interacting protein 1 | Kcnip1 | 1.78 |
| alkaline ceramidase 2 | Acer2 | 1.77 |
| protamine 1 | Prm1 | 1.76 |
| Fas apoptotic inhibitory molecule 2 | Faim2 | 1.76 |
| cat eye syndrome chromosome region, candidate 5 homolog (human) | Cecr5 | 1.76 |
| nucleus accumbens associated 2, BEN and BTB (POZ) domain containing | Nacc2 | 1.76 |
| guanylate binding protein 6 | Gbp6 | 1.75 |
| centromere protein B | Cenpb | 1.75 |
| U7 snRNP-specific Sm-like protein LSM11 | Lsm11 | 1.74 |
| chromatin modifying protein 6 | Chmp6 | 1.74 |
| BCL2-associated agonist of cell death | Bad | 1.74 |
| tripartite motif-containing 66 | Trim66 | 1.74 |
| RIKEN cDNA 9330161L09 gene | 9330161L09Rik | 1.73 |
| kelch repeat and BTB (POZ) domain containing 5 | Kbtbd5 | 1.73 |
| topoisomerase (DNA) III alpha | Top3a | 1.73 |
| galactose-3-O-sulfotransferase 1 | Gal3st1 | 1.73 |
| prostaglandin I2 (prostacyclin) synthase | Ptgis | 1.73 |
| follistatin-like 4 | Fstl4 | 1.72 |
| RIKEN cDNA 4930404N11 gene | 4930404N11Rik | 1.72 |
| glial cell line derived neurotrophic factor family receptor alpha 2 | Gfra2 | 1.72 |
| solute carrier family 8 (sodium/calcium exchanger), member 3 | Slc8a3 | 1.70 |
| MYST histone acetyltransferase (monocytic leukemia) 3 | Myst3 | 1.70 |
| carnosine synthase 1 | Carns1 | 1.70 |
| protein phosphatase 1, regulatory (inhibitor) subunit 1B | Ppp1r1b | 1.69 |
| solute carrier family 27 (fatty acid transporter), member 4 | Slc27a4 | 1.69 |
| excision repair cross-complementing rodent repair deficiency, complementation group 2 | Ercc2 | 1.69 |
| component of oligomeric golgi complex 8 | Cog8 | 1.68 |
| polymerase (RNA) III (DNA directed) polypeptide H | Polr3h | 1.68 |
| lipoma HMGIC fusion partner-like 5 | Lhfpl5 | 1.68 |
| C-type lectin domain family 18, member A | Clec18a | 1.67 |
| dihydropyrimidinase-like 5 | Dpysl5 | 1.65 |
| prodynorphin | Pdyn | 1.64 |
| homeobox B3 | Hoxb3 | 1.63 |
| SRY-box containing gene 10 | Sox10 | 1.63 |
| histocompatibility 2, class II antigen A, beta 1 | H2-Ab1 | 1.62 |
| tektin 5 | Tekt5 | 1.61 |
| potassium voltage gated channel, Shaw-related subfamily, member 3 | Kcnc3 | 1.61 |
| histocompatibility 2, class II antigen E beta | H2-Eb1 | 1.61 |
| angiopoietin-like 4 | Angptl4 | 1.60 |
| potassium voltage gated channel, Shaw-related subfamily, member 4 | Kcnc4 | 1.60 |
| crystallin, beta B1 | Crybb1 | 1.60 |
| sterile alpha and HEAT/Armadillo motif containing 1 | Sarm1 | 1.60 |
| lymphocyte specific 1 | Lsp1 | 1.60 |
| actinin alpha 2 | Actn2 | 1.59 |
| RIKEN cDNA D630003M21 gene | D630003M21Rik | 1.59 |
| protein phosphatase 1H (PP2C domain containing) | Ppm1h | 1.59 |
| abhydrolase domain containing 12 | Abhd12 | 1.59 |
| GPI anchor attachment protein 1 | Gpaa1 | 1.58 |
| tyrosine kinase, non-receptor, 2 | Tnk2 | 1.58 |
| guanosine diphosphate (GDP) dissociation inhibitor 1 | Gdi1 | 1.57 |
| predicted gene 10627 | Gm10627 | 1.57 |
| obscurin, cytoskeletal calmodulin and titin-interacting RhoGEF | Obscn | 1.57 |
| leucine rich repeat containing 3B | Lrrc3b | 1.56 |
| cysteine and glycine-rich protein 1 | Csrp1 | 1.56 |
| nerve growth factor receptor (TNFR superfamily, member 16) | Ngfr | 1.56 |
| sushi domain containing 2 | Susd2 | 1.56 |
| transmembrane and coiled-coil domains 2 | Tmcc2 | 1.56 |
| complexin 3 | Cplx3 | 1.55 |
| RIKEN cDNA C230096K16 gene | C230096K16Rik | 1.55 |
| RNA binding motif protein 45 | Rbm45 | 1.55 |
| procollagen-lysine, 2-oxoglutarate 5-dioxygenase 1 | Plod1 | 1.55 |
| huntingtin interacting protein 1 related | Hip1r | 1.55 |
| sarcoglycan, alpha (dystrophin-associated glycoprotein) | Sgca | 1.54 |
| mannosidase, alpha, class 1C, member 1 | Man1c1 | 1.54 |
| secernin 2 | Scrn2 | 1.54 |
| vacuolar protein sorting 37C (yeast) | Vps37c | 1.53 |
| steroid sulfatase | Sts | 1.53 |
| ubiquitin specific peptidase 5 (isopeptidase T) | Usp5 | 1.53 |
| insulin induced gene 1 | Insig1 | 1.53 |
| src homology 2 domain-containing transforming protein C3 | Shc3 | 1.52 |
| cannabinoid receptor 1 (brain) | Cnr1 | 1.52 |
| calpain, small subunit 1 | Capns1 | 1.52 |
| hypocretin (orexin) receptor 1 | Hcrtr1 | 1.51 |
| myelin-associated glycoprotein | Mag | 1.51 |
| beta-1,3-glucuronyltransferase 1 (glucuronosyltransferase P) | B3gat1 | 1.51 |
| phosphatidylinositol glycan anchor biosynthesis, class Z | Pigz | 1.51 |
| family with sequence similarity 73, member B | Fam73b | 1.51 |
| solute carrier family 9 (sodium/hydrogen exchanger), member 1 | Slc9a1 | 1.51 |
| serine (or cysteine) peptidase inhibitor, clade G, member 1 | Serping1 | 1.51 |
| kelch domain containing 8A | Klhdc8a | 1.51 |
| synaptogyrin 3 | Syngr3 | 1.50 |
| activin A receptor, type II-like 1 | Acvrl1 | 1.50 |
| non-protein coding RNA 86 | Ncrna00086 | 1.50 |
| arsA arsenite transporter, ATP-binding, homolog 1 (bacterial) | Asna1 | 1.49 |
| open reading frame 61 | ORF61 | 1.48 |
| alveolar soft part sarcoma chromosome region, candidate 1 (human) | Aspscr1 | 1.47 |
| solute carrier family 9 (sodium/hydrogen exchanger), member 3 regulator 1 | Slc9a3r1 | 1.47 |
| RIKEN cDNA 6030443J06 gene | 6030443J06Rik | 1.46 |
| glutamate receptor, ionotropic, NMDA2C (epsilon 3) | Grin2c | 1.46 |
| intermediate filament family orphan 2 | Iffo2 | 1.46 |
| tachykinin 2 | Tac2 | 1.46 |
| protein tyrosine phosphatase, receptor type, T | Ptprt | 1.46 |
| COX10 homolog, cytochrome c oxidase assembly protein, heme A: farnesyltransferase (yeast) | Cox10 | 1.45 |
| apolipoprotein B mRNA editing enzyme, catalytic polypeptide 2 | Apobec2 | 1.45 |
| anaplastic lymphoma kinase | Alk | 1.45 |
| adenomatosis polyposis coli 2 | Apc2 | 1.45 |
| TAF6 RNA polymerase II, TATA box binding protein (TBP)-associated factor | Taf6 | 1.45 |
| myeloid-associated differentiation marker-like 2 | Myadml2 | 1.44 |
| histocompatibility 2, blastocyst | H2-Bl | 1.44 |
| synaptopodin 2 | Synpo2 | 1.44 |
| solute carrier family 16 (monocarboxylic acid transporters), member 8 | Slc16a8 | 1.44 |
| Rho family GTPase 2 | Rnd2 | 1.43 |
| glutathione peroxidase 2 | Gpx2 | 1.43 |
| adenylate cyclase 5 | Adcy5 | 1.43 |
| death-associated protein kinase 3 | Dapk3 | 1.43 |
| sema domain, immunoglobulin domain (Ig), transmembrane domain (TM) and short cytoplasmic domain, (semaphorin) 4B | Sema4b | 1.42 |
| TBK1 binding protein 1 | Tbkbp1 | 1.42 |
| SCO-spondin | Sspo | 1.42 |
| transporter 1, ATP-binding cassette, sub-family B (MDR/TAP) | Tap1 | 1.41 |
| LIM domain binding 2 | Ldb2 | 1.41 |
| rhophilin, Rho GTPase binding protein 1 | Rhpn1 | 1.41 |
| slit homolog 1 (Drosophila) | Slit1 | 1.41 |
| RAS-like, family 10, member B | Rasl10b | 1.41 |
| adrenergic receptor, beta 2 | Adrb2 | 1.41 |
| predicted gene 10897 | Gm10897 | 1.40 |
| wingless-related MMTV integration site 7B | Wnt7b | 1.40 |
| RIKEN cDNA 2410131K14 gene | 2410131K14Rik | 1.40 |
| potassium voltage-gated channel, subfamily G, member 2 | Kcng2 | 1.40 |
| a disintegrin and metallopeptidase domain 18 | Adam18 | 1.39 |
| MAS-related GPR, member E | Mrgpre | 1.39 |
| transmembrane protein 204 | Tmem204 | 1.39 |
| transmembrane protein 91 | Tmem91 | 1.39 |
| bone morphogenetic protein 6 | Bmp6 | 1.39 |
| cDNA sequence BC029214 | BC029214 | 1.39 |
| gap junction protein, delta 3 | Gjd3 | 1.39 |
| nuclear receptor coactivator 5 | Ncoa5 | 1.39 |
| nudix (nucleoside diphosphate linked moiety X)-type motif 11 | Nudt11 | 1.38 |
| UBX domain protein 11 | Ubxn11 | 1.37 |
| desmin | Des | 1.37 |
| bicaudal D homolog 2 (Drosophila) | Bicd2 | 1.37 |
| a disintegrin-like and metallopeptidase (reprolysin type) with thrombospondin type 1 motif, 7 | Adamts7 | 1.37 |
| PNMA-like 1 | Pnmal1 | 1.37 |
| FtsJ homolog 2 (E. coli) | Ftsj2 | 1.36 |
| UPF1 regulator of nonsense transcripts homolog (yeast) | Upf1 | 1.36 |
| Na+/K+ transporting ATPase interacting 3 | Nkain3 | 1.36 |
| arginine decarboxylase | Adc | 1.36 |
| solute carrier family 16 (monocarboxylic acid transporters), member 9 | Slc16a9 | 1.35 |
| hyaluronan and proteoglycan link protein 4 | Hapln4 | 1.34 |
| LIM homeobox protein 1 | Lhx1 | 1.34 |
| Bernardinelli-Seip congenital lipodystrophy 2 homolog (human) | Bscl2 | 1.34 |
| zinc finger protein 651 | Zfp651 | 1.34 |
| NLR family member X1 | Nlrx1 | 1.34 |
| biogenesis of lysosome-related organelles complex-1, subunit 3 | Bloc1s3 | 1.34 |
| solute carrier family 17 (sodium-dependent inorganic phosphate cotransporter), member 8 | Slc17a8 | 1.34 |
| phosphatidylinositol transfer protein, membrane-associated 2 | Pitpnm2 | 1.34 |
| glutathione S-transferase, mu 6 | Gstm6 | 1.34 |
| BCL2-like 1 | Bcl2l1 | 1.34 |
| CCAAT/enhancer binding protein (C/EBP), alpha | Cebpa | 1.33 |
| ST6 (alpha-N-acetyl-neuraminyl-2,3-beta-galactosyl-1,3)-N-acetylgalactosaminide alpha-2,6-sialyltransferase 3 | St6galnac3 | 1.33 |
| mevalonate kinase | Mvk | 1.33 |
| hedgehog interacting protein-like 1 | Hhipl1 | 1.33 |
| calcium binding protein 1 | Cabp1 | 1.33 |
| potassium voltage-gated channel, subfamily H (eag-related), member 2 | Kcnh2 | 1.33 |
| outer dense fiber of sperm tails 3B | Odf3b | 1.32 |
| rhomboid, veinlet-like 1 (Drosophila) | Rhbdl1 | 1.32 |
| ER degradation enhancer, mannosidase alpha-like 2 | Edem2 | 1.32 |
| solute carrier family 25, member 42 | Slc25a42 | 1.32 |
| calbindin 2 | Calb2 | 1.31 |
| RecQ protein-like 5 | Recql5 | 1.31 |
| gap junction protein, alpha 4 | Gja4 | 1.31 |
| cathepsin F | Ctsf | 1.31 |
| fucosyltransferase 11 | Fut11 | 1.30 |
| RIKEN cDNA 6330512M04 gene | 6330512M04Rik | 1.30 |
| mesoderm induction early response 1, family member 2 | Mier2 | 1.30 |
| phosphatidylserine synthase 2 | Ptdss2 | 1.30 |
| vacuolar protein sorting 37D (yeast) | Vps37d | 1.29 |
| RIKEN cDNA A930038C07 gene | A930038C07Rik | 1.28 |
| Src homology 2 domain containing F | Shf | 1.28 |
| dysbindin (dystrobrevin binding protein 1) domain containing 1 | Dbndd1 | 1.28 |
| Vac14 homolog (S. cerevisiae) | Vac14 | 1.28 |
| chondroadherin-like | Chadl | 1.28 |
| wingless-type MMTV integration site 9B | Wnt9b | 1.28 |
| Parkinson disease 7 domain containing 1 | Pddc1 | 1.27 |
| opioid growth factor receptor | Ogfr | 1.27 |
| expressed sequence AI118078 | AI118078 | 1.27 |
| gastrokine 3 | Gkn3 | 1.27 |
| melanin-concentrating hormone receptor 1 | Mchr1 | 1.27 |
| rhomboid family 1 (Drosophila) | Rhbdf1 | 1.27 |
| lipopolysaccharide binding protein | Lbp | 1.27 |
| signal recognition particle 68 | Srp68 | 1.27 |
| coronin, actin binding protein 1B | Coro1b | 1.26 |
| fatty acid desaturase 2 | Fads2 | 1.26 |
| GIPC PDZ domain containing family, member 1 | Gipc1 | 1.26 |
| kin of IRRE like 3 (Drosophila) | Kirrel3 | 1.26 |
| hyperpolarization-activated, cyclic nucleotide-gated K+ 2 | Hcn2 | 1.26 |
| axin 1 | Axin1 | 1.25 |
| protein inhibitor of activated STAT 3 | Pias3 | 1.25 |
| RIKEN cDNA 2900064B16 gene | 2900064B16Rik | 1.25 |
| interferon regulatory factor 2 binding protein 1 | Irf2bp1 | 1.25 |
| prolyl-tRNA synthetase (mitochondrial)(putative) | Pars2 | 1.25 |
| cDNA sequence BC024139 | BC024139 | 1.25 |
| coiled-coil domain containing 9 | Ccdc9 | 1.25 |
| SH2 domain containing 3C | Sh2d3c | 1.24 |
| SH3-domain binding protein 2 | Sh3bp2 | 1.24 |
| inositol 1,3,4-triphosphate 5/6 kinase | Itpk1 | 1.24 |
| olfactory receptor 1269 | Olfr1269 | 1.24 |
| sparc/osteonectin, cwcv and kazal-like domains proteoglycan 1 | Spock1 | 1.24 |
| frizzled homolog 8 (Drosophila) | Fzd8 | 1.24 |
| RIKEN cDNA 9430041J12 gene | 9430041J12Rik | 1.24 |
| doublecortin domain containing 2a | Dcdc2a | 1.24 |
| empty spiracles homolog 1 (Drosophila) | Emx1 | 1.23 |
| lysozyme 1 | Lyz1 | 1.23 |
| methyltransferase like 7A2 | Mettl7a2 | 1.23 |
| Cbp/p300-interacting transactivator, with Glu/Asp-rich carboxy-terminal domain, 4 | Cited4 | 1.23 |
| olfactory receptor 921 | Olfr921 | 1.23 |
| FYVE, RhoGEF and PH domain containing 3 | Fgd3 | 1.23 |
| protein kinase N1 | Pkn1 | 1.23 |
| THO complex 5 | Thoc5 | 1.23 |
| serine protease inhibitor, Kunitz type 1 | Spint1 | 1.23 |
| target of myb1 homolog (chicken) | Tom1 | 1.22 |
| glutathione S-transferase, mu 4 | Gstm4 | 1.22 |
| Rho GTPase activating protein 27 | Arhgap27 | 1.22 |
| breast carcinoma amplified sequence 3 | Bcas3 | 1.22 |
| aspartoacylase | Aspa | 1.22 |
| F-box protein 44 | Fbxo44 | 1.22 |
| RIKEN cDNA 1700001O22 gene | 1700001O22Rik | 1.22 |
| glucose 6 phosphatase, catalytic, 3 | G6pc3 | 1.21 |
| glial fibrillary acidic protein | Gfap | 1.21 |
| Hermansky-Pudlak syndrome 1 homolog (human) | Hps1 | 1.21 |
| family with sequence similarity 116, member B | Fam116b | 1.21 |
| keratin 1 | Krt1 | 1.21 |
| potassium channel tetramerisation domain containing 17 | Kctd17 | 1.20 |
| chordin | Chrd | 1.20 |
| stearoyl-Coenzyme A desaturase 1 | Scd1 | 1.20 |
| rad and gem related GTP binding protein 2 | Rem2 | 1.20 |
| aminoacyl tRNA synthetase complex-interacting multifunctional protein 2 | Aimp2 | 1.20 |
| pecanex-like 3 (Drosophila) | Pcnxl3 | 1.20 |
| potassium voltage-gated channel, Shal-related family, member 3 | Kcnd3 | 1.20 |
| nuclear receptor interacting protein 2 | Nrip2 | 1.19 |
| peroxisomal biogenesis factor 10 | Pex10 | 1.19 |
| ceroid-lipofuscinosis, neuronal 6 | Cln6 | 1.19 |
| ancient ubiquitous protein 1 | Aup1 | 1.19 |
| quiescin Q6 sulfhydryl oxidase 1 | Qsox1 | 1.18 |
| WAP four-disulfide core domain 1 | Wfdc1 | 1.18 |
| GRB2-related adaptor protein | Grap | 1.18 |
| predicted gene 129 | Gm129 | 1.18 |
| ring finger protein 151 | Rnf151 | 1.18 |
| aldehyde dehydrogenase 1 family, member B1 | Aldh1b1 | 1.17 |
| endothelin converting enzyme-like 1 | Ecel1 | 1.17 |
| leukocyte tyrosine kinase | Ltk | 1.17 |
| eyes absent 2 homolog (Drosophila) | Eya2 | 1.17 |
| ribonuclease/angiogenin inhibitor 1 | Rnh1 | 1.17 |
| troponin I, skeletal, slow 1 | Tnni1 | 1.17 |
| HGF-regulated tyrosine kinase substrate | Hgs | 1.17 |
| thioredoxin reductase 2 | Txnrd2 | 1.17 |
| protein kinase domain containing, cytoplasmic | Pkdcc | 1.17 |
| ubiquitin-conjugating enzyme E2H | Ube2h | 1.17 |
| coiled-coil domain containing 155 | Ccdc155 | 1.17 |
| methyl-CpG binding domain protein 3 | Mbd3 | 1.16 |
| src homology 2 domain-containing transforming protein D | Shd | 1.16 |
| ninjurin 1 | Ninj1 | 1.16 |
| ankyrin repeat domain 54 | Ankrd54 | 1.16 |
| ras homolog gene family, member G | Rhog | 1.16 |
| immunoglobulin heavy constant mu | Ighm | 1.16 |
| B-cell CLL/lymphoma 7B | Bcl7b | 1.16 |
| glycosyltransferase 1 domain containing 1 | Glt1d1 | 1.15 |
| RIKEN cDNA 2900052N01 gene | 2900052N01Rik | 1.15 |
| low density lipoprotein receptor-related protein 3 | Lrp3 | 1.15 |
| leprecan 1 | Lepre1 | 1.15 |
| ankyrin repeat and SOCS box-containing 16 | Asb16 | 1.15 |
| REX1, RNA exonuclease 1 homolog (S. cerevisiae) | Rexo1 | 1.15 |
| abhydrolase domain containing 14b | Abhd14b | 1.15 |
| dimethylarginine dimethylaminohydrolase 1 | Ddah1 | 1.15 |
| methyltransferase like 13 | Mettl13 | 1.14 |
| RIKEN cDNA C030009O12 gene | C030009O12Rik | 1.14 |
| dynactin 2 | Dctn2 | 1.14 |
| N-acetyl galactosaminidase, alpha | Naga | 1.14 |
| protein phosphatase 1G (formerly 2C), magnesium-dependent, gamma isoform | Ppm1g | 1.14 |
| FK506 binding protein 4 | Fkbp4 | 1.14 |
| wingless-related MMTV integration site 7A | Wnt7a | 1.14 |
| proviral integration site 3 | Pim3 | 1.14 |
| PDZ domain containing 7 | Pdzd7 | 1.13 |
| oxysterol binding protein-like 6 | Osbpl6 | 1.13 |
| transmembrane protein 201 | Tmem201 | 1.13 |
| UDP galactosyltransferase 8A | Ugt8a | 1.13 |
| otoferlin | Otof | 1.13 |
| abhydrolase domain containing 16A | Abhd16a | 1.13 |
| patched domain containing 2 | Ptchd2 | 1.13 |
| RIKEN cDNA 1810055G02 gene | 1810055G02Rik | 1.13 |
| junctophilin 2 | Jph2 | 1.13 |
| pyridoxal (pyridoxine, vitamin B6) kinase, pseudogene | Pdxk-ps | 1.13 |
| thimet oligopeptidase 1 | Thop1 | 1.13 |
| calcium channel, voltage-dependent, beta 1 subunit | Cacnb1 | 1.12 |
| ADP-ribosylation factor GTPase activating protein 1 | Arfgap1 | 1.12 |
| guanine nucleotide binding protein (G protein), gamma 7 | Gng7 | 1.12 |
| unc-119 homolog (C. elegans) | Unc119 | 1.12 |
| F-box protein 27 | Fbxo27 | 1.12 |
| achaete-scute complex homolog 2 (Drosophila) | Ascl2 | 1.12 |
| transformation related protein 53 inducible protein 13 | Trp53i13 | 1.12 |
| enhancer of zeste homolog 1 (Drosophila) | Ezh1 | 1.12 |
| RIKEN cDNA 1110008J03 gene | 1110008J03Rik | 1.11 |
| zinc finger protein 385B | Zfp385b | 1.11 |
| G protein-coupled receptor 135 | Gpr135 | 1.11 |
| FIC domain containing | Ficd | 1.11 |
| peroxisome proliferator activator receptor delta | Ppard | 1.11 |
| RIKEN cDNA 2900079G21 gene | 2900079G21Rik | 1.11 |
| elongation factor Tu GTP binding domain containing 2 | Eftud2 | 1.11 |
| Na+/K+ transporting ATPase interacting 1 | Nkain1 | 1.11 |
| nuclear factor of activated T-cells, cytoplasmic, calcineurin-dependent 2 | Nfatc2 | 1.11 |
| RAB25, member RAS oncogene family | Rab25 | 1.11 |
| MLX interacting protein-like | Mlxipl | 1.10 |
| calcium/calmodulin-dependent protein kinase I gamma | Camk1g | 1.10 |
| peroxisomal biogenesis factor 19 | Pex19 | 1.10 |
| mitogen-activated protein kinase kinase kinase 11 | Map3k11 | 1.10 |
| limbic system-associated membrane protein | Lsamp | 1.09 |
| Kruppel-like factor 11 | Klf11 | 1.09 |
| cystathionine beta-synthase | Cbs | 1.09 |
| X-prolyl aminopeptidase (aminopeptidase P) 1, soluble | Xpnpep1 | 1.09 |
| UFM1-specific peptidase 1 | Ufsp1 | 1.09 |
| predicted pseudogene 5226 | Gm5226 | 1.09 |
| cDNA sequence BC053749 | BC053749 | 1.08 |
| lysine (K)-specific demethylase 4B | Kdm4b | 1.08 |
| v-maf musculoaponeurotic fibrosarcoma oncogene family, protein K (avian) | Mafk | 1.08 |
| zer-1 homolog (C. elegans) | Zer1 | 1.08 |
| pellino 3 | Peli3 | 1.08 |
| smoothelin | Smtn | 1.08 |
| ADP-ribosylation factor 5 | Arf5 | 1.08 |
| EF hand domain containing 1 | Efhd1 | 1.08 |
| ATP5S-like | Atp5sl | 1.08 |
| delta-like 4 (Drosophila) | Dll4 | 1.08 |
| folliculin | Flcn | 1.08 |
| potassium inwardly-rectifying channel, subfamily J, member 6 | Kcnj6 | 1.07 |
| adrenergic receptor, alpha 1d | Adra1d | 1.07 |
| adaptor-related protein complex 3, sigma 2 subunit | Ap3s2 | 1.07 |
| aggrecan | Acan | 1.07 |
| granzyme M (lymphocyte met-ase 1) | Gzmm | 1.07 |
| LIM domain binding 1 | Ldb1 | 1.07 |
| fibromodulin | Fmod | 1.07 |
| DNA segment, Chr 10, Johns Hopkins University 81 expressed | D10Jhu81e | 1.07 |
| dedicator of cyto-kinesis 3 | Dock3 | 1.07 |
| inter-alpha (globulin) inhibitor H5 | Itih5 | 1.06 |
| suppressor of defective silencing 3 homolog (S. cerevisiae) | Suds3 | 1.06 |
| MAD homolog 6 (Drosophila) | Smad6 | 1.06 |
| RIKEN cDNA 4930502E18 gene | 4930502E18Rik | 1.06 |
| DDB1 and CUL4 associated factor 15 | Dcaf15 | 1.06 |
| cathepsin D | Ctsd | 1.06 |
| RIKEN cDNA 2410002I01 gene | 2410002I01Rik | 1.06 |
| placental growth factor | Pgf | 1.06 |
| activin receptor IIB | Acvr2b | 1.05 |
| protease, serine, 41 | Prss41 | 1.05 |
| nudix (nucleoside diphosphate linked moiety X)-type motif 18 | Nudt18 | 1.05 |
| solute carrier family 16 (monocarboxylic acid transporters), member 2 | Slc16a2 | 1.05 |
| myelin protein zero | Mpz | 1.05 |
| MAM domain containing glycosylphosphatidylinositol anchor 1 | Mdga1 | 1.05 |
| secretory carrier membrane protein 5 | Scamp5 | 1.05 |
| PRA1 domain family 2 | Praf2 | 1.05 |
| prostaglandin E synthase 2 | Ptges2 | 1.05 |
| glycoprotein Ib, beta polypeptide | Gp1bb | 1.05 |
| ATG2 autophagy related 2 homolog A (S. cerevisiae) | Atg2a | 1.04 |
| solute carrier family 22 (organic anion transporter), member 6 | Slc22a6 | 1.04 |
| RAN GTPase activating protein 1 | Rangap1 | 1.04 |
| phospholipase A2, group VI | Pla2g6 | 1.04 |
| RIKEN cDNA 1700003E16 gene | 1700003E16Rik | 1.03 |
| DnaJ (Hsp40) homolog, subfamily B, member 5 | Dnajb5 | 1.03 |
| histocompatibility 2, D region locus 4 | H2-D4 | 1.03 |
| protein phosphatase 4, catalytic subunit | Ppp4c | 1.03 |
| solute carrier family 25 (mitochondrial carnitine/acylcarnitine translocase), member 20 | Slc25a20 | 1.03 |
| olfactory receptor 62 | Olfr62 | 1.02 |
| thromboxane A synthase 1, platelet | Tbxas1 | 1.02 |
| RIKEN cDNA A930001N09 gene | A930001N09Rik | 1.02 |
| tubulin tyrosine ligase-like family, member 10 | Ttll10 | 1.02 |
| mannose receptor, C type 2 | Mrc2 | 1.02 |
| serine/threonine kinase 33 | Stk33 | 1.02 |
| aspartic peptidase, retroviral-like 1 | Asprv1 | 1.01 |
| ribosomal protein SA | Rpsa | 1.01 |
| cytosolic thiouridylase subunit 2 homolog (S. pombe) | Ctu2 | 1.01 |
| calcium channel, voltage-dependent, gamma subunit 7 | Cacng7 | 1.01 |
| 2',3'-cyclic nucleotide 3' phosphodiesterase | Cnp | 1.01 |
| pygopus 2 | Pygo2 | 1.01 |
| small G protein signaling modulator 3 | Sgsm3 | 1.01 |
| cytochrome P450, family 4, subfamily f, polypeptide 13 | Cyp4f13 | 1.01 |
| integral membrane protein 2C | Itm2c | 1.01 |
| gastrulation brain homeobox 1 | Gbx1 | 1.01 |
| stabilin 1 | Stab1 | 1.01 |
| spermidine synthase | Srm | 1.01 |
| coiled-coil domain containing 151 | Ccdc151 | 1.00 |
| olfactory receptor 320 | Olfr320 | 1.00 |
| SWI/SNF related, matrix associated, actin dependent regulator of chromatin, subfamily d, member 2 | Smarcd2 | 1.00 |
| UDP-Gal:betaGlcNAc beta 1,3-galactosyltransferase, polypeptide 4 | B3galt4 | 1.00 |
| 3'-phosphoadenosine 5'-phosphosulfate synthase 2 | Papss2 | 1.00 |
| internexin neuronal intermediate filament protein, alpha | Ina | 1.00 |
| dynein light chain roadblock-type 2 | Dynlrb2 | 1.00 |
| fatty acid desaturase domain family, member 6 | Fads6 | 1.00 |
| jerky | Jrk | 1.00 |
| alpha-N-acetylglucosaminidase (Sanfilippo disease IIIB) | Naglu | 1.00 |
| endoplasmic reticulum (ER) to nucleus signalling 2 | Ern2 | 1.00 |
| H1 histone family, member X | H1fx | 1.00 |
| cytochrome P450, family 4, subfamily f, polypeptide 16 | Cyp4f16 | 1.00 |
| plexin D1 | Plxnd1 | 1.00 |
| slowmo homolog 1 (Drosophila) | Slmo1 | 1.00 |
| wingless-type MMTV integration site 9A | Wnt9a | 0.99 |
| predicted gene 4980 | Gm4980 | 0.99 |
| calcium channel, voltage-dependent, T type, alpha 1H subunit | Cacna1h | 0.99 |
| sarcoglycan zeta | Sgcz | 0.99 |
| reticulon 4 receptor-like 2 | Rtn4rl2 | 0.99 |
| mediator complex subunit 15 | Med15 | 0.99 |
| RIKEN cDNA 2310061I04 gene | 2310061I04Rik | 0.99 |
| sorting nexin 33 | Snx33 | 0.99 |
| nucleotide binding protein 2 | Nubp2 | 0.99 |
| Myb-related transcription factor, partner of profilin | Mypop | 0.99 |
| cortistatin | Cort | 0.99 |
| zinc finger protein 467 | Zfp467 | 0.98 |
| calcium channel, voltage-dependent, gamma subunit 2 | Cacng2 | 0.98 |
| programmed cell death 11 | Pdcd11 | 0.98 |
| glutathione S-transferase, theta 1 | Gstt1 | 0.98 |
| calcium channel, voltage-dependent, N type, alpha 1B subunit | Cacna1b | 0.98 |
| GM2 ganglioside activator protein | Gm2a | 0.98 |
| aminopeptidase-like 1 | Npepl1 | 0.98 |
| WD repeat domain 54 | Wdr54 | 0.97 |
| immunoglobulin superfamily, member 1 | Igsf1 | 0.97 |
| ATP-binding cassette, sub-family B (MDR/TAP), member 8 | Abcb8 | 0.97 |
| zinc finger protein 580 | Zfp580 | 0.97 |
| microtubule-associated protein 1 light chain 3 alpha | Map1lc3a | 0.97 |
| transducin (beta)-like 3 | Tbl3 | 0.97 |
| dishevelled associated activator of morphogenesis 2 | Daam2 | 0.96 |
| TNF receptor-associated protein 1 | Trap1 | 0.96 |
| G protein-coupled receptor 153 | Gpr153 | 0.96 |
| glucokinase | Gck | 0.96 |
| synaptopodin | Synpo | 0.96 |
| methyltransferase like 7A1 | Mettl7a1 | 0.96 |
| DEAD (Asp-Glu-Ala-Asp) box polypeptide 49 | Ddx49 | 0.96 |
| cyclin-dependent kinase 5 | Cdk5 | 0.95 |
| RanBP-type and C3HC4-type zinc finger containing 1 | Rbck1 | 0.95 |
| methyltransferase like 22 | Mettl22 | 0.95 |
| testis-specific serine kinase 1 | Tssk1 | 0.95 |
| growth arrest specific 8 | Gas8 | 0.95 |
| ilvB (bacterial acetolactate synthase)-like | Ilvbl | 0.95 |
| beta-1,3-glucuronyltransferase 3 (glucuronosyltransferase I) | B3gat3 | 0.95 |
| Max dimerization protein 4 | Mxd4 | 0.94 |
| v-rel reticuloendotheliosis viral oncogene homolog A (avian) | Rela | 0.94 |
| ring finger protein 5 | Rnf5 | 0.94 |
| lectin, galactoside-binding, soluble, 3 binding protein | Lgals3bp | 0.94 |
| DOM-3 homolog Z (C. elegans) | Dom3z | 0.94 |
| DPH1 homolog (S. cerevisiae) | Dph1 | 0.94 |
| leucine rich repeat containing 14 | Lrrc14 | 0.94 |
| within bgcn homolog (Drosophila) | Wibg | 0.94 |
| toll-like receptor adaptor molecule 1 | Ticam1 | 0.94 |
| basic helix-loop-helix family, member e41 | Bhlhe41 | 0.94 |
| WD repeat domain 18 | Wdr18 | 0.94 |
| cutA divalent cation tolerance homolog (E. coli) | Cuta | 0.94 |
| transforming growth factor beta regulated gene 1 | Tbrg1 | 0.94 |
| histocompatibility 2, D region locus 1 | H2-D1 | 0.93 |
| methylphosphate capping enzyme | Mepce | 0.93 |
| GPN-loop GTPase 2 | Gpn2 | 0.93 |
| kinesin light chain 4 | Klc4 | 0.93 |
| ephrin B3 | Efnb3 | 0.93 |
| E2F transcription factor 4 | E2f4 | 0.93 |
| BTB (POZ) domain containing 2 | Btbd2 | 0.93 |
| H2-K region expressed gene 6 | H2-Ke6 | 0.92 |
| tensin like C1 domain-containing phosphatase | Tenc1 | 0.92 |
| ADAMTS-like 4 | Adamtsl4 | 0.92 |
| seizure related 6 homolog like | Sez6l | 0.92 |
| dysbindin (dystrobrevin binding protein 1) domain containing 2 | Dbndd2 | 0.92 |
| TBC1 domain family, member 4 | Tbc1d4 | 0.92 |
| coiled-coil domain containing 157 | Ccdc157 | 0.92 |
| actinin alpha 3 | Actn3 | 0.92 |
| UDP-N-acetyl-alpha-D-galactosamine:polypeptide N-acetylgalactosaminyltransferase 6 | Galnt6 | 0.92 |
| schlafen 10, pseudogene | Slfn10-ps | 0.91 |
| olfactory receptor 975 | Olfr975 | 0.91 |
| AKT1 substrate 1 (proline-rich) | Akt1s1 | 0.91 |
| solute carrier family 38, member 10 | Slc38a10 | 0.91 |
| mitochondrial ribosomal protein L37 | Mrpl37 | 0.91 |
| ring finger protein 157 | Rnf157 | 0.91 |
| angio-associated migratory protein | Aamp | 0.91 |
| hypothetical protein 6530439I21 | 6530439I21 | 0.91 |
| WW domain binding protein 2 | Wbp2 | 0.91 |
| leucine rich repeat and sterile alpha motif containing 1 | Lrsam1 | 0.90 |
| chloride channel 6 | Clcn6 | 0.90 |
| WD repeat domain 8 | Wdr8 | 0.90 |
| F-box and WD-40 domain protein 9 | Fbxw9 | 0.90 |
| synaptotagmin XIII | Syt13 | 0.90 |
| polyhomeotic-like 2 (Drosophila) | Phc2 | 0.90 |
| acyl-Coenzyme A dehydrogenase family, member 10 | Acad10 | 0.90 |
| family with sequence similarity 108, member A | Fam108a | 0.90 |
| peptidyl arginine deiminase, type VI | Padi6 | 0.90 |
| polynucleotide kinase 3'- phosphatase | Pnkp | 0.89 |
| matrix metallopeptidase 24 | Mmp24 | 0.89 |
| cyclin-dependent kinase 4 | Cdk4 | 0.89 |
| ring finger protein 40 | Rnf40 | 0.89 |
| pyroglutamyl-peptidase I | Pgpep1 | 0.89 |
| ATPase type 13A1 | Atp13a1 | 0.89 |
| SRY-box containing gene 1 | Sox1 | 0.89 |
| golgi associated, gamma adaptin ear containing, ARF binding protein 1 | Gga1 | 0.89 |
| retinol saturase (all trans retinol 13,14 reductase) | Retsat | 0.88 |
| protein kinase, AMP-activated, beta 1 non-catalytic subunit | Prkab1 | 0.88 |
| solute carrier family 25, member 47 | Slc25a47 | 0.88 |
| solute carrier family 12 (potassium/chloride transporters), member 9 | Slc12a9 | 0.88 |
| solute carrier family 23 (nucleobase transporters), member 2 | Slc23a2 | 0.88 |
| N-myc downstream regulated gene 1 | Ndrg1 | 0.88 |
| translocase of inner mitochondrial membrane 50 homolog (yeast) | Timm50 | 0.88 |
| patatin-like phospholipase domain containing 2 | Pnpla2 | 0.87 |
| ring finger protein 212 | Rnf212 | 0.87 |
| predicted gene 684 | Gm684 | 0.87 |
| ubiquitin-conjugating enzyme E2Z (putative) | Ube2z | 0.87 |
| breast cancer metastasis-suppressor 1 | Brms1 | 0.87 |
| heparanase | Hpse | 0.87 |
| aldo-keto reductase family 7, member A5 (aflatoxin aldehyde reductase) | Akr7a5 | 0.87 |
| death effector domain-containing | Dedd | 0.87 |
| collagen, type XXV, alpha 1 | Col25a1 | 0.87 |
| olfactory receptor 237, pseudogene 1 | Olfr237-ps1 | 0.86 |
| melanoma associated antigen (mutated) 1 | Mum1 | 0.86 |
| polymerase (DNA-directed), delta 4 | Pold4 | 0.86 |
| pentatricopeptide repeat domain 1 | Ptcd1 | 0.86 |
| RIKEN cDNA 2610034B18 gene | 2610034B18Rik | 0.86 |
| dynactin 1 | Dctn1 | 0.86 |
| microspherule protein 1 | Mcrs1 | 0.85 |
| heat shock protein 8 | Hspb8 | 0.85 |
| Yip1 domain family, member 1 | Yipf1 | 0.85 |
| branched chain ketoacid dehydrogenase E1, alpha polypeptide | Bckdha | 0.84 |
| RAN binding protein 3 | Ranbp3 | 0.83 |
| sodium channel, voltage-gated, type I, beta | Scn1b | 0.83 |
| poly (ADP-ribose) polymerase family, member 3 | Parp3 | 0.83 |
| sorting nexin 17 | Snx17 | 0.83 |
| polymerase (DNA-directed), delta interacting protein 2 | Poldip2 | 0.83 |
| GLI-Kruppel family member GLI2 | Gli2 | 0.82 |
| expressed sequence AI413582 | AI413582 | 0.82 |
| insulin-like growth factor binding protein 2 | Igfbp2 | 0.81 |
| polymerase (RNA) III (DNA directed) polypeptide D | Polr3d | 0.80 |
| leucine zipper, down-regulated in cancer 1-like | Ldoc1l | 0.79 |
| RNA binding motif protein, X chromosome retrogene | Rbmxrt | -0.81 |
| enabled homolog (Drosophila) | Enah | -0.82 |
| coiled-coil domain containing 47 | Ccdc47 | -0.82 |
| copine VIII | Cpne8 | -0.82 |
| nucleoporin 88 | Nup88 | -0.83 |
| minichromosome maintenance deficient 4 homolog (S. cerevisiae) | Mcm4 | -0.84 |
| PHD finger protein 14 | Phf14 | -0.84 |
| WW domain binding protein 5 | Wbp5 | -0.84 |
| a disintegrin-like and metallopeptidase (reprolysin type) with thrombospondin type 1 motif, 9 | Adamts9 | -0.84 |
| nudix (nucleoside diphosphate linked moiety X)-type motif 5 | Nudt5 | -0.85 |
| Bardet-Biedl syndrome 5 (human) | Bbs5 | -0.85 |
| DEAD (Asp-Glu-Ala-Asp) box polypeptide 42 | Ddx42 | -0.85 |
| oxidative stress induced growth inhibitor family member 2 | Osgin2 | -0.85 |
| tetratricopeptide repeat domain 8 | Ttc8 | -0.86 |
| ephrin A5 | Efna5 | -0.86 |
| poly (ADP-ribose) polymerase family, member 10 | Parp10 | -0.86 |
| origin recognition complex, subunit 2 | Orc2 | -0.86 |
| RIKEN cDNA B230354K17 gene | B230354K17Rik | -0.86 |
| RAD17 homolog (S. pombe) | Rad17 | -0.86 |
| dynamin 1-like | Dnm1l | -0.86 |
| zinc finger and BTB domain containing 6 | Zbtb6 | -0.86 |
| RIKEN cDNA 1600012F09 gene | 1600012F09Rik | -0.86 |
| solute carrier family 39 (metal ion transporter), member 6 | Slc39a6 | -0.86 |
| coiled-coil domain containing 46 | Ccdc46 | -0.86 |
| ribosomal protein S28 | Rps28 | -0.87 |
| met proto-oncogene | Met | -0.87 |
| RIKEN cDNA 1110067D22 gene | 1110067D22Rik | -0.87 |
| ninein | Nin | -0.87 |
| vomeronasal 1 receptor 42 | Vmn1r42 | -0.87 |
| cell adhesion molecule 1 | Cadm1 | -0.87 |
| AF4/FMR2 family, member 4 | Aff4 | -0.87 |
| ankyrin repeat and KH domain containing 1 | Ankhd1 | -0.87 |
| pleckstrin homology domain interacting protein | Phip | -0.88 |
| nemo like kinase | Nlk | -0.88 |
| hypothetical LOC100504038 | LOC100504038 | -0.88 |
| jumonji C domain-containing histone demethylase 1 homolog D (S. cerevisiae) | Jhdm1d | -0.88 |
| trichohyalin | Tchh | -0.88 |
| RIKEN cDNA B130006D01 gene | B130006D01Rik | -0.89 |
| RAB22A, member RAS oncogene family | Rab22a | -0.89 |
| predicted gene 5622 | Gm5622 | -0.89 |
| angel homolog 2 (Drosophila) | Angel2 | -0.90 |
| meiosis-specific nuclear structural protein 1 | Mns1 | -0.90 |
| RIKEN cDNA 2810427C15 gene | 2810427C15Rik | -0.90 |
| RIKEN cDNA C920008N22 gene | C920008N22Rik | -0.90 |
| UTP3, small subunit (SSU) processome component, homolog (S. cerevisiae) | Utp3 | -0.90 |
| ligase IV, DNA, ATP-dependent | Lig4 | -0.90 |
| DNA primase, p49 subunit | Prim1 | -0.90 |
| zinc finger, MYND domain containing 17 | Zmynd17 | -0.90 |
| predicted gene 7968 | Gm7968 | -0.90 |
| baculoviral IAP repeat-containing 3 | Birc3 | -0.90 |
| nuclear export mediator factor | Nemf | -0.90 |
| eukaryotic translation initiation factor 5B | Eif5b | -0.91 |
| integrin alpha L | Itgal | -0.91 |
| transient receptor potential cation channel, subfamily C, member 2 | Trpc2 | -0.91 |
| GUF1 GTPase homolog (S. cerevisiae) | Guf1 | -0.91 |
| N-acetylneuraminic acid phosphatase | Nanp | -0.91 |
| zinc finger protein 68 | Zfp68 | -0.91 |
| DnaJ (Hsp40) homolog, subfamily C, member 1 | Dnajc1 | -0.91 |
| ornithine decarboxylase, structural 1 | Odc1 | -0.91 |
| centrosomal protein 290 | Cep290 | -0.91 |
| KH domain containing, RNA binding, signal transduction associated 1 | Khdrbs1 | -0.91 |
| RIKEN cDNA E330016A19 gene | E330016A19Rik | -0.92 |
| ELAV (embryonic lethal, abnormal vision, Drosophila)-like 2 (Hu antigen B) | Elavl2 | -0.92 |
| eukaryotic translation elongation factor 1 epsilon 1 | Eef1e1 | -0.92 |
| triadin | Trdn | -0.92 |
| lymphocyte antigen 6 complex, locus I | Ly6i | -0.92 |
| transmembrane protein 126A | Tmem126a | -0.92 |
| poliovirus receptor | Pvr | -0.92 |
| peptidylprolyl isomerase (cyclophilin)-like 4 | Ppil4 | -0.92 |
| heat shock protein 90, alpha (cytosolic), class A member 1 | Hsp90aa1 | -0.92 |
| RIKEN cDNA 4933427D14 gene | 4933427D14Rik | -0.92 |
| RIKEN cDNA 5730469M10 gene | 5730469M10Rik | -0.92 |
| PHD finger protein 17 | Phf17 | -0.92 |
| high-mobility group nucleosome binding domain 5 | Hmgn5 | -0.92 |
| v-abl Abelson murine leukemia viral oncogene homolog 2 (arg, Abelson-related gene) | Abl2 | -0.92 |
| cDNA sequence U90926 | U90926 | -0.92 |
| SET domain containing 5 | Setd5 | -0.92 |
| transcription factor B2, mitochondrial | Tfb2m | -0.93 |
| H2A histone family, member Z | H2afz | -0.93 |
| hydroxysteroid (17-beta) dehydrogenase 11 | Hsd17b11 | -0.93 |
| SWI/SNF related, matrix associated, actin dependent regulator of chromatin, subfamily a, member 1 | Smarca1 | -0.93 |
| sorbin and SH3 domain containing 1 | Sorbs1 | -0.94 |
| coiled-coil domain containing 53 | Ccdc53 | -0.94 |
| UHRF1 (ICBP90) binding protein 1-like | Uhrf1bp1l | -0.94 |
| PTC7 protein phosphatase homolog (S. cerevisiae) | Pptc7 | -0.94 |
| RIKEN cDNA C030046E11 gene | C030046E11Rik | -0.94 |
| THAP domain containing, apoptosis associated protein 2 | Thap2 | -0.95 |
| zinc finger, CCHC domain containing 11 | Zcchc11 | -0.95 |
| pleckstrin homology domain-containing, family A (phosphoinositide binding specific) member 2 | Plekha2 | -0.95 |
| RIKEN cDNA C330019G07 gene | C330019G07Rik | -0.96 |
| mindbomb homolog 1 (Drosophila) | Mib1 | -0.96 |
| ubiquitin A-52 residue ribosomal protein fusion product 1 | Uba52 | -0.96 |
| protogenin homolog (Gallus gallus) | Prtg | -0.96 |
| NADH dehydrogenase (ubiquinone) 1 beta subcomplex, 7 | Ndufb7 | -0.96 |
| RIKEN cDNA A430057L12 gene | A430057L12Rik | -0.96 |
| myeloid cell nuclear differentiation antigen | Mnda | -0.97 |
| protein tyrosine phosphatase, non-receptor type 4 | Ptpn4 | -0.97 |
| keratin 15 | Krt15 | -0.97 |
| synapse associated protein 1 | Syap1 | -0.97 |
| predicted gene 9897 | Gm9897 | -0.97 |
| RIKEN cDNA 1110002N22 gene | 1110002N22Rik | -0.97 |
| fibronectin type III domain containing 1 | Fndc1 | -0.97 |
| heterogeneous nuclear ribonucleoprotein A/B | Hnrnpab | -0.97 |
| deafness, autosomal dominant 5 (human) | Dfna5 | -0.97 |
| heterogeneous nuclear ribonucleoprotein H3 | Hnrnph3 | -0.97 |
| caspase 7 | Casp7 | -0.98 |
| RIKEN cDNA 2010107E04 gene | 2010107E04Rik | -0.98 |
| melanoma antigen, family A, 5 | Magea5 | -0.98 |
| pyruvate dehyrogenase phosphatase catalytic subunit 1 | Pdp1 | -0.98 |
| exocyst complex component 5 | Exoc5 | -0.98 |
| RIKEN cDNA E330037G11 gene | E330037G11Rik | -0.98 |
| dynactin 3 | Dctn3 | -0.98 |
| zinc finger protein 189 | Zfp189 | -0.98 |
| ribosomal protein L35 | Rpl35 | -0.99 |
| tripartite motif-containing 33 | Trim33 | -0.99 |
| BTB and CNC homology 2 | Bach2 | -0.99 |
| tumor necrosis factor receptor superfamily, member 22 | Tnfrsf22 | -0.99 |
| hypothetical protein LOC100504100 | LOC100504100 | -0.99 |
| DEAH (Asp-Glu-Ala-His) box polypeptide 33 | Dhx33 | -1.00 |
| vomeronasal 1 receptor 47 | Vmn1r47 | -1.00 |
| lysyl oxidase | Lox | -1.00 |
| myeloid/lymphoid or mixed-lineage leukemia 5 | Mll5 | -1.01 |
| very low density lipoprotein receptor | Vldlr | -1.01 |
| ring finger protein 125 | Rnf125 | -1.01 |
| synaptojanin 2 binding protein | Synj2bp | -1.01 |
| RIKEN cDNA B830007D08 gene | B830007D08Rik | -1.01 |
| IK cytokine | Ik | -1.01 |
| ubiquitin associated and SH3 domain containing, B | Ubash3b | -1.01 |
| SEC16 homolog B (S. cerevisiae) | Sec16b | -1.01 |
| RAS, guanyl releasing protein 3 | Rasgrp3 | -1.02 |
| NHL repeat containing 2 | Nhlrc2 | -1.02 |
| SMC hinge domain containing 1 | Smchd1 | -1.02 |
| Rho GTPase activating protein 6 | Arhgap6 | -1.02 |
| vomeronasal 1 receptor 236 | Vmn1r236 | -1.02 |
| guanylate cyclase activator 2a (guanylin) | Guca2a | -1.02 |
| coiled-coil domain containing 90A | Ccdc90a | -1.03 |
| tripartite motif-containing 24 | Trim24 | -1.03 |
| protein kinase, X-linked | Prkx | -1.03 |
| WD repeat domain 78 | Wdr78 | -1.03 |
| synaptonemal complex central element protein 1 | Syce1 | -1.03 |
| biogenesis of lysosome-related organelles complex-1, subunit 1 | Bloc1s1 | -1.03 |
| zinc finger, MYM-type 5 | Zmym5 | -1.03 |
| Sp110 nuclear body protein | Sp110 | -1.03 |
| F-box and WD-40 domain protein 7 | Fbxw7 | -1.03 |
| cDNA sequence AY036118 | AY036118 | -1.03 |
| cathepsin L | Ctsl | -1.04 |
| methylmalonyl-Coenzyme A mutase | Mut | -1.04 |
| RELT-like 1 | Rell1 | -1.04 |
| zinc finger protein 251 | Zfp251 | -1.04 |
| TATA box binding protein (Tbp)-associated factor, RNA polymerase I, D | Taf1d | -1.04 |
| ESF1, nucleolar pre-rRNA processing protein, homolog (S. cerevisiae) | Esf1 | -1.04 |
| nuclear receptor interacting protein 1 | Nrip1 | -1.04 |
| progesterone immunomodulatory binding factor 1 | Pibf1 | -1.04 |
| THO complex 2 | Thoc2 | -1.04 |
| Crn, crooked neck-like 1 (Drosophila) | Crnkl1 | -1.04 |
| inositol 1,4,5-triphosphate receptor 2 | Itpr2 | -1.05 |
| ATPase, Na+/K+ transporting, alpha 1 polypeptide | Atp1a1 | -1.05 |
| RIKEN cDNA D630033O11 gene | D630033O11Rik | -1.05 |
| centromere protein E | Cenpe | -1.05 |
| insulin receptor substrate 1 | Irs1 | -1.05 |
| G protein-coupled receptor 126 | Gpr126 | -1.05 |
| brain derived neurotrophic factor | Bdnf | -1.05 |
| family with sequence similarity 60, member A | Fam60a | -1.06 |
| SET binding protein 1 | Setbp1 | -1.06 |
| mitogen-activated protein kinase 8 | Mapk8 | -1.06 |
| SUMO1/sentrin specific peptidase 7 | Senp7 | -1.06 |
| Wolf-Hirschhorn syndrome candidate 1 (human) | Whsc1 | -1.06 |
| nuclear autoantigenic sperm protein (histone-binding) | Nasp | -1.06 |
| hephaestin | Heph | -1.07 |
| Fanconi anemia, complementation group D2 | Fancd2 | -1.07 |
| RIKEN cDNA 5830409B07 gene | 5830409B07Rik | -1.07 |
| sprouty homolog 2 (Drosophila) | Spry2 | -1.07 |
| acyl-CoA synthetase long-chain family member 4 | Acsl4 | -1.07 |
| fibronectin type III domain containing 3B | Fndc3b | -1.08 |
| RIKEN cDNA A330021E22 gene | A330021E22Rik | -1.08 |
| CD302 antigen | Cd302 | -1.08 |
| cDNA sequence BC026762 | BC026762 | -1.08 |
| SUMO/sentrin specific peptidase 6 | Senp6 | -1.08 |
| asialoglycoprotein receptor 2 | Asgr2 | -1.09 |
| eukaryotic translation initiation factor 2, subunit 3, structural gene Y-linked | Eif2s3y | -1.09 |
| homer homolog 1 (Drosophila) | Homer1 | -1.09 |
| centrosomal protein 70 | Cep70 | -1.09 |
| metal response element binding transcription factor 2 | Mtf2 | -1.09 |
| kinectin 1 | Ktn1 | -1.09 |
| ADP-ribosylation factor-like 6 interacting protein 1 | Arl6ip1 | -1.09 |
| early growth response 1 | Egr1 | -1.09 |
| family with sequence similarity 199, X-linked | Fam199x | -1.09 |
| calmodulin regulated spectrin-associated protein 1 | Camsap1 | -1.10 |
| predicted gene 15506 | Gm15506 | -1.10 |
| armadillo repeat containing, X-linked 4 | Armcx4 | -1.10 |
| cytotoxic granule-associated RNA binding protein 1 | Tia1 | -1.10 |
| lysosomal-associated protein transmembrane 4A | Laptm4a | -1.10 |
| NLR family, pyrin domain containing 10 | Nlrp10 | -1.10 |
| HAUS augmin-like complex, subunit 6 | Haus6 | -1.10 |
| ladybird homeobox homolog 2 (Drosophila) | Lbx2 | -1.11 |
| ganglioside-induced differentiation-associated-protein 10 | Gdap10 | -1.11 |
| periplakin | Ppl | -1.11 |
| predicted gene 7890 | Gm7890 | -1.11 |
| superoxide dismutase 1, soluble | Sod1 | -1.11 |
| runt-related transcription factor 1; translocated to, 1 (cyclin D-related) | Runx1t1 | -1.11 |
| 3-hydroxyisobutyryl-Coenzyme A hydrolase | Hibch | -1.12 |
| ring finger protein 168 | Rnf168 | -1.12 |
| RIKEN cDNA 1110035E04 gene | 1110035E04Rik | -1.12 |
| bromodomain PHD finger transcription factor | Bptf | -1.13 |
| ubinuclein 2 | Ubn2 | -1.13 |
| ribosomal protein L12 | Rpl12 | -1.13 |
| RIO kinase 1 (yeast) | Riok1 | -1.13 |
| neutral sphingomyelinase (N-SMase) activation associated factor | Nsmaf | -1.13 |
| testis expressed gene 19.2 | Tex19.2 | -1.13 |
| Wilms' tumour 1-associating protein | Wtap | -1.14 |
| RIKEN cDNA A430089I19 gene | A430089I19Rik | -1.14 |
| receptor transporter protein 3 | Rtp3 | -1.14 |
| choroidermia | Chm | -1.14 |
| Rho GTPase activating protein 8 | Arhgap8 | -1.14 |
| calcium binding and coiled-coil domain 2 | Calcoco2 | -1.14 |
| spermatid associated | Spert | -1.15 |
| inhibitor of growth family, member 5 | Ing5 | -1.15 |
| mitogen-activated protein kinase 1 interacting protein 1 | Mapk1ip1 | -1.15 |
| family with sequence similarity 169, member A | Fam169a | -1.15 |
| PDZ domain containing RING finger 3 | Pdzrn3 | -1.15 |
| protein phosphatase 2, regulatory subunit B (B56), alpha isoform | Ppp2r5a | -1.16 |
| Snf2-related CREBBP activator protein | Srcap | -1.16 |
| LIM homeobox protein 2 | Lhx2 | -1.16 |
| transcription termination factor, RNA polymerase II | Ttf2 | -1.16 |
| PHD finger protein 19 | Phf19 | -1.16 |
| caspase recruitment domain family, member 14 | Card14 | -1.17 |
| cell division cycle and apoptosis regulator 1 | Ccar1 | -1.17 |
| ephrin A4 | Efna4 | -1.17 |
| receptor accessory protein 3 | Reep3 | -1.17 |
| cytochrome c oxidase, subunit VI a, polypeptide 1 | Cox6a1 | -1.17 |
| RIKEN cDNA B930063P07 gene | B930063P07Rik | -1.17 |
| zinc finger protein 131 | Zfp131 | -1.17 |
| sorting nexin 7 | Snx7 | -1.18 |
| LON peptidase N-terminal domain and ring finger 2 | Lonrf2 | -1.18 |
| Wiskott-Aldrich syndrome homolog (human) | Was | -1.19 |
| RIKEN cDNA A430106A12 gene | A430106A12Rik | -1.19 |
| galactose mutarotase | Galm | -1.19 |
| calcium channel, voltage-dependent, L type, alpha 1S subunit | Cacna1s | -1.19 |
| KCNQ1 overlapping transcript 1 | Kcnq1ot1 | -1.19 |
| metallothionein-like 5, testis-specific (tesmin) | Mtl5 | -1.20 |
| protein disulfide isomerase associated 5 | Pdia5 | -1.20 |
| spindle assembly 6 homolog (C. elegans) | Sass6 | -1.21 |
| cyclin T2 | Ccnt2 | -1.21 |
| heterogeneous nuclear ribonucleoprotein A2/B1 | Hnrnpa2b1 | -1.21 |
| RNA binding motif protein 28 | Rbm28 | -1.21 |
| caspase 1 | Casp1 | -1.22 |
| LON peptidase N-terminal domain and ring finger 3 | Lonrf3 | -1.22 |
| C-type lectin domain family 2, member g | Clec2g | -1.22 |
| ribosomal protein S4, X-linked | Rps4x | -1.22 |
| TBC1 domain family, member 10c | Tbc1d10c | -1.23 |
| leucine-rich repeats and immunoglobulin-like domains 2 | Lrig2 | -1.23 |
| TNF receptor-associated factor 1 | Traf1 | -1.23 |
| Rho-associated coiled-coil containing protein kinase 1 | Rock1 | -1.23 |
| AT rich interactive domain 2 (ARID, RFX-like) | Arid2 | -1.23 |
| RIKEN cDNA 4921524J17 gene | 4921524J17Rik | -1.24 |
| fibronectin leucine rich transmembrane protein 3 | Flrt3 | -1.25 |
| solute carrier family 39 (metal ion transporter), member 8 | Slc39a8 | -1.26 |
| AHA1, activator of heat shock protein ATPase homolog 2 (yeast) | Ahsa2 | -1.26 |
| sushi, von Willebrand factor type A, EGF and pentraxin domain containing 1 | Svep1 | -1.27 |
| echinoderm microtubule associated protein like 5 | Eml5 | -1.27 |
| RIKEN cDNA E230008O15 gene | E230008O15Rik | -1.27 |
| matrix metallopeptidase 19 | Mmp19 | -1.27 |
| RIKEN cDNA 4930523C07 gene | 4930523C07Rik | -1.28 |
| l(3)mbt-like 2 (Drosophila) | L3mbtl2 | -1.28 |
| zinc finger protein 329 | Zfp329 | -1.28 |
| early growth response 2 | Egr2 | -1.28 |
| C-type lectin domain family 7, member a | Clec7a | -1.29 |
| ribosomal protein S23 | Rps23 | -1.29 |
| RIKEN cDNA B230214N19 gene | B230214N19Rik | -1.29 |
| CCR4-NOT transcription complex, subunit 6-like | Cnot6l | -1.30 |
| suppression inducing transmembrane adaptor 1 | Sit1 | -1.30 |
| RIKEN cDNA B130040O20 gene | B130040O20Rik | -1.30 |
| RAB19, member RAS oncogene family | Rab19 | -1.30 |
| meiotic nuclear divisions 1 homolog (S. cerevisiae) | Mnd1 | -1.30 |
| sulfotransferase family, cytosolic, 1C, member 2 | Sult1c2 | -1.31 |
| RIKEN cDNA 9930017N22 gene | 9930017N22Rik | -1.32 |
| RIKEN cDNA D130020G16 gene | D130020G16Rik | -1.33 |
| DENN/MADD domain containing 1B | Dennd1b | -1.33 |
| family with sequence similarity 19, member A3 | Fam19a3 | -1.33 |
| keratin 76 | Krt76 | -1.33 |
| olfactory receptor 373 | Olfr373 | -1.34 |
| interleukin 11 | Il11 | -1.34 |
| RIKEN cDNA 4732419C18 gene | 4732419C18Rik | -1.34 |
| RIKEN cDNA 9430019C24 gene | 9430019C24Rik | -1.35 |
| bromodomain adjacent to zinc finger domain 1A | Baz1a | -1.35 |
| ribosomal protein L38 | Rpl38 | -1.35 |
| transformer 2 alpha homolog (Drosophila) | Tra2a | -1.35 |
| teashirt zinc finger family member 3 | Tshz3 | -1.36 |
| caspase 4, apoptosis-related cysteine peptidase | Casp4 | -1.36 |
| kelch-like 6 (Drosophila) | Klhl6 | -1.36 |
| predicted gene 14410 | Gm14410 | -1.37 |
| protamine 2 | Prm2 | -1.37 |
| ribosomal protein L41 | Rpl41 | -1.37 |
| CD7 antigen | Cd7 | -1.37 |
| RIKEN cDNA 4932425I24 gene | 4932425I24Rik | -1.38 |
| Shc SH2-domain binding protein 1 | Shcbp1 | -1.38 |
| expressed sequence AI463229 | AI463229 | -1.39 |
| complement component 1, r subcomponent B | C1rb | -1.39 |
| transmembrane protein 55A | Tmem55a | -1.39 |
| protein tyrosine phosphatase, receptor type, G | Ptprg | -1.40 |
| hypothetical protein LOC100049077 | LOC100049077 | -1.40 |
| zinc finger protein 160 | Zfp160 | -1.40 |
| TBC1 domain containing kinase | Tbck | -1.41 |
| alkylglycerol monooxygenase | Agmo | -1.42 |
| nanos homolog 3 (Drosophila) | Nanos3 | -1.42 |
| RIKEN cDNA A230057D06 gene | A230057D06Rik | -1.43 |
| cytochrome P450, family 2, subfamily c, polypeptide 65 | Cyp2c65 | -1.44 |
| NLR family, CARD domain containing 3 | Nlrc3 | -1.45 |
| DNA segment, Chr 19, ERATO Doi 409, expressed | D19Ertd409e | -1.45 |
| RIKEN cDNA B130063F10 gene | B130063F10Rik | -1.46 |
| fos-like antigen 2 | Fosl2 | -1.46 |
| DiGeorge syndrome critical region gene 8 | Dgcr8 | -1.46 |
| natriuretic peptide type B | Nppb | -1.47 |
| RIKEN cDNA 4632427E13 gene | 4632427E13Rik | -1.48 |
| RIKEN cDNA 8430437O03 gene | 8430437O03Rik | -1.49 |
| lymphocyte antigen 9 | Ly9 | -1.50 |
| DDB1 and CUL4 associated factor 17 | Dcaf17 | -1.50 |
| H19 fetal liver mRNA | H19 | -1.51 |
| RIKEN cDNA 2810403D21 gene | 2810403D21Rik | -1.51 |
| RIKEN cDNA 1700121C10 gene | 1700121C10Rik | -1.52 |
| WNT1 inducible signaling pathway protein 2 | Wisp2 | -1.53 |
| cathepsin E | Ctse | -1.54 |
| microphthalmia-associated transcription factor | Mitf | -1.54 |
| lin-9 homolog (C. elegans) | Lin9 | -1.54 |
| annexin A7 | Anxa7 | -1.55 |
| LMBR1 domain containing 2 | Lmbrd2 | -1.55 |
| CD5 antigen | Cd5 | -1.55 |
| olfactory receptor 1366 | Olfr1366 | -1.57 |
| FYN binding protein | Fyb | -1.57 |
| SPC25, NDC80 kinetochore complex component, homolog (S. cerevisiae) | Spc25 | -1.58 |
| 3-oxoacid CoA transferase 1 | Oxct1 | -1.59 |
| 3-phosphoinositide dependent protein kinase 1 | Pdpk1 | -1.59 |
| deoxynucleotidyltransferase, terminal | Dntt | -1.62 |
| CD48 antigen | Cd48 | -1.63 |
| RAD54 like (S. cerevisiae) | Rad54l | -1.63 |
| RIKEN cDNA E430024C06 gene | E430024C06Rik | -1.65 |
| ras homolog gene family, member H | Rhoh | -1.65 |
| homeobox B7 | Hoxb7 | -1.65 |
| H6 homeobox 2 | Hmx2 | -1.65 |
| RIKEN cDNA 2210010C17 gene | 2210010C17Rik | -1.68 |
| Ras association (RalGDS/AF-6) domain family (N-terminal) member 7 | Rassf7 | -1.69 |
| RIKEN cDNA A830082K12 gene | A830082K12Rik | -1.69 |
| collagen, type IV, alpha 5 | Col4a5 | -1.73 |
| RIKEN cDNA E130120C16 gene | E130120C16Rik | -1.75 |
| pterin 4 alpha carbinolamine dehydratase/dimerization cofactor of hepatocyte nuclear factor 1 alpha (TCF1) 2 | Pcbd2 | -1.76 |
| DNA (cytosine-5-)-methyltransferase 3-like | Dnmt3l | -1.76 |
| RIKEN cDNA 3100003M19 gene | 3100003M19Rik | -1.76 |
| RIKEN cDNA 3110080O07 gene | 3110080O07Rik | -1.78 |
| predicted gene 10825 | Gm10825 | -1.79 |
| lectin, mannose-binding 1 like | Lman1l | -1.79 |
| chemokine (C-C motif) receptor 9 | Ccr9 | -1.79 |
| HORMA domain containing 1 | Hormad1 | -1.80 |
| secretoglobin, family 3A, member 2 | Scgb3a2 | -1.81 |
| PIN2/TERF1 interacting, telomerase inhibitor 1 | Pinx1 | -1.82 |
| RIKEN cDNA A430093F15 gene | A430093F15Rik | -1.83 |
| N-acetyltransferase 3 | Nat3 | -1.84 |
| RIKEN cDNA 2810442I21 gene | 2810442I21Rik | -1.85 |
| apolipoprotein C-II | Apoc2 | -1.85 |
| cytidine monophospho-N-acetylneuraminic acid hydroxylase | Cmah | -1.87 |
| RIKEN cDNA D730045B01 gene | D730045B01Rik | -1.89 |
| zinc finger protein 369 | Zfp369 | -1.93 |
| transmembrane 4 superfamily member 4 | Tm4sf4 | -1.93 |
| Rho GTPase activating protein 4 | Arhgap4 | -1.98 |
| RIKEN cDNA 6720473M08 gene | 6720473M08Rik | -2.01 |
| programmed cell death 1 | Pdcd1 | -2.06 |
| hypothetical LOC100505358 | LOC100505358 | -2.15 |
| transmembrane protein 221 | Tmem221 | -2.19 |
| predicted gene 3134 | Gm3134 | -2.25 |
| helicase, lymphoid specific | Hells | -2.35 |
| expressed sequence AU022855 | AU022855 | -2.42 |
| aquaporin 5 | Aqp5 | -2.49 |
| chitinase 3-like 3 | Chi3l3 | -2.52 |
| cystatin F (leukocystatin) | Cst7 | -2.56 |
| muscle-related coiled-coil protein | Murc | -2.74 |
| histidine ammonia lyase | Hal | -2.79 |
| predicted gene, 16532 | Gm16532 | -2.92 |
| acid phosphatase, prostate | Acpp | -3.40 |
