# Supplementary material for: Transcriptomic Analysis of the Effects of a Fish Oil Enriched Diet on Murine Brains
Source: PLoS One. 2014 Mar 14;9(3):e90425. doi: 10.1371/journal.pone.0090425 (PMC3954562; doi:10.1371/journal.pone.0090425)
Supplement: Table S3 — Complete list of biofunction categories enriched by 1,142 genes. For individual functional categories, corresponding range of p values and the number of enriching genomic members are reported. (DOCX) [file pone.0090425.s005.docx]

# Table S3. Complete list of biofunction categories enriched by 1142 genes

| **Biofunction categories** | **p-value range** | **Number of Molecules** |
| --- | --- | --- |
| Cell-To-Cell Signaling and Interaction | 1.04E-3 to 1.46E-11 | 176 |
| Organismal Injury and Abnormalities | 1.73E-3 to 1.89E-8 | 65 |
| Nervous System Development and Function | 1.04E-3 to 7.83E-7 | 150 |
| Tissue Development | 1.04E-3 to 1.79E-7 | 202 |
| Cell Death | 1.25E-3 to 1.79E-7 | 89 |
| Cellular Development | 1.06E-3 to 3.03E-7 | 179 |
| Metabolic Disease | 1.73E-3 to 2.27E-6 | 211 |
| Cell Signaling | 1.01E-3 to 3.48E-6 | 97 |
| Molecular Transport | 1.01E-3 to 3.48E-6 | 169 |
| Vitamin and Mineral Metabolism | 1.01E-3 to 4.04E-6 | 75 |
| Cardiovascular Disease | 1.03E-3 to 8.17E-6 | 180 |
| Genetic Disorder | 1.09E-3 to 4.24E-6 | 432 |
| Hematological System Development and Function | 1.04E-3 to 6.18E-6 | 112 |
| Immune Cell Trafficking | 1.04E-3 to 6.18E-6 | 80 |
| Infalmmatory Response | 3.03E-3 to 6.18E-6 | 108 |
| Organ Morphology | 2.10E-3 to 6.94E-6 | 21 |
| Hematological Disease | 1.03E-3 to 1.18E-5 | 165 |
| Neurological Disease | 1.01E-3 to 5.69E-5 | 260 |
| Immunological Disease | 1.07E-3 to 9.63E-5 | 193 |
| Inflammatory Disease | 1.60E-3 to 2.06E-5 | 111 |
| Embryonic Development | 1.54E-3 to 2.23E-5 | 118 |
| Organ Development | 2.19E-3 to 2.23E-5 | 101 |
| Organismal Development | 1.73E-3 to 2.23E-5 | 149 |
| Tissue Morphology | 1.44E-3 to 3.96E-5 | 109 |
| Behavior | 1.17E-3 to 3.19E-5 | 74 |
| Infectious Disease | 1.74E-3 to 3.51E-5 | 47 |
| Connective Tissue Development and Function | 1.27E-3 to 3.76E-5 | 55 |
| Skeletal and Muscular System Development and Function | 1.58E-3 to 3.76E-5 | 52 |
| Cellular Compromise | 1.73E-3 to 3.90E-5 | 34 |
| Cellular Growth and Proliferation | 1.61E-3 to 5.90E-5 | 184 |
| Endocrine System Disorders | 1.73E-3 to 4.57E-5 | 180 |
| Cardiovascular System Development and Function | 1.73E-3 to 6.54E-5 | 55 |
| Carbohydrate Metabolism | 1.75E-3 to 7.29E-5 | 33 |
| Dermatological Diseases and Conditions | 1.73E-3 to 9.63E-5 | 98 |
| Nucleic Acid Metabolism | 1.08E-3 to 1.05E-5 | 12 |
| Small Molecule Biochemistry | 1.10E-3 to 2.10E-4 | 107 |
| Gastrointestinal Disease | 1.61E-3 to 1.37E-4 | 232 |
| Cellular Movement | 1.61E-3 to 7.36E-4 | 123 |
| Respiratory Disease | 1.74E-3 to 3.71E-5 | 23 |
| Antigen Presentation | 2.53E-3 to 8.97E-4 | 27 |
| Hepatic System Disease | 1.61E-3 to 7.14E-4 | 50 |
| Cell-mediated Immune Response | 1.73E-3 to 2.12E-5 | 28 |
| Cellular Function and Maintenance | 1.04E-3 to 2.12E-4 | 50 |
| Hematopoiesis | 2.10E-3 tp2.12E-4 | 29 |
| Lymphoid Tissue Structure and Development | 1.74E-3 to 2.12E-4 | 23 |
| Lipid Metabolism | 1.06E-3 to 2.24E-4 | 75 |
| Hair and Skin Development and Function | 3.18E-3 to 2.38E-4 | 24 |
| Humoral Immune Response | 1.90E-3 to 3.71E-4 | 20 |
| Skeletal and Muscular Disorders | 1.26E-3 to 5.10E-4 | 67 |
| Gene Expression | 1.12E-3 to 8.14E-4 | 45 |
| Endocrine System Development and Function | 1.34E-3 to 5.50E-4 | 17 |
| Post-Translational Modification | 5.70E-4 to 5.70E-4 | 20 |
| Nutritional Disease | 2.94E-3 to 8.53E-4 | 26 |
| Psychological Disorders | 6.54E-4 to 1.09E-3 | 17 |
| Amino Acid Metabolism | 1.04E-3 to 7.94E-4 | 24 |
| Cell Cycle | 1.12E-3 to 8.14E-4 | 14 |
| Hypersensitivity Response | 8.14E-4 to 8.14E-4 | 9 |
| Antimicrobial Response | 8.95E-4 to 8.95E-4 | 3 |
| Cell Morphology | 1.36E-3 to 8.95E-4 | 50 |
| Cellular Assembly and Organization | 1.04E-3 to 8.95E-4 | 25 |
| Tumor Morphology | 8.95E-4 to 8.95E-4 | 3 |
| Drug Metabolism | 1.21E-3 to 2.10E-3 | 15 |
| Renal and Urological System Development and Function | 1.61E-3 to 1.27E-3 | 11 |
| Ophthalmic Disease | 1.60E-3 to 1.60E-3 | 8 |
| DNA Replication, Recombination, and Repair | 1.61E-3 to 2.10E-3 | 6 |
| Free Radical Scavenging | 1.72E-3 to 2.47E-3 | 31 |
| Developmental Disorder | 1.72E-3 to 2.66E-3 | 79 |
| Auditory and Vestibular System Development and Function | 2.10E-3 to 2.10E-3 | 2 |
| Cancer | 2.10E-3 to 2.63E-3 | 23 |
| Connective Tissue Disorders | 2.10E-3 to 2.94E-3 | 25 |
| RNA Damage and Repair | 2.10E-3 to 2.10E-3 | 2 |
| Visual System Development and Function | 2.10E-3 to 2.10E-3 | 2 |
| Protein Synthesis | 2.20E-3 to 2.10E-3 | 5 |
| Renal and Urological Disease | 2.38E-3 to 2.10E-3 | 10 |
| Digestive System Development and Function | 3.01E-3 to 2.10E-3 | 8 |
