# Supplementary material for: Transcriptomic Analysis of the Effects of a Fish Oil Enriched Diet on Murine Brains
Source: PLoS One. 2014 Mar 14;9(3):e90425. doi: 10.1371/journal.pone.0090425 (PMC3954562; doi:10.1371/journal.pone.0090425)
Supplement: Table S4 — The brain regions enriched by the genes mined by present study relevant to “Neurological disease”, “Nervous system development and function”, “Inflammatory response” and “Cell death”. (DOCX) [file pone.0090425.s006.docx]

# Table S4. The brain regions enriched by the genes mined by present study relevant to “Neurological disease”, “Nervous system development and function”, “Inflammatory response” and “Cell death”

| **Brain region** | **Entrez ID** | **Gene Symbol** | **Log Ratio** | **Neurological Disease; Nervous system development and functions; Inflammatory response, cell death** |
| --- | --- | --- | --- | --- |
| Olfactory bulb | 16504 | KCNC3 | 0.97 | MvD |
|  | 114142 | FOXP2 | 0.96 | MvD, PD |
|  | 12801 | CNR1 | 0.88 | AD, MvD, PD; SyT, Ngs (GoN, DCNS, DoN); ImRes |
|  | 213262 | FSTL5 | 0.83 | AD, MvD, PD |
|  | 140919 | SLC17A6 | 0.83 | MvD, PD |
|  | 18213 | NTRK3 | 0.8 | AD, MvD, PD; Ngs (Ntg, GoN, FPMP, DoN); Apop |
|  | 11550 | ADRA1D | 0.54 | AD, MvD, PD |
|  | 16522 | KCNJ6 | 0.41 | MvD, PD |
|  | 14680 | GNAL | 0.36 | MvD |
| Oculomotor nucleus | 20508 | SLC18A3 | 1.2 | MvD |
|  | 63993 | SLC5A7 | 0.84 | AD; SyT |
|  | 12310 | CALCB | 1.33 | ImRes |
| Somatosensory areas | 16504 | KCNC3 | 0.97 | MvD |
| Retrosplenial area | 110893 | SLC8A3 | 0.98 | MvD, PD; SyT |
| Amygdala  (including cortical, central and medial) | 140919 | SLC17A6 | 0.83 | MvD, PD |
|  | 18125 | NOS1 | 0.54 | AD, MvD, PD; Ngs (Ntg, FPMP, DoN) |
|  | 18753 | PRKCD | 0.31 | AD; Ngs (GoN); Apop, ImRes |
|  | 21834 | THRB | 0.71 | MvD, PD; Ngs (DCNS) |
| Dentate gyrus | 18610 | PDYN | 0.67 | MvD |
| Isocortex | 14680 | GNAL | 0.36 | MvD |
| Nucleus accumbens | 226922 | KCNQ5 | 0.69 | MvD, PD |
|  | 14407 | GABRG3 | 0.46 | AD, MvD, PD |
| Hippocampus (including CA1, CA2 and CA3, all pyramidal regions) | 226922 | KCNQ5 | 0.69 | MvD, PD |
|  | 18753 | PRKCD | 0.31 | AD; Ngs (GoN); Apop, ImRes |
| Thalamus (including ventral posterior complex, paraventricular nucleus, reticular nucleus) | 16521 | KCNJ5 | 1.45 | AD |
|  | 16504 | KCNC3 | 0.97 | MvD |
|  | 11550 | ADRA1D | 0.54 | AD, MvD, PD |
|  | 17536 | MEIS2 | -0.5 | MvD |
| Subthalamic nucleus | 14680 | GNAL | 0.36 | MvD |
| Zona incerta | 20660 | SORL1 | 1.32 | AD, MvD |
|  | 16504 | KCNC3 | 0.97 | MvD |
|  | 17536 | MEIS2 | -0.5 | MvD |
| Colliculus (superior and inferior) | 14680 | GNAL | 0.36 | MvD |
| Midbrian trigeminal nucleus | 213783 | PLEKHG1 | 0.64 | AD |
|  | 18053 | NGFR | 1.15 | AD; Ngs (Ntg, FPMP, GoN, DCNS, DoN); Apop |
| Cerebellar cortex (purkinje and granular layer) | 110893 | SLC8A3 | 0.98 | MvD, PD |
|  | 16504 | KCNC3 | 0.97 | MvD |
|  | 319924 | APBA1 | 0.51 | AD; SyT |
|  | 14804 | GRID2 | 0.32 | AD, MvD; SyT |
|  | 13653 | EGR1 | -0.61 | MvD; ImRes, Apop |
| Cerebellar nuclei | 140919 | SLC17A6 | 0.83 | MvD, PD |
|  | 58178 | SORCS1 | 0.45 | AD |
|  | 17536 | MEIS2 | -0.5 | MvD |
|  | 110893 | SLC8A3 | 0.98 | SyT |
| Entorhinal area | 65254 | DPYSL5 | 1.02 | Ngs (GoN) |
|  | 22422 | WNT7B | 0.7 | Ngs (Ntg, FPMP) |
| Triangular nucleus of septum | 20562 | SLIT1 | 0.74 | Ngs (Ntg, GoN, FPMP, DCNS) |
