# Supplementary material for: Transcriptomic Analysis of the Effects of a Fish Oil Enriched Diet on Murine Brains
Source: PLoS One. 2014 Mar 14;9(3):e90425. doi: 10.1371/journal.pone.0090425 (PMC3954562; doi:10.1371/journal.pone.0090425)
Supplement: Table S5 — The genomic candidates associated with apoptosis (Apop) and immune response (ImRes). The genes are identified by the Entrez IDs and gene symbols. The log ratio indicates the log1.5 transformed of the average transcriptomic expressions from FD-/SD-fed mice. (DOCX) [file pone.0090425.s007.docx]

## Table S5. The genomic candidates associated with apoptosis (Apop) and immune response (ImRes). The genes are identified by the Entrez ID and gene symbol. The log ratio indicates the log1.5 transformed of the average transcriptomic expression from FD- / SD-fed mice.

| **Entrez ID** | **Gene Symbol** | **Log Ratio** | **Apoptosis and Immune Response** |
| --- | --- | --- | --- |
| 11555 | ADRB2 | 0.47 | ImRes |
| 11682 | ALK | 0.54 | Apop |
| 12014 | BACH2 | -0.49 | Apop, ImRes |
| 12015 | BAD | 0.58 | Apop, ImRes |
| 12048 | BCL2L1 | 0.44 | Apop, ImRes |
| 12161 | BMP6 | 0.75 | ImRes |
| 12258 | SERPING1 | 0.38 | ImRes |
| 12265 | CIITA | 0.49 | Apop, ImRes |
| 12310 | CALCB | 1.33 | ImRes |
| 12363 | CASP4 | -0.96 | Apop, ImRes |
| 12395 | RUNX1T1 | -0.59 | Apop |
| 12481 | CD2 | -0.58 | Apop, ImRes |
| 12487 | CD28 | -0.35 | Apop, ImRes |
| 12502 | CD3G | -0.87 | ImRes |
| 12504 | CD4 | 0.62 | Apop, ImRes |
| 12506 | CD48 | -0.47 | Apop, ImRes |
| 12507 | CD5 | -0.67 | Apop |
| 12516 | CD7 | -0.23 | ImRes |
| 12519 | CD80 | -1.91 | Apop, ImRes |
| 12649 | CHEK1 | -1.06 | Apop |
| 12655 | Chi3l3/Chi3l4 | -0.98 | ImRes |
| 12769 | CCR9 | -0.90 | ImRes |
| 12774 | CCR5 | 0.99 | Apop, ImRes |
| 12776 | CCR8 | -1.25 | Apop, ImRes |
| 12801 | CNR1 | 0.88 | ImRes |
| 12981 | CSF2 | -0.43 | Apop, ImRes |
| 13011 | CST7 | -0.85 | ImRes |
| 13033 | CTSD | 0.35 | ImRes |
| 13034 | CTSE | -0.66 | ImRes |
| 13039 | CTSL2 | -0.49 | ImRes |
| 13190 | DCT | 2.76 | ImRes |
| 13616 | EDN3 | 0.63 | ImRes |
| 13653 | EGR1 | -0.61 | Apop, ImRes |
| 13654 | EGR2 | -0.69 | Apop, ImRes |
| 14058 | F10 | -0.87 | ImRes |
| 14128 | FCER2 | -1.20 | Apop, ImRes |
| 14173 | FGF2 | -0.87 | Apop, ImRes |
| 14264 | FMOD | 0.67 | ImRes |
| 14612 | GJA4 | 0.80 | ImRes |
| 14960 | HLA-DQA1 | 1.27 | ImRes |
| 14961 | HLA-DQB1 (includes others) | 0.77 | ImRes |
| 15061 | IFI44L | -1.01 | ImRes |
| 15234 | HGF | -0.38 | Apop, ImRes |
| 16153 | IL10 | -0.97 | Apop, ImRes |
| 16176 | IL1B | -0.44 | Apop, ImRes |
| 16409 | ITGAM | 0.49 | ImRes |
| 16439 | ITPR2 | -0.33 | Apop |
| 16590 | KIT | 0.66 | Apop, ImRes |
| 16591 | KL | 1.32 | ImRes |
| 16621 | KLKB1 | -1.11 | ImRes |
| 16637 | Klra4 (includes others) | -0.77 | ImRes |
| 16678 | KRT1 | 0.61 | ImRes |
| 16803 | LBP | 0.72 | ImRes |
| 16985 | LSP1 | 0.50 | Apop, ImRes |
| 17057 | Klrb1c (includes others) | -2.42 | ImRes |
| 17110 | Lyz1/Lyz2 | 0.63 | ImRes |
| 17224 | Mcpt1 | -0.89 | ImRes |
| 17441 | MOG | 1.01 | ImRes |
| 17528 | MPZ | 0.29 | ImRes |
| 18019 | NFATC2 | 0.27 | Apop, ImRes |
| 18053 | NGFR | 1.15 | Apop |
| 18106 | CD244 | -0.74 | ImRes |
| 18126 | NOS2 | -0.32 | ImRes |
| 18213 | NTRK3 | 0.80 | Apop |
| 18386 | OPRD1 | -0.60 | ImRes |
| 18566 | PDCD1 | -0.58 | Apop, ImRes |
| 18607 | PDPK1 | -0.78 | Apop |
| 18646 | PRF1 | 0.86 | Apop, ImRes |
| 18654 | PGF | 0.61 | Apop |
| 18753 | PRKCD | 0.31 | Apop, ImRes |
| 19015 | PPARD | 0.55 | ImRes |
| 19108 | PRKX | -0.37 | ImRes |
| 19109 | PRL | -2.23 | Apop, ImRes |
| 19368 | Raet1a | -0.44 | ImRes |
| 19697 | RELA | 0.34 | Apop, ImRes |
| 19877 | ROCK1 | -0.61 | ImRes |
| 20310 | CXCL3 | -0.30 | ImRes |
| 20387 | SFTPA1 | 0.65 | ImRes |
| 20655 | SOD1 | -0.32 | Apop, ImRes |
| 20657 | SOD3 | 1.08 | ImRes |
| 21336 | TACR1 | 0.69 | ImRes |
| 21673 | DNTT | -0.62 | ImRes |
| 21819 | TG | -0.59 | ImRes |
| 21926 | TNF | -1.21 | Apop, ImRes |
| 22029 | TRAF1 | -0.46 | Apop |
| 22361 | VNN1 | -1.54 | ImRes |
| 22376 | WAS | -0.39 | Apop, ImRes |
| 23845 | CLEC5A | -0.74 | ImRes |
| 23880 | FYB | -0.69 | ImRes |
| 23957 | NR0B2 | -0.63 | ImRes |
| 24010 | IK | -0.22 | ImRes |
| 26419 | MAPK8 | -0.35 | Apop, ImRes |
| 27052 | AOAH | -0.42 | ImRes |
| 50930 | TNFSF14 | -0.81 | ImRes |
| 50931 | IL27RA | -0.81 | ImRes |
| 54485 | DLL4 | 0.51 | Apop, ImRes |
| 56066 | CXCL11 | -0.46 | ImRes |
| 57757 | PGLYRP2 | -0.74 | ImRes |
| 58861 | CYSLTR1 | -0.34 | ImRes |
| 58992 | F12 | 0.87 | ImRes |
| 69188 | MLL5 | -0.68 | ImRes |
| 70101 | Cyp4f16/Gm9705 | 0.27 | ImRes |
| 71884 | CHIT1 | -1.18 | ImRes |
| 140806 | IL25 | 1.02 | ImRes |
| 192236 | HPS1 | 0.48 | ImRes |
| 225825 | CD226 | -0.31 | ImRes |
| 232946 | BLOC1S3 | 0.94 | ImRes |
| 268857 | NLRC3 | -0.63 | ImRes |
| 270151 | NLRX1 | 0.69 | ImRes |
| 329244 | IL19 | -0.32 | Apop, ImRes |
